# Report on the Development of the Advanced Encryption Standard (AES)

**DOI:** 10.6028/jres.106.023

**Published:** 2001-06-01

**Authors:** James Nechvatal, Elaine Barker, Lawrence Bassham, William Burr, Morris Dworkin, James Foti, Edward Roback

**Affiliations:** National Institute of Standards and Technology, Gaithersburg, MD 20899-8930

**Keywords:** Advanced Encryption Standard (AES), cryptography, cryptanalysis, cryptographic algorithms, encryption, Rijndael

## Abstract

In 1997, the National Institute of Standards and Technology (NIST) initiated a process to select a symmetric-key encryption algorithm to be used to protect sensitive (unclassified) Federal information in furtherance of NIST’s statutory responsibilities. In 1998, NIST announced the acceptance of 15 candidate algorithms and requested the assistance of the cryptographic research community in analyzing the candidates. This analysis included an initial examination of the security and efficiency characteristics for each algorithm. NIST reviewed the results of this preliminary research and selected MARS, RC™, Rijndael, Serpent and Twofish as finalists. Having reviewed further public analysis of the finalists, NIST has decided to propose Rijndael as the Advanced Encryption Standard (AES). The research results and rationale for this selection are documented in this report.

“I’ll do the [S]quare thing.”

—G.B. McCutcheon (1917)

## 1. Overview of the Development Process for the Advanced Encryption Standard and Summary of Round 2 Evaluations

The National Institute of Standards and Technology (NIST) has been working with the international cryptographic community to develop an Advanced Encryption Standard (AES). The overall goal is to develop a Federal Information Processing Standard (FIPS) that specifies an encryption algorithm capable of protecting sensitive (unclassified) government information well into the twenty-first century. NIST expects that the algorithm will be used by the U.S. Government and, on a voluntary basis, by the private sector.

The competition among the finalists was very intense, and NIST selected Rijndael as the proposed AES algorithm at the end of a very long and complex evaluation process. This report describes that process and summarizes many of the characteristics of the algorithms that were identified during the public evaluation periods. The following sections provide an overview of the AES development followed by a discussion of specific analysis details.

### 1.1 Background

On January 2, 1997, NIST announced the initiation of an effort to develop the AES [31] and made a formal call for algorithms on September 12, 1997 [32]. The call indicated NIST’s goal that the AES would specify an unclassified, publicly disclosed encryption algorithm, available royalty-free, worldwide. At a minimum, the algorithm would have to implement symmetric key cryptography as a block cipher and support a block size of 128 bits and key sizes of 128, 192, and 256 bits.

On August 20, 1998, NIST announced 15 AES candidate algorithms at the First AES Candidate Conference (AES1) and solicited public comments on the candidates [33]. Industry and academia submitters from twelve countries proposed the fifteen algorithms. A Second AES Candidate Conference (AES2) was held in March 1999 to discuss the results of the analysis that was conducted by the international cryptographic community on the candidate algorithms. In August 1999, NIST announced its selection of five finalist algorithms from the fifteen candidates. The selected algorithms were MARS, RC6™, Rijndael, Serpent and Twofish.

### 1.2 Overview of the Finalists

The five finalists are iterated block ciphers: they specify a transformation that is iterated a number of times on the data block to be encrypted or decrypted. Each iteration is called a round, and the transformation is called the round function. The data block to be encrypted is called the plaintext; the encrypted plaintext is called the ciphertext. For decryption, the ciphertext is the data block to be processed. Each finalist also specifies a method for generating a series of keys from the original user key; the method is called the key schedule, and the generated keys are called subkeys. The round functions take distinct subkeys as input along with the data block.

For each finalist, the very first and last cryptographic operations are some form of mixing of subkeys with the data block. Such mixing of secret subkeys prevents an adversary who does not know the keys from even beginning to encrypt the plaintext or decrypt the ciphertext. Whenever this subkey mixing does not naturally occur as the initial step of the first round or the final step of the last round, the finalists specify the subkey mixing as an extra step called pre- or post-whitening.

There are other common technical features of the finalists. Four of the finalists specify substitution tables, called S-boxes: an *A×B* bit S-box replaces *A* bit inputs with *B* bit outputs. Three of the finalists specify variations on a structure for the round function, called the Feistel structure. In the classic Feistel structure, half of the data block is used to modify the other half of the data block, and then the halves are swapped. The two finalists that do not use a Feistel structure process the entire data block in parallel during each round using substitutions and linear transformations; thus, these two finalists are examples of substitution-linear transformation networks.

Below is a summary of each of the finalist candidates in alphabetical order; profiles and Round 2 assessments are provided in subsequent sections of this report.

MARS [15] has several layers: key addition^1^ as pre-whitening, eight rounds of unkeyed forward mixing, eight rounds of keyed forward transformation, eight rounds of keyed backwards transformation, eight rounds of unkeyed backwards mixing, and key subtraction as post-whitening. The 16 keyed transformations are called the cryptographic core. The unkeyed rounds use two 8×32 bit S-boxes, addition, and the XOR operation. In addition to those elements, the keyed rounds use 32 bit key multiplication, data-dependent rotations, and key addition. Both the mixing and the core rounds are modified Feistel rounds in which one fourth of the data block is used to alter the other three fourths of the data block. MARS was submitted by the International Business Machines Corporation (IBM).

RC6 [75] is a parameterized family of encryption ciphers that essentially use the Feistel structure; 20 rounds were specified for the AES submission. The round function of RC6 uses variable rotations that are regulated by a quadratic function of the data. Each round also includes 32 bit modular multiplication, addition, XOR (i.e., exclusive-or), and key addition. Key addition is also used for pre- and post-whitening. RC6 was submitted to the AES development effort by RSA Laboratories.

Rijndael [22] is a substitution-linear transformation network with 10, 12, or 14 rounds, depending on the key size. A data block to be processed using Rijndael is partitioned into an array of bytes, and each of the cipher operations is byte-oriented. Rijndael’s round function consists of four layers. In the first layer, an 8×8 S-box is applied to each byte. The second and third layers are linear mixing layers, in which the rows of the array are shifted, and the columns are mixed. In the fourth layer, subkey bytes are XORed into each byte of the array. In the last round, the column mixing is omitted. Rijndael was submitted by Joan Daemen (Proton World International) and Vincent Rijmen (Katholieke Universiteit Leuven).

Serpent [4] is a substitution-linear transformation network consisting of 32 rounds. Serpent also specifies non-cryptographic initial and final permutations that facilitate an alternative mode of implementation called the bitslice mode. The round function consists of three layers: the key XOR operation, 32 parallel applications of one of the eight specified 4×4 S-boxes, and a linear transformation. In the last round, a second layer of key XOR replaces the linear transformation. Serpent was submitted by Ross Anderson (University of Cambridge), Eli Biham (Technion), and Lars Knudsen (University of California San Diego).

Twofish [83] is a Feistel network with 16 rounds. The Feistel structure is slightly modified using 1 bit rotations. The round function acts on 32 bit words with four key-dependent 8×8 S-boxes, followed by a fixed 4×4 maximum distance separable matrix over GF(2^8^), a pseudo-Hadamard transform, and key addition. Twofish was submitted by Bruce Schneier, John Kelsey, and Niels Ferguson (Counterpane Internet Security, Inc.), Doug Whiting (Hi/fn, Inc.), David Wagner (University of California Berkeley), and Chris Hall (Princeton University).

In announcing the finalists, NIST again solicited public review and comment on the algorithms [34]. These algorithms received further analysis during a second, more in-depth review period, and the Third AES Candidate Conference (AES3) was held in April 2000 to present and discuss much of that analysis. The public comment period for reviewing the finalist algorithms closed on May 15, 2000. At that time, NIST’s AES team conducted a thorough review of all of the public comments and analyses of the finalists.

### 1.3 Evaluation Criteria

In the September 1997 call for candidate algorithms [32], NIST specified the overall evaluation criteria that would be used to compare the candidate algorithms. These criteria were developed from public comments to Ref. [31] and from the discussions at a public AES workshop held on April 15, 1997 at NIST.

The evaluation criteria were divided into three major categories: 1) Security, 2) Cost, and 3) Algorithm and Implementation Characteristics. Security was the most important factor in the evaluation and encompassed features such as resistance of the algorithm to cryptanalysis, soundness of its mathematical basis, randomness of the algorithm output, and relative security as compared to other candidates.

Cost was a second important area of evaluation that encompassed licensing requirements, computational efficiency (speed) on various platforms, and memory requirements. Since one of NIST’s goals was that the final AES algorithm be available worldwide on a royalty-free basis, public comments were specifically sought on intellectual property claims and any potential conflicts. The speed of the algorithm on a variety of platforms needed to be considered. During Round 1, the focus was primarily on the speed associated with 128 bit keys. During Round 2, hardware implementations and the speeds associated with the 192 bit and 256 bit key sizes were addressed. Memory requirements and software implementation constraints for software implementations of the candidates were also important considerations.

The third area of evaluation was algorithm and implementation characteristics such as flexibility, hard ware and software suitability, and algorithm simplicity. Flexibility includes the ability of an algorithm:

- To handle key and block sizes beyond the minimum that must be supported,
- To be implemented securely and efficiently in many different types of environments, and
- To be implemented as a stream cipher, hashing algorithm, and to provide additional cryptographic services.

It must be feasible to implement an algorithm in both hardware and software, and efficient firmware implementations were considered advantageous. The relative simplicity of an algorithm’s design was also an evaluation factor.

During Rounds 1 and 2, it became evident that the various issues being analyzed and discussed often crossed into more than one of the three main criteria headings. Therefore, the criteria of cost and algorithm characteristics were considered together as secondary criteria, after security. This report addresses the criteria listed above, as follows:

**Table:** 

Security:	Sects. 3.2 and 3.6.
Cost:	Sects. 3.3, 3.4, 3.5, 3.7, 3.8, 3.10, and 4.
Algorithm Characteristics:	Sects. 3.3, 3.4, 3.5, 3.6, 3.8, 3.9, and 3.10.

### 1.4 Results from Round 2

The Round 2 public review extended from the official announcement of the five AES finalists on August 20, 1999 until the official close of the comment period on May 15, 2000. During Round 2, many members of the global cryptographic community supported the AES development effort by analyzing and testing the five AES finalists.

NIST facilitated and focused the analysis of the finalists by providing an electronic discussion forum and home page. The public and NIST used the electronic forum [1] to discuss the finalists and relevant AES issues, inform the public of new analysis results, etc. The AES home page [2] served as a tool to disseminate information such as algorithm specifications and source code, AES3 papers, and other Round 2 public comments.

Thirty-seven papers were submitted to NIST for consideration for AES3. Twenty-four of those papers were presented at AES3 as part of the formal program, and one of the remaining papers was presented during an informal session at AES3. All of the submitted papers were posted on the AES home page [2] several weeks prior to AES3 in order to promote informed discussions at the conference.

AES3 gave members of the international cryptographic community an opportunity to present and discuss Round 2 analysis and other important topics relevant to the AES development effort. A summary of AES3 presentations and discussions will be available in Ref. [29]. In addition to the AES3 papers, NIST received 136 sets of public comments on the finalists during Round 2 in the form of submitted papers, email comments and letters. All of these comments were made publicly available on the AES home page [2] on April 19, 2000.

NIST performed an analysis of mathematically optimized ANSI C and Java™ implementations^2^ of the candidate algorithms that were provided by the submitters prior to the beginning of Round 1. NIST’s testing of ANSI C implementations focused on the speed of the candidates on various desktop systems, using different combinations of processors, operating systems, and compilers. The submitters’ Java™ code was tested for speed and memory usage on a desktop system. NIST’s testing results for the ANSI C and Java™ code are presented in Refs. [7] and [28], respectively. Additionally, extensive statistical testing was performed by NIST on the candidates, and results are presented in Ref. [88].

### 1.5 The Selection Process

A team of NIST security personnel convened a series of meetings in order to establish the strategy for AES algorithm selection (see Sec. 2). The team then proceeded to evaluate the papers and comments received during the AES development process, compare the results of the numerous studies made of the finalists and finally make the selection of the proposed AES algorithm. There is a consensus by the team that the selected algorithm will provide good security for the foreseeable future, is reasonably efficient and suitable for various platforms and environments, and provides sufficient flexibility to accommodate future requirements.

### 1.6 Organization of this Report

This report is organized as follows. Section 2 provides details on NIST’s approach to making its selection, and discusses some of the more critical issues that were considered prior to evaluating the algorithms. Section 3 presents the various factors and analysis results that were taken into consideration during the algorithms’ evaluation by NIST; this section presents a number of specific case studies. Section 4 summarizes the intellectual property issue. In Section 5, candidate algorithm profiles summarize the salient information that NIST accrued for each finalist, based on the results summarized in Section 3. Section 6 takes the information from the algorithm profiles and draws comparisons and contrasts, in terms of the advantages and disadvantages identified for each algorithm. Finally, Sec. 7 presents NIST’s conclusion for its selection of Rijndael. Section 8 indicates some of the next steps that will occur in the AES development effort.

## 2. Selection Issues and Methodology

### 2.1 Approach to Selection

As the public comment period neared its closing date of May 15, 2000, NIST reconstituted its AES selection team (hereafter called the “team”) that was used for the Round 1 selection of the finalists. This team was comprised of cross-disciplinary NIST security staff. The team reviewed the public comments, drafted this selection report and selected the algorithms to propose as the AES.

A few fundamental decisions confronted the team at the beginning of the selection process. Specifically, the team considered whether to:

- Take a quantitative or qualitative approach to selection;
- Select one or multiple algorithms;
- Select a backup algorithm(s); and
- Consider public proposals to modify the algorithms.

The following sections briefly address these issues.

### 2.2 Quantitative vs Qualitative Review

At one of its first meetings to plan for the post Round 2 activities, the team reviewed the possibility of conducting a quantitative approach as proposed in Ref. [87]. Using this process, each algorithm and combination of algorithms would receive a score based on the evaluation criteria [32]. If such a quantitative approach were feasible, it could provide an explicit assignment of values and allow a comparison of the algorithms. The quantitative approach would also provide explicit weighting of each AES selection factor. However, the consensus of the team was that the degree of subjectivity of many of the criteria would result in numeric figures that would be debatable. Moreover, the issue of quantitative review had been raised by the public at various times during the AES development effort (most recently at AES3), and there seemed to be little agreement regarding how different factors should be weighted and scored. Team members also expressed concern that determining a quantitative scoring system without significant public discussion would give the impression that the system was unfair. For those reasons, the team concluded that a quantitative approach to selection was not workable, and decided to proceed as they did after Round 1. Namely, the team decided to review the algorithms’ security, performance, implementation, and other characteristics, and to make a decision based upon an overall assessment of each algorithm—keeping in mind that security considerations were of foremost concern.

### 2.3 Number of AES Algorithms

During the course of the Round 1 and 2 public evaluation periods, several arguments were made regarding the number of algorithms that should be selected for inclusion in the AES. In addition, the issue was raised about the selection of a “backup” algorithm in the case that a single AES algorithm were selected and later deemed to be unsuitable. This could occur, for example, because of a practical attack on the algorithm or an intellectual property dispute. The team decided that it was necessary to address this issue as early as possible, in part to narrow its scope of options under consideration during the rest of the selection process.

Several arguments made in favor of multiple algorithms (and/or against a single algorithm) included:

- In terms of resiliency, if one AES algorithm were broken, there would be at least one more AES algorithm available and implemented in products. Some commenters expressed the concern that extensive use of a single algorithm would place critical data at risk if that algorithm were shown to be insecure [42] [51] [52].
- Intellectual property (IP) concerns could surface later, calling into question the royalty-free availability of a particular algorithm. An alternative algorithm might provide an immediately available alternative that would not be affected by the envisioned IP concern [52].
- A set of AES algorithms could cover a wider range of desirable traits than a single algorithm. In particular, it might be possible to offer both high security and high efficiency to an extent not possible with a single algorithm [47] [52].

The public also submitted arguments in favor of a single AES algorithm (and/or against multiple algorithms). Some of those arguments suggested that:

- Multiple AES algorithms would increase interoperability complexity and raise costs when multiple algorithms were implemented in products [17] [91].
- Multiple algorithms could be seen as multiplying the number of potential “intellectual property attacks” against implementers [17] [47] [48].
- The specification of multiple algorithms might cause the public to question NIST’s confidence in the security of any of the algorithms [6] [91].
- Hardware implementers could make better use of available resources by improving the performance of a single algorithm than by including multiple algorithms [92].

The team discussed these and other issues raised during Round 2 regarding single or multiple AES algorithms. The team recognized the likelihood, as evidenced by commercial products today, that future products will continue to implement multiple algorithms, as dictated by customer demand, requirements for interoperability with legacy/proprietary systems, and so forth. The Triple data Encryption Standard (Triple DES), which NIST anticipates will remain a FIPS-approved algorithm for the foreseeable future, is expected to be available in many commercial products for some time, as are other FIPS and non-FIPS algorithms. In some regard, therefore, the presence of these multiple algorithms in current products provides a degree of systemic resiliency—as does having multiple AES key sizes. In the event of an attack, NIST would likely assess options at that time, including whether other AES finalists were resistant to such an attack, or whether entirely new approaches were necessary.

With respect to intellectual property issues, vendors noted that if multiple AES algorithms were selected, market forces would likely result in a need to implement all AES algorithms, thus exposing the vendors to additional intellectual property risks.

At the AES3 conference, there was significant discussion regarding the number of algorithms that should be included in the AES. The vast majority of attendees expressed their support—both verbally and with a show of hands—for selecting only a single algorithm. There was some support for selecting a backup algorithm, but there was no agreement as to how that should be accomplished. The above sentiments were reflected in written comments provided to NIST by many of the attendees after the conference.

The team considered all of the comments and factors above before making the decision to propose only a single algorithm for the AES. The team felt that other FIPS-approved algorithms will provide a degree of systemic resiliency, and that a single AES algorithm will promote interoperability and address vendor concerns about intellectual property and implementation costs.

### 2.4 Backup Algorithm

As noted earlier, intertwined in the discussion of multiple AES algorithms was the issue of whether to select a backup algorithm, particularly in the case of a single AES algorithm. A backup could take a number of forms, ranging from an algorithm that would not be required to be implemented in AES validated products (“cold backup”), to requiring the backup algorithm in AES products as a “hot backup.” It was argued by some commenters that, in many respects, a backup algorithm was nearly equivalent to a two-algorithm AES, since many users would reasonably demand that even a “cold backup” be implemented in products.

Given 1) the vendors’ concerns that a backup algorithm would be a *de facto* requirement in products (for immediate availability in the future), 2) the complete uncertainty of knowing the potential applicability of future breakthroughs in cryptanalysis, 3) NIST’s interest in promoting interoperability, and 4) the availability of other algorithms (FIPS and non-FIPS) in commercial products, the team decided not to select a backup algorithm.

As with its other cryptographic algorithm standards, NIST will continue to follow developments in the cryptanalysis of the AES algorithm, and the standard will be formally reevaluated every five years. Maintenance activities for the AES standard will be performed at the appropriate time, in full consideration of the situation’s particular circumstances. If an issue arises that requires more immediate attention, NIST will act expeditiously and consider all available alternatives at that time.

### 2.5 Modifying the Algorithms

During Rounds 1 and 2, NIST received a number of comments that expressed an interest in increasing the number of rounds (or repetitions) of certain steps of the algorithms. Although some comments offered explicit rationale for an increase in the number of rounds (e.g., choosing an algorithm with twice the number of rounds that the currently best known reduced-round analysis requires), many did not. NIST noted that the submitters of the two algorithms that received the most comments regarding an increase in rounds, RC6 and Rijndael, did not choose to increase the number of rounds at the end of Round 1 (when “tweak” proposals were being considered). Additionally, the Rijndael submitters even stated “the number of rounds of Rijndael provides a sufficient margin of security with respect to cryptanalytic attack.” [23]

The following issues and concerns were expressed during the team’s discussions:

- For some algorithms, it is not clear how the algorithm would be fully defined (e.g., the key schedule) with a different number of rounds, or how such a change would impact the security analysis.
- Changing the number of rounds would impact the large amount of performance analysis from Rounds 1 and 2. All performance data for the modified algorithm would need to be either estimated or performed again. In some cases, especially in hardware and in memory-restricted environments, estimating algorithm performance for the new number of rounds would not be a straightforward process.
- There was a lack of agreement in the public comments regarding the number of rounds to be added, and which algorithms should be altered.
- The submitters had confidence in the algorithms as submitted, and there were no post-Round 1 “tweaked” proposals for an increased numbers of rounds.

After much discussion, and given the factors listed above, the team decided that it would be most appropriate to make its recommendation for the AES based on the algorithms as submitted (i.e., without changing the number of rounds).

## 3. Technical Details of the Round 2 Analysis

### 3.1 Notes on Sec. 3

The analyses presented in this paper were performed using the original specifications submitted for the finalists prior to the beginning of Round 2. Most of the analysis of MARS considered the Round 2 version [15], in which modifications had been made to the original submitted specifications [100]. Some of the studies—including the NIST software performance analyses [7] [28]—used algorithm source code that was provided by the submitters themselves.

While NIST does not vouch for any particular data items that were submitted, all data was taken into account. In some cases, the data from one study may not be consistent with that of other studies. This may be due, for example, to different assumptions made for the various studies. NIST considered these differences into account and attempted to determine the general trend of the information provided. For the various case studies presented in Sec. 3, this report summarizes some of these analyses and results, but the reader should consult the appropriate references for more complete details.

### 3.2 General Security

Security was the foremost concern in evaluating the finalists. As stated in the original call for candidates [32], NIST relied on the public security analysis conducted by the cryptographic community. *No attacks have been reported against any of the finalists, and no other properties have been reported that would disqualify any of them.*

The only attacks that have been reported to data are against simplified variants of the algorithms: the number of rounds is reduced or simplified in other ways. A summary of these attacks against reduced-round variants, and the resources of processing, memory, and information that they require, is discussed in Sec. 3.2.1 and presented in Table 1.

It is difficult to assess the significance of the attacks on reduced-round variants of the finalists. On the one hand, reduced-round variants are, in fact, different algorithms, so attacks on them do not necessarily imply anything about the security of the original algorithms. An algorithm could be secure with n rounds even if it were vulnerable with *n*−1 rounds. On the other hand, it is standard practice in modern cryptanalysis to try to build upon attacks on reduced-round variants, and, as observed in Ref. [56], attacks get better over time. From this point of view, it would seem to be prudent to try to estimate a “security margin” of the candidates, based on the attacks on reduced-round variants.

One possible measure of the security margin, based on the proposal in Ref. [10], is the degree to which the full number of rounds of an algorithm exceeds the largest number of rounds that have been attacked. This idea and its limitations are discussed in Sec. 3.2.2. There are a number of reasons not to rely heavily on any single figure of merit for the strength of an algorithm; however, this particular measure of the security margin may provide some utility.

NIST considered other, less quantifiable characteristics of the finalists that might conceivably impact upon their security. Confidence in the security analysis conducted during the specified timeframe of the AES development process is affected by the ancestry of the algorithms and their design paradigms as well as the difficulty of analyzing particular combinations of operations using the current framework of techniques. These issues are discussed in Secs. 3.2.3 and 3.2.4. The statistical testing that NIST conducted on the candidates is discussed in Sec. 3.2.5. Various public comments about the security properties of the finalists are discussed in Sec. 3.2.6. NIST’s overall assessment of the security of the finalists is summarized in Sec. 3.2.7.

#### 3.2.1 Attacks on Reduced-Round Variants

Table 1 summarizes the attacks against reduced-round variants of the finalists. For each attack, the table gives a reference to the original paper in which the attack was described, the number of rounds of the variant under attack, the key size, the type of attack, and the resources that are required. The three resource categories that may be required for the attack are information, memory, and processing.

The “Texts” column indicates the information required to effect the attack, specifically, the number of plaintext blocks and corresponding ciphertext blocks encrypted under the secret key. For most of the attacks, it does not suffice for the adversary to intercept arbitrary texts; the plaintexts must take a particular form of the adversary’s choosing. Such plaintexts are called chosen plaintexts. In the discussions of the attacks in Secs. 3.2.1.1–3.2.1.5, it is noted when an attack can use any known plaintext, as opposed to chosen plaintext.

The “Mem. Bytes” column indicates the largest number of memory bytes that would be used at any point in the course of executing the attack; this is not necessarily equivalent to storing all of the required information.

The “Ops.” column indicates the expected number of operations that are necessary to perform the attack. It is difficult to translate such a number into a time estimate, because the time will depend on the computing power available, as well as the extent to which the procedure can be conducted in parallel. The nature of the operations will also be a factor; they will typically be full encryption operations, but the operations may also be partial encryptions or some other operation. Even full encryptions will vary in the required processing time across algorithms. Therefore, the number of operations required for an attack should be regarded only as an approximate basis for comparison among different attacks. The references should be consulted for full details.

A useful benchmark for the processing that is required for the attacks on reduced-round variants is the processing that is required for an attack by key exhaustion, that is, by trying every key. Any block cipher, in principle, can be attacked in this way. For the three AES key sizes, key exhaustion would require 2^127^, 2^191^, or 2^255^ operations, on average. Even the smallest of these is large enough that any attacks by key exhaustion are impractical today and likely to remain so for at least several decades.

Exhaustive key search requires little memory and information and can be readily conducted in parallel using multiple processors. Thus, any attack that required more operations than are required for the exhaustive key search probably would be more difficult to execute than exhaustive key search. For this reason, many of the attacks on reduced-round variants are only relevant to the larger AES key sizes, although the processing requirements are nevertheless impractical today. Similarly, the memory requirements of many of the reported attacks against reduced-round variants are significant.

Practical considerations are also relevant to the information requirements of the reported attacks against reduced-round variants. Almost all of these attacks require more than 2^30^ encryptions of chosen plaintexts; in other words, more than a billion encryptions, and in some cases far more are required. Even if a single key were used this many times, it might be impractical for an adversary to collect so much information. For instance, there are linear and differential attacks in Ref. [12] and Ref. [64] on DES that require 2^43^ known plaintexts and 2^47^ encryptions of chosen plaintexts. However, NIST knows of no circumstance in which those attacks were carried out against DES.

One model for collecting such large amounts of information would require physical access for an adversary to one or more encryption devices that use the same secret key. In that case, another useful benchmark would be the memory that would be required to store the entire “codebook,” in other words, a table containing the ciphertext blocks corresponding to every possible plaintext block. Such a table would require 2^132^ bytes of memory for storage.

The following are comments on the attacks presented in Table 1.

##### 3.2.1.1 MARS

There are many ways to simplify MARS for the purpose of analysis because of the heterogeneous structure consisting of four different types of rounds. The 16 keyed rounds of the cryptographic core are “wrapped” in 16 unkeyed mixing rounds and pre- and post-whitening.

Four attacks on three simplified variants of MARS were presented in Refs. [57] and [58]. The first variant includes 11 core rounds, without any mixing rounds or whitening. The authors attack this variant with a new type of truncated differential attack, called the boomerang-amplifier, extending the methods in Ref. [90]. The second variant includes both the whitening and the full 16 mixing rounds, while reducing the core rounds from 16 to 5. Two different meet-in-the-middle attacks are proposed on this variant; the adversary does not need to choose the plaintexts for these attacks. The third variant includes the whitening, while reducing both the number of mixing rounds and the number of core rounds from 16 to 6.

Another paper reports an impossible differential for 8 of the 16 rounds of the MARS core [11]. The authors imply that the existence of an impossible differential typically leads to an attack that recovers the secret key from a variant that is a couple of rounds longer than the differential. Because the attack does not actually exist, this attack is not included in Table 1.

##### 3.2.1.2 RC6

The two papers presenting attacks on variants of RC6 both present a small, but iterative, statistical bias in the round function. The resulting statistical correlations between inputs of a certain form and their outputs can be used to distinguish some number of rounds of RC6 from a random permutation. In other words, the two papers construct “distinguishers.” Both papers assume that the distribution of the subkeys is uniformly random; the attack described in Ref. [39] on a 14 round variant of RC6, also assumes that the variable rotation amounts produced within the round function are random. In Ref. [60], the authors describe a distinguisher that they estimate, based on systematic experimental results, will apply to variants of RC6 with up to 15 rounds. Attacks, i.e., methods for recovering the secret key, are described for 12, 14, and 15 round variants. For a class of weak keys, estimated to be one key in 2^80^, the non-randomness is estimated to persist in reduced-round variants employing up to 17 rounds of RC6. In Ref. [76], the RC6 submitters comment on the results in Ref. [60] and observe that those results support their own estimates of the security of RC6.

##### 3.2.1.3 Rijndael

The Rijndael specification describes a truncated differential attack on 4, 5, and 6 round variants of Rijndael [22], based on a 3 round distinguisher of Rijndael. This attack is called the “Square” attack, named after the cipher on which the attack was first mounted. In Ref. [40], truncated differentials are used to construct a different distinguisher on 4 rounds, based on the experimentally confirmed existence of collisions between some partial functions induced by the cipher. This distinguisher leads to a collision attack on 7 round variants of Rijndael.

The other papers that present attacks on variants of Rijndael build directly on the Square attack. In Ref. [63], the Square attack is extended to 7 round variants of Rijndael by guessing an extra round of subkeys. Table 1 indicates the results for the 192 and 256 bit key sizes, where the total number of operations remains below those required for exhaustive search. Similar attacks are described in Ref. [37]. These attacks are improved, however, by a partial summing technique that reduces the number of operations. The partial summing technique is also combined with a technique for trading off operations for information, yielding attacks on 7 and 8 round variants that require almost the entire codebook. The same paper also presents a related key attack on a 9 round variant with 256 bit keys. This attack requires not only encryptions of chosen plaintexts under the secret key, but also encryptions under 255 other keys that are related to the secret key in a manner chosen by the adversary.

##### 3.2.1.4 Serpent

In Ref. [57], the amplified boomerang technique is used to construct a 7 round distinguisher of Serpent, leading to an attack on a variant of Serpent with 8 rounds for the 192 and 256 bit key sizes. In Ref. [58], a refinement based on an experimental observation reduces the texts, memory, and processing required for the attack; an extension to an attack on a 9 round variant is also offered. The same paper also presents a standard meet-in-the-middle attack and differential attacks on 6 and 7 round variants of Serpent, and a standard boomerang attack on an 8 round variant of Serpent that requires the entire codebook.

##### 3.2.1.5 Twofish

The Twofish team has found two attacks on variants of Twofish. In Ref. [35], a 5 round impossible differential is used to attack a 6 round variant of Twofish under 256 bit keys, with the required number of processing operations equivalent to that required for an exhaustive search. If the pre- and post-whitening is removed from the variant, then the attack can be extended to 7 rounds; alternatively, without whitening, 6 round variants can be attacked with a complexity less than that of an exhaustive search for each key size. In Ref. [36], the Twofish team explains why the partial chosen-key and related key attack on a 9 round variant of Twofish that they reported in the Twofish specification does not work. The best such attack of which they are aware applies to a 6 round variant, or a 7 round variant without whitening. The Twofish specification [83] also reports attacks on reduced-round variants of Twofish that are considerably simplified in other ways: for example, by using fixed S-boxes, by removing whitening or subkeys, or by allowing partial key guesses.

Outside of attacks mounted by the Twofish team, NIST knows of no attacks that have been mounted on Twofish by simply reducing the number of rounds. In Ref. [70], differential characteristics on 6 rounds are presented that apply only to certain key dependent S-boxes and thus, only to a fraction of the keys. This particular fraction of the keys could be considered as a class of weak keys, because the authors claim that characteristics like theirs should lead directly to an attack on 7 or eight round variants of Twofish. Because the attack does not actually exist, it does not appear in Table 1. In Ref. [59], an attack is mounted on a 4 round variant of Twofish in which 32 bit words are scaled down to 8 bit words; other properties of Twofish are also explored.

#### 3.2.2 Security Margin

NIST wished to evaluate the likelihood that an analytic shortcut attack would be found for the candidate algorithms with all specified rounds in the next several decades, or before attacks by key exhaustion become practical. It is difficult, however, to extrapolate the data for reduced-round variants to the actual algorithms. The attacks on reduced round variants are generally not even practical at this time because they require huge amounts of resources. In fact, most of these attacks on reduced round variants are, arguably, more difficult to execute in practice than attacks by exhaustive key search, despite smaller processing requirements, because of their information and memory requirements. Moreover, even if a shortcut attack on a simplified variant were practical, the original algorithm might remain secure.

Nevertheless, attacks will improve in the future, and the resources available to carry them out will be greater, so it might be prudent to favor algorithms that appear to have a greater margin for security. If only a little simplification allows an attack on one algorithm, but a second algorithm has only been attacked after much greater simplification, then that may be an indication that the second algorithm has a greater margin for security. Simplification includes round reductions, which is not surprising, because the most notable frameworks of attacks, differential and linear cryptanalysis, may be effectively resisted if the number of rounds is high enough. Therefore, the full number of rounds specified for the algorithm has been compared to the largest number of rounds at which an attack currently exists. In Ref. [85], the ratio of these numbers was defined as the “safety factor” and calculated for each candidate.

There are several problems with relying heavily on this measure, or on any single figure of merit that is based on the attacks on reduced-round variants. In general, the results will be biased against algorithms that attract greater scrutiny in a limited analysis period. This could plausibly occur, for example, if a particular algorithm is simpler, or at least appears to be simpler, to analyze against certain attacks. Another factor could be the ancestry of the algorithm and its constituent techniques, and the existence of previous attacks upon which to build. The proposed measure would tend to favor novel techniques for resisting attacks, techniques that have not yet stood the test of time. Similarly, the proposed measure may not be a good index to the resistance of the algorithms to new and novel techniques for attacking algorithms.

To develop a measure based on the largest number of rounds that are currently attacked is also technically problematic, as is acknowledged in Ref. [85]. There is no natural definition for the number of analyzed rounds, or even the total number of rounds specified for each algorithm. For example, should the whitening in MARS, Twofish, RC6, and Rijndael count as rounds or partial rounds? MARS has 16 unkeyed mixing rounds and 16 keyed core rounds: is MARS a 16 round or a 32 round algorithm, or something in between? Should attacks that ignore the mixing rounds be considered? Should reduced-round variants of Serpent or Rijndael be required to inherit the slightly modified final round? Another complicating factor is the key size, especially for Rijndael, which varies the number of rounds depending on the key size.

What types of attacks should be included in the definition? Some attacks were successful against only a small fraction of keys; some required encryption operations under related unknown keys; some distinguished outputs from random permutations without an explicit method for recovering the key; and some relied on experimental conjectures. In addition, the attacks required considerably different resources; some even assume that nearly the entire codebook was available to the attacker.

In light of these difficulties, NIST did not attempt to reduce its assessment of the security margins of the finalists to a single measurement. NIST considered all of the reported data, and used the raw number of analyzed rounds out of the total rounds specified for an algorithm as a first approximation. The results are summarized below for each finalist.

Note that the rounds defined for the candidates are not necessarily comparable to each other. For example, the algorithms based on the Feistel construction, MARS, RC6, and Twofish, require two rounds to alter an entire word of data, while a single round of Rijndael or Serpent accomplishes this.

MARS: The results for MARS depend on the treatment of the “wrapper,” i.e., the pre- and post-whitening and the 16 unkeyed mixing rounds that surround the 16 keyed core rounds. Without the wrapper, 11 out of the 16 core rounds have been attacked. With the wrapper, MARS has many more rounds than have been successfully attacked: only 5 out of the 16 core rounds, or 21 out of the 32 total rounds have been attacked. Or, if the wrapper is regarded as a pair of single, keyed pair of single, keyed rounds, then 7 out of the 18 rounds have been attacked. For any of these cases, MARS appears to offer a high security margin.

RC6: Attacks have been mounted against 12, 14, and 15 out of the 20 rounds of RC6, depending on the key size. The submitters point out in Ref. [78] that these results support their original estimate that as many as 16 out of the 20 rounds may be vulnerable to attack. RC6 appears to offer an adequate security margin.

Rijndael: For 128 bit keys, 6 or 7 out of the 10 rounds of Rijndael have been attacked, the attack on 7 rounds requiring nearly the entire codebook. For 192 bit keys, 7 out of the 12 rounds have been attacked. For 256 bit keys, 7, 8, or 9 out of the 14 rounds have been attacked. The 8 round attack requires nearly the entire codebook, and the 9 round attack requires encryptions under related unknown keys. The submitters point out in Ref. [26] that the incremental round improvements over their own 6 round Square attack come at a heavy cost in resources. Rijndael appears to offer an adequate security margin.

Serpent: Attacks have been mounted on 6, 8, or 9 out of 32 rounds of Serpent, depending on the key size. Serpent appears to offer a high security margin.

Twofish: The Twofish team has mounted an attack on 6 out of the 16 rounds of Twofish that requires encryption operations under related unknown keys. Another attack proposed on 6 rounds for the 256 bit key size is no more efficient than exhaustive key search. Twofish appears to offer a high security margin.

#### 3.2.3 Design Paradigms and Ancestry

The history of the underlying design paradigms affects the confidence that may be placed in the security analysis of the algorithms. This also applies to the constituent elements of the design, such as the S-boxes. It may require more time for attacks to be developed against novel techniques, and traditional techniques may tend to attract more analysis, especially if attacks already exist on which to build. For example, the Feistel construction, such as employed by DES, has been well studied, and three of the finalists use variations of this structure. Another element that can affect public confidence is the design of the S-boxes, which can be suspected of containing a hidden “trapdoor” that can facilitate an attack. These considerations are discussed below for each finalist.

MARS: The heterogeneous round structure of MARS appears to be novel. Both the mixing round and the core rounds are based on the Feistel construction, with considerable variation. MARS uses many different operations, most of which are traditional. A product of key material and data is used to regulate the variable rotation operation. The S-box was generated deterministically to achieve certain desired properties; thus, the MARS specification asserts that MARS is unlikely to contain any structure that could be used as a trapdoor for an attack. The MARS specification does not cite any algorithm as an ancestor.

RC6: The design of RC6 evolved from the design of RC5, which has undergone several years of analysis. The security of both algorithms relies on variable rotations as the principal source of non-linearity; there are no S-boxes. The variable rotation operation in RC6, unlike RC5, is regulated by a quadratic function of the data. The key schedules of RC5 and RC6 are identical. The round structure of RC6 is a variation on the Feistel construction. The RC6 specification asserts that there are no trapdoors in RC6 because the only *a priori* defined part of RC6 is the well known mathematical constants used during key setup.

Rijndael: Rijndael is a byte-oriented cipher based on the design of Square. The submitters’ presentation of the Square attack served as a starting point for further analysis. The types of substitution and permutation operations used in Rijndael are standard. The S-box has a mathematical structure, based on the combination of inversion over a Galois field and an affine transformation. Although this mathematical structure might conceivably aid an attack, the structure is not hidden as would be the case for a trapdoor. The Rijndael specification asserts that if the S-box was suspected of containing a trapdoor, then the S-box could be replaced.

Serpent: Serpent is a byte-oriented algorithm. The types of substitution and permutation operations are standard. The S-boxes are generated deterministically from those of DES to have certain properties; the Serpent specification states that such a construction counters the fear of trapdoors. The Serpent specification does not cite any algorithm as an ancestor.

Twofish: Twofish uses a slight modification of the Feistel structure. The Twofish specification does not cite any particular algorithm as its ancestor, but it does cite several algorithms that share an important feature of Twofish, the key-dependent S-boxes, and weighs the various design approaches to them. The Twofish specification asserts that Twofish has no trapdoors and supports this conclusion with several arguments, including the variability of the S-boxes.

#### 3.2.4 Simplicity

Simplicity is a property whose impact on security is difficult to assess. On the one hand, complicated algorithms can be considered more difficult to attack. On the other hand, results may be easier to obtain on a simple algorithm, and an algorithm that is perceived to be simple may attract relatively more scrutiny. Therefore, during the AES analysis period, it may have been easier to be confident in the analysis of a simple algorithm.

There is no consensus, however, on what constitutes simplicity. MARS has been characterized as complicated in several public comments, but the submitters point out in Ref. [20] that MARS requires fewer lines of C code in the Gladman implementations than Rijndael, Twofish, and Serpent. RC6, by contrast, is generally regarded as the simplest of the finalists, yet the modular multiplication operation it contains is arguably much more complicated than typical cipher operations. In Ref. [49], the MARS team points out that the published linear analysis of RC5 was found to be in error three years after the publication of that analysis, so seemingly simple ciphers are not necessarily easier to analyze.

For standard differential cryptanalysis, the type of operations employed tangibly affects the rigor of the security analysis. If key material is mixed with data only by the XOR operation, as in Serpent and Rijndael, then plaintext pairs with a given XOR difference are the natural inputs, and the security analysis is relatively clean. If key material is mixed with data by more than one operation, as in the other finalists, then there is no natural notion of difference, and the security analysis requires more estimates. Similarly, the use of variable rotations in MARS and RC6 would seem to inhibit the possibility of clean security results against a variety of differential and linear attacks.

Another aspect of simplicity that relates to security analysis is scalability. If a simplified variant can be constructed with a smaller block size, for example, then conducting experiments on the variant becomes more feasible, which in turn may shed light on the properties of the original algorithm. In Ref. [79], it is claimed that the lack of smaller versions of MARS severely hampers analysis and experimentation. Similarly, in Ref. [59], the authors assert that a “realistic” scaled-down variant of Twofish seems difficult to construct. Both claims are plausible, although it should be noted that the MARS and Twofish specifications contain considerable analysis of their individual design elements. The Serpent specification asserts, plausibly, that it would not be difficult to construct scaled-down variants of Serpent. RC6 and Rijndael are scaleable by design.

#### 3.2.5 Statistical Testing

NIST conducted statistical tests on the AES finalists for randomness by evaluating whether the outputs of the algorithms under certain test conditions exhibited properties that would be expected of randomly generated outputs. These tests were conducted for each of the three key sizes. In addition, NIST conducted a subset of the tests on reduced-round versions of each algorithm. All of the testing was based on the NIST Statistical Test Suite [80].

For the full round testing, each of the algorithms produced random-looking outputs for each of the key sizes. For the reduced-round testing of each finalist, the outputs of an early round appear to be random, as do the outputs of each subsequent round. Specifically, the output of MARS appears to be random at four or more core rounds, RC6 and Serpent at four or more rounds, Rijndael at three or more rounds, and Twofish at two or more rounds. The test conditions and results are described in Ref. [88]. For comments on the limitations of NIST’s methodology, see Ref. [69].

Additional testing, as described in Ref. [53] and limited to RC6, confirmed NIST’s results for RC6 on certain statistical tests. Reference [74] presented detailed results from measuring the diffusion properties of full round and reduced round versions of the finalists. The quantities measured—including the degrees of completeness, of the avalanche effect, and of strict avalanche criterion—were “indistinguishable from random permutations after a very small number of rounds,” for all of the finalists.

In summary, none of the finalists was statistically distinguishable from a random function.

#### 3.2.6 Other Security Observations

Many observations have been offered about various properties that might impact the security of the finalists. Because the implications of these observations are generally subjective, they did not play a significant role in NIST’s selection.

MARS: In Ref. [20], the MARS team conjectures that the heterogeneous structure of MARS and its variety of operations constitute a kind of insurance against the unknown attacks of the future. The MARS key schedule requires several stages of mixing; in Ref. [77], key schedules that require the thorough mixing of key bits are cited for security advantages. The estimates in the MARS specification of the resistance of the core to linear cryptanalysis are questioned in Ref. [79]. In Ref. [61], one conjectured estimate from the MARS specification is proven incorrect. In Ref. [14], it is pointed out that the MARS S-box does not have all of the properties that the designers required. No attacks are proposed based on these observations. In Ref. [49], the MARS team offers a clarification of its analysis, supporting the original assessment that MARS is resilient against linear attacks.

RC6: In Ref. [77], the thorough mixing provided by the RC6 key schedule is cited as a security advantage. In Ref. [20], the concern is raised that RC6 relies mainly on data-dependent rotations for its security, constituting a “ ‘single point of failure’ … (as it does not use S-boxes).”

Rijndael: In Ref. [86], the author discusses three concerns about the mathematical structure of Rijndael and the potential vulnerabilities that result. First, he observes that all of the operations of the cipher act on entire bytes of the data, rather than bits; this property allows the Square attack on reduced-round variants. Moreover, the nearly symmetric movement of the bytes troubles him. The only break to the symmetry is the use of different round constants in the key schedule, and for the first eight rounds, these constants are only one bit. If Rijndael were simplified to omit these round constants, then encryption would be compatible with rotating each word of the data and subkeys by a byte.

The second concern discussed in Ref. [86] is that “Rijndael is mostly linear.” He disagrees with the deliberate design decision to avoid mixing the XOR operations with ordinary addition operations. He illustrates how to apply a linear map to the bits within each byte without changing the overall algorithm, by compensating for the linear map in the other elements of the cipher, including the key schedule. Similarly, the Galois field that underlies the S-box can be represented in different basis vectors or can be transformed to other Galois fields with different defining polynomials. In other words, the Rijndael’s mathematical structure permits many equivalent formulations. The author suggests that, by performing a series of manipulations to the S-box, an attacker might be able to find a formulation of Rijndael with an exploitable weakness.

The third concern discussed in Ref. [86] is the relatively simple algebraic formula for the S-box, which is given in the Rijndael specification. The formula is a polynomial of degree 254 over the given Galois field, but there are only nine terms in the polynomial, far fewer than would be expected in a typical randomly generated S-box of the same size. The mathematical expression for the iteration of several rounds of Rijndael would be much more complex, but the author asserts that the growth of the expression size as a function of rounds has not been analyzed in detail. He presents some examples of calculations in this setting, including the possible use of a “normal” basis, under which the squaring operation amounts to just a rotation of bits. If the expression for five rounds of Rijndael turned out to contain, say, only a million terms, then the author asserts that a meet in the middle attack could be mounted by solving a large system of linear equations. Such an attack would require the attacker to collect two million plaintext-ciphertext pairs.

In Ref. [86], it is also noted that an attacker that recovers or guesses appropriate bits of Rijndael’s subkeys will be able to compute additional bits of the subkeys. (In the case of DES, this property aided the construction of linear and differential attacks.) Extensions of this observation are discussed in Ref. [37]; its authors deem these properties worrisome and suggest that, contrary to a statement in the Rijndael specification, the key schedule does not have high diffusion.

In Ref. [72], some properties of the linear part of the round function in Rijndael are explored. In particular, the linear mapping within the round function has the property that 16 iterations are equivalent to the identity mapping. The authors suggest that this casts doubt on the claim in the Rijndael submission that the linear mapping provides high diffusion over multiple rounds. In Ref. [24], the Rijndael submitters explain that the observations in Ref. [72] do not contradict their claims about the security of Rijndael. The authors of Ref. [72] offer a further response in Ref. [71].

Serpent: In Ref. [3], the Serpent team asserts that Serpent is the most secure of the finalists. They cite Serpent’s many extra rounds, beyond those needed to resist today’s attacks, as a reason why future advances in cryptanalysis should not break its design. In Ref. [67], a concern is raised about the small size of Serpent’s S-boxes. Although the author views the S-boxes as well designed with respect to linear and differential cryptanalysis, the S-boxes may turn out to exhibit some other properties that are exploitable in an attack. No such properties or attacks have been proposed. In Ref. [86], it is noted that an attacker that recovers or guesses appropriate bits of the subkeys will be able to compute additional bits of the subkeys.

Twofish: Twofish uses an innovative paradigm, in the form of key-dependent S-boxes. This creates an unusual dependency between the security of the algorithm and the structure of the key schedule and S-boxes. In the 128 bit key case (where there are 128 bits of entropy), Twofish may be viewed as a collection of 2^64^ different cryptosystems. A 64 bit quantity (representing 64 bits of the original 128 bits of entropy) that is derived from the original key controls the selection of the cryptosystem. For any particular cryptosystem, 64 bits of entropy remain, in effect, for the key. As a result of this partitioning of the 128 bits of entropy derived from the original key, there has been some speculation [66] that Twofish may be amenable to a divide-and-conquer attack. In such an attack, an attacker would determine which of the 2^64^ cryptosystems is in use, and then determine the key to the cryptosystem. If a method could be devised to execute these steps, the work factor for each step would presumably be 2^64^. However, no general attack along this line has been forthcoming. That is, if an attacker is faced with the task of decrypting ciphertext encrypted with a 128 bit key, it is not clear that the partitioning of the 128 bits of entropy gives the attacker any advantage. On the other hand, if a fixed 128 bit key is used repeatedly, each usage may leak some information about the cryptosystem selected. If an attacker can make repeated observations of the cryptosystem in action, he might conceivably be able to determine which of the 2^64^ cryptosystems is in use. Similar remarks apply to higher key sizes (in general, for *k* bit keys, the cryptosystem is determined by *k*/2 bits of entropy).

This feature of Twofish, called the key separation property of Twofish in Ref. [66], is discussed further in Refs. [55], [68], and [96]. In particular, Ref. [55] notes that the dependence of the S-boxes in Twofish on only 64 bits of entropy in the 128 bit key case was a deliberate design decision. This decision is somewhat analogous to the security/efficiency tradeoff involved in establishing the number of rounds in a system with a fixed round function. The authors note that if the S-boxes had depended on 128 bits of entropy, the number of rounds of Twofish would have had to be reduced in order to avoid an overly negative effect on key agility and/or throughput.

In Ref. [55], the Twofish team asserts that key-dependent S-boxes constitute a form of security margin against unknown attacks.

In Ref. [59], the author explores a variety of properties of Twofish, including the construction of truncated differentials for up to 16 rounds. Although these differentials do not necessarily lead to an attack, the author finds it surprising that non-trivial information can be pushed through all 16 rounds of Twofish.

#### 3.2.7 Summary of Security Characteristics of the Finalists

As noted earlier, no general attacks against any of the finalists is known. Hence, the determination of the level of security provided by the finalists is largely guesswork, as in the case of any unbroken cryptosystem. The following is a summary of the known security characteristics of the finalists.

MARS appears to have a high security margin. A precise characterization of MARS is difficult because of the fact that MARS employs two different kinds of rounds. MARS has received some criticism based on its complexity, which may have hindered its security analysis during the timeframe of the AES development process.

RC6 appears to have an adequate security margin. However, RC6 has received some criticism because of its low security margin relative to that offered by other finalists. On the other hand, RC6 has been praised for its simplicity, which may have facilitated its security analysis during the specified timeframe of the AES development process. RC6 is descended from RC5, which has received prior scrutiny.

Rijndael appears to have an adequate security margin. The security margin is a bit difficult to measure because the number of rounds changes with the key size. Rijndael has received some criticism on two grounds: that its security margin is on the low side among the finalists, and that its mathematical structure may lead to attacks. However, its structure is fairly simple, which may have facilitated its security analysis during the specified timeframe of the AES development process.

Serpent appears to have a high security margin. Serpent also has a simple structure, which may have facilitated its security analysis during the specified timeframe of the AES development process.

Twofish appears to have a high security margin. Since Twofish uses key-dependent round function, the notion of security margin may have less meaning for this algorithm than for the other finalists. The dependence of the Twofish S-boxes on only *k*/2 bits of entropy in the *k* bit key case has led to a speculation that Twofish may be amenable to a divide-and-conquer attack, although no such attack has been found. Twofish has received some criticism for its complexity, making analysis difficult during the timeframe of the AES development process.

### 3.3 Software Implementations

Software implementations cover a wide range. In some cases, space is essentially unrestricted; in other cases, RAM and/or ROM may be severely restricted. In some cases, large quantities of data are encrypted or decrypted with a single key. In other cases, the key changes frequently, perhaps with each block of data.

Encryption or decryption speed may be traded off against security, indirectly or directly. That is, the number of rounds specified for an algorithm is a factor in security; encryption or decryption speed is roughly proportional to the number of rounds. Thus, speed cannot be studied independently of security, as noted in Sec. 3.3.6.

There are many other aspects of software implementations. Some of these are explored below, along with the basic speed and cost considerations.

#### 3.3.1 Machine Word Size

One issue that arises in software implementations is the basic underlying architectures. The platforms on which NIST performed testing were oriented to 32 bit architectures. However, performance on 8 bit and 64 bit machines is also important, as was recognized in the public comments and analyses. It is difficult to project how various architectures will be distributed over the next 30 years (roughly the minimum period in which the AES is expected to remain viable). Hence, it is difficult to assign weights to the corresponding performance figures that accurately represent their importance during this timeframe. Nonetheless, from the information received by NIST, the following picture emerges:

It appears that over the next 30 years, 8 bit, 32 bit, and 64 bit architectures will all play a significant role (128 bit architectures might be added to the list at some point). Although the 8 bit architectures used in certain applications will gradually be supplanted by 32 bit versions, 8 bit architectures are not likely to disappear. Meanwhile, some 32 bit architectures will be supplanted by 64 bit versions at the high-end, but 32 bit architectures will become increasingly relevant in low-end applications, so that their overall significance will remain high. Meanwhile, 64 bit architectures will grow in importance. Since none of these predictions can be quantified, it appears that versatility is of the essence. That is, an AES should exhibit good performance across a variety of architectures.

Some information on the performance of the finalists with respect to word size may be accrued from Tables 16 through 25 of Appendix A. In this appendix, encryption speeds are grouped into four categories: 8 bit, 32 bit C and assembler code, 64 bit C and assembler code, and other (Java, DSPs, etc.). Graphs are also provided in order to aid the visualization of the table information.

It should be noted that performance cannot be classified by word size alone. One additional factor is the support provided by software. This is noted (but not systematically explored) in the next section.

#### 3.3.2 Other Architectural Issues

Both MARS and RC6 use 32 bit multiplies and 32 bit variable rotations. These operations, particularly the rotations, are not supported on some 32 bit processors. The 32 bit multiply and rotation operations are both awkward to implement on processors of other word sizes. Moreover, some compilers do not actually use the rotation operations even when they are available in the processor instruction set. Therefore, the relative performance of MARS and RC6, when running the same source code, shows somewhat more variance from specific platform (processor and compiler) to platform, than do the other three finalists.

#### 3.3.3 Software Implementation Languages

The performance of the finalists also depends somewhat on the particular high-level language used (e.g., assembler, compiler or interpreter). In some cases, the role played by particular software has a strong effect on performance figures. There is a spectrum of possibilities. At one extreme, hand-coded assembly code will generally produce better performance than even an optimizing compiler. At the other extreme, interpreted languages are, in general, poorly adapted to the task of optimizing performance. Compilers are typically in between. In addition, as noted in the Sec. 3.3.2, some compilers do a better job than others in making use of the support provided by the underlying architecture for operations such as 32 bit rotations. This increases the difficulty of measuring performance across a variety of platforms. Some finalists benefited from the use of certain compilers on certain processors. However, this type of performance increase on specific platforms does not necessarily translate into high performance results across platforms.

There is no clear consensus on the relative importance of different languages. In Ref. [84], the opinion is expressed that assembler coding is the best means of evaluating performance on a given architecture. The reason provided is that hand-coded assembler will be used when speed is important and a hardware implementation is not available. On the other hand, the use of assembler or another means of optimizing for speed may raise costs. Code development cost may be significant, especially if the goal is maximum speed. For example, optimizations may be effected using hand coding for high-level languages such as C, or by the use of assembly code. This developmental cost may or may not translate into significant monetary cost, depending upon the specific environment. In some environments, the speed at which the code runs is perceived as a paramount consideration in evaluating efficiency, overriding cost considerations. In other cases, the time and/or cost of code development is a more important consideration. In some cases, the speed of key setup is more significant than encryption or decryption speed. This makes it difficult to develop a universal metric for evaluating the performance of the finalists.

Code development cost may need to be traded off against speed. That is, the use of standard reference code may minimize cost, but may not allow significant optimization in a particular environment. On the other hand, the use of non-standard code, such as hand-coded assembler, may optimize speed at the expense of higher development cost.

Optimization spans a broad range. Some optimizations may be made without great effort. Furthermore, some optimizations may be portable across platforms. At the opposite extreme, some optimizations require much effort and/or are restricted to particular platforms. Two related examples are discussed in Refs. [43] and [73], in which optimized implementations of the Serpent S-boxes are obtained. This work involves exhaustive searching through possible instruction sequences. The results improve Serpent’s performance on the targeted platforms. However, this level of optimization involves resource expenditures (e.g., 1000 hours of execution of search programs [43]) far beyond optimizations that may be obtained using hand coding. Optimizations obtained by such searches do not necessarily port to different platforms. Maximal optimization on specific platforms may raise the cost of code development substantially.

In Tables 16–21 of Appendix A, the results were obtained via a mixture of reference code and hand-coded assembler. Some finalists (notably, Rijndael and Twofish) performed better on some platforms when hand-coded assembler was used as opposed to compilers. The results from Refs. [43] and [73] and from other papers dealing with heavily optimized implementations of one finalist have been omitted from these tables. Although such papers would be valuable aids in implementing a finalist in practice, their significance for comparing the finalists is questionable since the papers only address a single algorithm. Without knowing the level of effort applied to optimizing the algorithms in the separate studies, it is impractical to compare studies where a single algorithm was optimized. Choosing an AES algorithm on the basis of heavily optimized implementations would not necessarily be an accurate predictor of the general performance of the algorithm in the field, since extreme optimization may not be feasible or cost-effective in many applications.

#### 3.3.4 Variation of Speed With Key Size

The software performance of MARS, RC6, and Serpent does not vary significantly for the three different AES key sizes. For Rijndael and Twofish, however, key setup or encryption/decryption is noticeably slower for 192 bit keys than for 128 bit keys, and slower still for 256 bit keys; see Tables 16–30 in Appendix A.

Rijndael specifies more rounds for the larger key sizes, affecting the speed of both encryption/decryption and key setup. The key setup time remains the fastest among the finalists for the larger key sizes.

For the larger key sizes, Twofish specifies extra layers both in the generation of its subkeys and in the construction of its key-dependent S-boxes. Subkey computation only affects the speed of key setup. However, the S-box construction can affect the speed of either key setup or encryption/decryption, or both, depending on the extent to which the S-boxes are precomputed during key setup. For example, under the full keying option, which optimizes for throughput, the effect of the extra layers in the S-boxes is confined to key setup.

The variation of Rijndael and Twofish for the three key sizes complicates the evaluation and comparison of the performance of all of the finalists. Most of the submitted data applied to the 128 bit case. Rijndael and Twofish pay a performance penalty for the larger key sizes, although in those cases, they arguably offer some compensation in increased security.

#### 3.3.5 Summary of Speed on General Software Platforms

An enormous amount of information has been gathered on the speed of the finalists on a variety of software platforms. These platforms include 32 bit processors (C and Java implementations), 64 bit processors (C and assembler), 8 bit processors (C and assembler), 32 bit smart cards (ARM), and Digital Signal Processors. Tables 16–30 of Appendix A provide the performance findings of the finalists on these software platforms. These tables provide information from various contributors on the encryption speed (Tables 16–21), decryption speed (Tables 22–25), and key setup time (Tables 26–30). Tables 2–4 summarize the performance of the finalists on the various platforms when using 128-keys. Additionally, an overall performance table is also included. The performance of the finalists is grouped into tiers. At times, these tiers were difficult to determine because of the multitude of studies. Hence, the groupings are not meant to be definitive. Tier I is the highest level of performance; Tier III is the lowest level of performance.

In the following assessments, “low-end,” “average” and “high-end” are relative terms used only within the context of these five finalists.

MARS provides average performance for encryption, decryption and key setup.

RC6 provides average to high-end performance for encryption and decryption, and average performance for key setup.

Rijndael provides consistently high-end performance for encryption, decryption and key setup, although performance decreases for the 192 bit and 256 bit key sizes.

Serpent provides consistently low-end performance for encryption and decryption, and platform-dependent performance for key setup.

Twofish provides platform-dependent performance for encryption and decryption, and consistently low-end performance for key setup. The “Full Keying” option was used in the implementations (see Sec. 3.9.2). This option provides the fastest possible encryption time by placing more computations in key setup. Encryption/decryption or key setup performance decreases with the larger key sizes, depending upon the keying option used.

#### 3.3.6 Variation of Speed With Mode

Another factor that may impact an algorithm’s speed is the mode of operation in use. An algorithm running in a non-feedback mode (e.g., Electronic Codebook (ECB) and Counter modes) can be implemented to process data blocks independently and, therefore, simultaneously. The results from the simultaneous processing are then interleaved to produce a stream of information that would be identical to the stream produced by sequential processing. An implementation using this approach is considered to be using an “interleaved mode.” This contrasts with feedback modes of operation (e.g., Cipher Feedback, Cipher Block Chaining, etc.), which must process data blocks sequentially. Thus, interleaved modes have the potential for taking advantage of parallel processing functionality within some processors.

Only a few studies provided data comparing algorithm speed in both feedback and non-feedback modes. In Ref. [19], a TriMedia VLIW CPU was used to test the throughput of the finalists in the ECB mode using varying amounts of interleaving. Although all five algorithms exhibited better speed with interleaving, RC6 benefited the most, in terms of both raw speed and percentage improvement. Although Rijndael’s raw speed remained competitive, the author indicates that Rijndael’s speed varied the least between interleaved and non-interleaved modes [19].

The authors of Ref. [95] offer speed estimates for the finalists on the Alpha 21264 processor for processing single and multiple streams of data (used in feedback and interleaved modes of operation, respectively). The Alpha 21264 processor, like the TriMedia used in Ref. [19], indicates that the speed of RC6 can benefit greatly (by more than 33 % in an interleaved mode, while MARS, Rijndael, and Twofish may not improve at all. Serpent’s speed is estimated to improve somewhat when processing multiple streams simultaneously.

In Ref. [97], all five finalists are implemented in the TMS320C6201 DSP, in both feedback and interleaved modes. As in the previous two studies, Rijndael showed no speed improvement when executed in an interleaved mode. The authors indicate that the DSP tools can very efficiently optimize the Rijndael code (in both mode types), which is the reason for no change in speed [97]. However, all four of the other finalists demonstrated improvements of between 10 % and 40 % when using an interleaved mode for encryption. Twofish experienced the greatest percentage improvement for encryption and decryption, while MARS and RC6 also executed significantly more quickly. Serpent’s improvement was less dramatic.

Irrespective of the raw speed figures, the three studies indicate that for some processors, there may be a significant difference in an algorithm’s speed for encryption and decryption when comparing interleaved and feedback modes. Whether an algorithm demonstrates any improved speed from one mode to another appears to depend on the processor in use. For the processors in question, RC6 consistently improved its speed significantly when used in an interleaved mode, while Rijndael’s speed varied the least between the two mode types.

### 3.4 Restricted-Space Environments

In some environments, relatively small amounts of RAM and/or ROM are available for such purposes as code storage (generally in ROM), representation of data objects such as S-boxes (which could be stored in ROM or RAM, depending on whether pre-computation or Boolean representation is used), and subkey storage (in RAM). In theory, intermediate forms of storage such as EEPROM could be used for non-static quantities such as subkeys. However, this would be impractical in many instances, since it would negatively impact key agility. Thus, in particular, it must be assumed that subkeys are stored in RAM.

Another consideration is that the available RAM must be used for various purposes, such as the storage for intermediate variables during program execution. Thus, it cannot be assumed that a large portion of this space is available for subkey storage.

In restricted-memory environments, the amounts of ROM and RAM needed to implement the finalists may be a factor in determining their suitability for a given environment. A major advantage (and in some cases, a virtual prerequisite) is support for on-the-fly subkey computation (or some equivalent scheme that obviates the necessity of computing and storing all subkeys in advance), as discussed in Sec. 3.8. In addition, ROM usage must be reasonable.

#### 3.4.1 A Case Study

In Ref. [82], the finalists are implemented on a high-end smart card. The smart card is equipped with a Z80 microprocessor (8 bits), a coprocessor, 48 kB of ROM, and 1 kB of RAM. The Z80 can execute logical instructions, 1 bit shifts or rotations, addition, and subtraction. The coprocessor is useful in handling modular multiplications, completing a multiplication within the execution time of a Z80 instruction. The coprocessor can also be called upon for other arithmetic or logical operations, if advantageous.

Code was written so that an encryption or decryption operation runs in the same amount of time, regardless of the specific key or data bits used. This was intended to deflect timing attacks and simple power analysis (Sec. 3.6.1). Defense against differential power analysis and other variants was not considered. On-the-fly subkey computation was used wherever possible. Only the results for encryption and key scheduling are reported, although decryption is also analyzed.

##### 3.4.1.1 Notes on the Finalists

MARS: MARS caused some problems because of its heterogeneous round structure (four different round types). The 2 kB of ROM needed for S-boxes were not a problem because of the plentiful amount of ROM that was available.

Parsing for weak keys caused some problems in restricted-resource environments. It was necessary to use some form of pattern matching in order to eliminate certain patterns arising in key words (10 consecutive zeroes or ones are disallowed). The needed checks increased the execution time and the amount of ROM required. If subkeys needed to be regenerated, processing time was affected. This conditional regeneration of subkeys opens the process to timing attacks. For simplicity, the authors omitted the required weakness checks in the key schedule. Thus, their implementation of MARS was incomplete.

Variable rotations could have caused problems per se because of the constant-time requirement. However, the coprocessor solved this problem by emulating variable rotations using modular multiplications (to left-rotate an *m* bit string by *n* positions, store it twice in a 2*m* bit area, multiply by 2*^n^* mod 2^2*m*^, then extract the high-end *m* bits).

The overall conclusion is that MARS causes problems in restricted-resource environments, including high-end smart cards.

RC6: Encryption in RC6 is well suited to the particular smart card used in this study. This is due to its reliance upon arithmetic operations, which the Z80 can off-load to the coprocessor. As in the case of MARS, variable rotations can be handled using modular multiplications.

The key schedule is simple, but on-the-fly subkey computation is not an option. This could cause problems on low-end cards. In this study, storage for subkeys was plentiful. On the other hand, key setup was very time-consuming, and took about four times as many cycles as encryption.

Rijndael: Rijndael is the most efficient of the finalists according to this study. The AddRoundKey operation was carried out on the coprocessor. Other operations were carried out by the Z80. Key setup was very efficient. However, the efficiency edge of Rijndael over the other finalists was reduced if encryption and decryption were implemented simultaneously, due to the relative lack of resource sharing between encryption and decryption. Specifically, Rijndael would then require twice the amount of ROM reported in the study.

Serpent: Two different Serpent implementation modes are possible: ordinary and bit-sliced. Only the ordinary implementation was employed in this study. 2kB of ROM were required for tables, not a problem on the card employed. Most of the rotations required by Serpent were simpler using the 1 bit rotations of the Z80 rather than the coprocessor. An exception was the 11 bit rotations that were off-loaded to the coprocessor, along with XORs.

It is possible to implement Serpent using 80 bytes of RAM, due to support for on-the-fly subkey computation. However, the authors chose to involve the coprocessor, which required twice as much RAM. Key setup took about twice as many cycles as encryption.

The authors estimate that a bitsliced implementation would degrade speed somewhat, but also reduce ROM requirements.

Twofish: There are several possible modes for implementing Twofish; these are particularly relevant to restricted storage environments such as were used for this study. Rotations were implemented with the Z80’s 1 bit rotations. Subkey additions and XORs were off-loaded to the coprocessor. Performance depended, to some extent, on the size of pre-computed tables; this was not systematically explored.

The connection between decryption and key setup was ignored in all case studies within this study, due to the concentration on encryption. If encryption and decryption were implemented simultaneously, however, Twofish would have a substantial advantage due to its independent subkey computation property.

##### 3.4.1.2 Comparison of the Finalists

The results of this study do not necessarily correspond to other studies, because of the particular features of the smart card used. In particular, the invocation of the coprocessor had a considerable effect on both time and RAM usage.

The results are summarized in Table 5, taken directly from Table 8 of Ref. [82]. In this study, the algorithm implementations were optimized for speed. Note that the MARS implementation was incomplete due to the omission of the weakness check during key setup. This table clearly indicates that Rijndael is superior in every respect, within the scope of the present study. Twofish is next best, followed by RC6; MARS and Serpent seem to fall behind the other three algorithms, depending on the weights assigned to the various categories. The figures listed for MARS are too low, because of the incomplete implementation of the key schedule.

If the parameters of the study were altered, the results would undoubtedly differ somewhat. For example, if encryption and decryption were implemented simultaneously, Serpent and Rijndael would be penalized because of the lack of resource sharing, and Twofish would be rewarded for its independent subkey computation. The presence of an arithmetic coprocessor clearly benefited MARS and RC6, since multiplication and variable rotation could be executed efficiently.

#### 3.4.2 A Second Case Study

In Ref. [54], the performances of four of the finalists (all except Twofish) were simulated on the Motorola 6805 and are summarized in Table 6. “In general, the algorithms were implemented to fit within 120 bytes of RAM including the key schedule. The algorithms were implemented to take about 1024 bytes of ROM, but flexibility was allowed where this would cause a large speed penalty” [54]. The performance for Twofish on the 6805 is quoted from the Twofish submission. The 6805 family contains a number of variants, with RAM ranging from 64 B to 384 B, and ROM ranging from 1240 B to 32 040 B. The author considered two implementations of RC6 and MARS. The first, denoted simply by RC6 and MARS in the table, were designed so that the RAM required for encryption plus subkey storage did not exceed 120 B. The alternative implementations, denoted by RC6(2) and MARS(2) in the table, had no limit on RAM for encryption plus subkey storage. The results are given for the encryption of a single block with a 128 bit key, except that the author considered decryption as well as encryption for Rijndael (RIJN(d)). For Serpent, a bitslice implementation was used to conserve ROM.

It is important to note that this study only tested the Round 1 version of MARS, which had a different key schedule than the Round 2 version. Before Round 2, the MARS submission team “tweaked” the key schedule, with a goal of making the key schedule better suited for restricted-space environments. Unfortunately, during Round 2, there was a general lack of cross cutting performance testing in those environments. Therefore, NIST felt it was worthwhile to discuss Ref. [54], keeping in mind that the values for MARS may be different for the Round 2 version.

##### 3.4.2.1 Notes on the Finalists

MARS has a relatively large ROM requirement. Its key setup is very slow, and encryption is also slow, especially when the amount of RAM is constrained (this study only examined the original MARS submission, not the Round 2 version).

RC6 has a moderate ROM requirement. However, both key setup and encryption are slow, especially when amount of RAM is constrained. In addition, if the amount of RAM is constrained, then decryption will be very slow.

Rijndael has a low ROM requirement and very low RAM requirement. Both encryption and decryption are at least twice as fast as any other finalist.

Serpent has a low ROM requirement and moderate RAM requirement. Encryption is very slow.

Twofish has a very low RAM requirement, except possibly for the key schedule that is omitted for this study. The ROM requirement is fairly high. The speed is second best among the finalists, although only about half as fast as the Rijndael decryption speed.

##### 3.4.2.2 Comparison of the Finalists

It is difficult to draw any general conclusions about the finalists as a whole from this study, due to the incomplete treatment of MARS and Twofish (i.e., the Round 1 version of MARS is used, and the Twofish results have been quoted from statements made by the Twofish submitters). Rijndael emerges as the best, with a low RAM requirement and at least twice the encryption and decryption speed of any other finalist. Serpent is second from a space point of view, but is slow.

### 3.5 Hardware Implementations

While a relatively large amount of data was available on the performance of software implementations on various platforms, there was much less data available to NIST for hardware implementations. A software implementer is simply trying to efficiently express an algorithm in terms of an existing hardware device. However, a hardware implementer is designing a device to perform the algorithm and has far more degrees of freedom, but much more work to do to compare each alternative explored.

This is particularly true when attempting to exploit the parallelism of an algorithm. The software implementer simply tries, as best he can, to use the available execution units of a processor to maximize performance. The hardware implementer has many options to design the hardware to best exploit the inherent parallelism of an algorithm.

Like software, hardware implementations can be optimized for speed or for size. However, in the case of hardware, size translates much more directly into cost than is usually the case for software implementations. Doubling the size of an encryption program may make little difference on a general-purpose computer with a large memory, but doubling the area used in a hardware device typically more than doubles the cost of the device.

Two major classes of hardware devices, Field Programmable Gate Arrays and Application Specific Integrated Circuits were studied during Round 2 and are discussed separately in Sec. 3.5.3 and 3.5.4. First, however, some architectural considerations that apply to both classes of hardware are explored.

#### 3.5.1 Architectural Options

There are many possible approaches to the hardware design of the finalists. Some of the major options are explored below. The suitability of a particular option depends upon the specific environment, including space and other available resources.

Another major consideration is the intended cryptographic mode of operation. The two categories are the feedback and non-feedback modes. In both modes, plaintext consists of a sequence of blocks to be processed. In the feedback modes (e.g., Cipher Block Chaining, Cipher Feedback, and Output Feedback), the encryption or decryption of the next block cannot begin until the present block has finished. This prevents the parallel processing of separate blocks. In the non-feedback modes (e.g., Electronic Code Book, counter mode or interleaved modes), this restriction is removed, and blocks can be pipelined and/or processed in parallel, achieving substantially higher throughput performance. However, for feedback modes, parallel encryption of separate plaintext blocks is not possible; pipelining yields no performance gain and may cause a substantial increase in the area required to implement the algorithm.

##### 3.5.1.1 The Basic Architecture

If an algorithm has homogeneous rounds, the simplest implementation of encryption or decryption in hardware is to implement one round in combinational logic. Combinational logic circuits are circuits whose current output state is solely dependent on the present state of the circuit inputs. In a system clock cycle, data is fed to the circuit implementing that round via a multiplexer, and the output of the circuit is stored in a register. Thus, in each system clock cycle, one round of the algorithm is evaluated. If the algorithm uses *k* rounds, it takes *k* clock cycles to encrypt one block. This configuration is often referred to as the *basic architecture.* An advantage of this architecture is the minimization of the area required for round function implementation. However, additional hardware is required for subkey multiplexing (and multiplexing of other large data objects such as S-boxes, if applicable). The basic architecture is versatile; it may be used in either feedback or non-feedback modes. It is also fairly efficient, since the logic implementing the round is in continuous usage. However, not all parts of this logic are in use at any given time during a cycle. Some optimization may be attainable by exploiting this fact, as noted in Sec. 3.5.1.2.

If an algorithm has heterogeneous rounds, the basic architecture will have to be modified accordingly. Each type of round will have to be implemented.

As discussed in Sec. 3.8, an algorithm may have an option for generating subkeys on-the-fly. Another possibility is to perform subkey computation in hardware prior to encryption or decryption, and store the subkeys in registers or RAM. A third possibility is to have subkey computation performed externally, and subkeys stored prior to the processing of the data blocks.

##### 3.5.1.2 Internal Pipelining

The basic architecture can be modified, in some cases, by partitioning the logic within a round, with registers separating the individual portions of the rounds. It may be possible to do so in such a fashion that the latencies associated with the portions are roughly equal. In this event, the portions can form the stages of an internal pipeline within the round, with each stage executed in one system clock cycle. This presents an opportunity for instruction-level parallelism: if there are *k* stages, *k* blocks of data can be processed in parallel. However, pipelining can only be fully exploited when non-feedback modes are used.

Internal pipelining can increase throughput and efficiency considerably in non-feedback modes. Throughput (i.e., the number of blocks processed per second) increases almost linearly with the number of stages, but area requirements presumably increase at a much lower rate, since the only requirement is the addition of some registers. However, the potential for internal pipelining is generally limited, since it is necessary to subdivide a round into portions with similar delays. Thus, in this form of pipelining, the number of stages is generally small.

##### 3.5.1.3 Loop Unrolling

Another variation on the basic architecture is loop unrolling. In this case, *k* copies of a round are used, where *k* is usually a divisor of the number of rounds. All *k* rounds are implemented as a single combinational logic. Loop unrolling increases both the area devoted to round logic and the system clock cycle by a factor of roughly *k*. Furthermore, *k* subkeys must be stored prior to each clock cycle, possibly increasing the number of registers needed for this purpose. Loop unrolling can be used to increase throughput in a feedback mode. However, with loop unrolling, the area requirement usually increases more than the increase in throughput, resulting in lower efficiency.

##### 3.5.1.4 External Pipelining

Another variation on the basic architecture is attained by placing registers between *k* unrolled rounds. Then the *k* rounds form the stages of a pipeline (technically the registers, rather than the logic implementing the rounds, form the stages). As in the case of internal pipelining, this option can be fully exploited only in non-feedback modes. External pipelining increases the area required by a factor of roughly *k*. Included in this increase is often a *k*-fold replication of data objects such as S-boxes. On the other hand, in non-feedback modes, throughput increases by a factor of roughly *k* as well.

##### 3.5.1.5 Hybrid Pipelining

Both external and internal pipelining can be implemented simultaneously. That is, *k* rounds of an algorithm can be replicated. In turn, each can be subdivided into *m* portions. Altogether, a pipeline of *k×m* stages can be formed.

#### 3.5.2 Design Methodologies and Goals

There are two basic approaches to hardware design: low-level and high-level. High-level design, which is language-based, tends to be predominant because of practical considerations. The result of high-level design is implementations that are relatively easy to produce, but may not be as optimal as would be the case with a low-level (schematic-based) design methodology. All of the studies reporting on the hardware implementations of AES finalists were high-level designs. In most cases, the reported performance data were the result of device simulations, rather than measurements performed on the actual devices. Tools for designing and simulating FPGAs and ASICs are mature and reliable. However, the tools use conservative design rules and assumptions, therefore the attainable clock rates of actual devices may sometimes exceed the predictions of the simulations.

Another consideration is the implementation goals. Possible goals include:

- Minimum area.
- Maximum throughput, with unlimited area.
- Maximum throughput within a fixed area.
- Maximum efficiency, as measured by throughput/area.

Generally, the goal determines the design approach; different goals often produce incompatible design decisions. In particular, area minimization and speed maximization are generally polar opposites. The maximization of speed may affect efficiency. For example, full loop unrolling (all rounds unrolled) may maximize throughput but increase the required area and reduce efficiency. On the other hand, in non-feedback modes, pipelining may increase throughput but keep efficiency essentially constant (in the external case—see Sec. 3.5.1.4) or increase efficiency (in the internal case—see Sec. 3.5.1.2).

#### 3.5.3 Field Programmable Gate Arrays

A Field Programmable Gate Array (FPGA) is an integrated circuit consisting of a large two-dimensional array of small computing units that can be programmed. Data can be routed within the array, vertically or horizontally. Altering connections between the units can effect re-routing. This type of hardware offers the advantages of flexibility, low development cost, and low product cost for relatively low volume devices. In particular, it is possible to reconfigure an FPGA to switch from one algorithm to another algorithm, or from encryption to decryption or key setup. Reconfiguration takes only a fraction of a second. However, the flexibility advantages are traded off against speeds lower than those attainable by non-reconfigurable hardware devices (e.g., ASICs). On the other hand, FPGAs can achieve speeds considerably higher than software implementations.

The computing units of an FPGA are sometimes called Configurable Logic Blocks (CLBs). Reconfiguration changes the functions of the CLBs and the connections between them. A CLB typically consists of lookup tables and flip-flops. The lookup tables are configured as either a small amount of combinational logic or a small RAM.

An FPGA may also contain embedded RAM blocks that can be employed as either lookup tables or memory elements. However, there is a considerable variation between FPGAs in this regard, and the use of embedded RAM may affect portability and the universality of the results obtained. In addition, RAM has slower access time than CLBs.

Subkey computation can be implemented within the FPGA, in which case the array could be configured for subkey computation, and then reconfigured for encryption or decryption. Alternatively, subkey computation could be performed externally, and the subkeys could be loaded through the input interface prior to the processing of the blocks.

In order to hold down time and cost, all FPGA investigations of the finalists had to focus on certain priorities. For example, some researchers implemented only encryption. Others implemented a wider scope of functions, but did not explore as wide a range of architectures. All four of the FPGA case studies presented in this report used 128 bit keys.

Unless otherwise noted, all case studies were implemented using VHDL^3^ and simulated on a general-purpose computer for the specified FPGA. These simulations incorporated all device and design specific timing constraints.

##### 3.5.3.1 Operations and Their Implementation

The operations used by the finalists are summarized in Table 7. Note that the finalists can be implemented using either table lookups, or both Boolean and fixed shift operations. S-boxes can be implemented via combinational logic or embedded RAM. The XOR, mod 2^32^ add and subtract, and fixed shift operations are fast and use few hardware resources. The GF(2^8^) multiplications used by the finalists are also efficient. The most costly operation in terms of both area and time is mod 2^32^ multiplication.

##### 3.5.3.2 A Case Study

In Ref. [30], all finalists except MARS were implemented using FPGAs. However, only the encryption function was implemented. It was assumed that subkeys were generated externally, loaded from the external key bus, and stored in internal registers before encryption began.

The target FPGA was the Xilinx Virtex XCV 1000BG560-4. This device has 128 kbit of embedded RAM, 512 I/O pins, and a 64×96 array of CLBs. Language-based design methodology was used, with VHDL being the chosen language. Synthesis tools (FPGA Express by Synopsis, Inc. and Synplify by Synplicity, Inc.) were set to optimize the implementation for speed. For internally pipelined architectures, a 40 MHz timing constraint was used.

Note: The terms *basic architecture, external pipelining*, and *internal pipelining* are referred to in Ref. [30] as *iterative looping, partial pipelining*, and *sub-pipelining*, respectively.

###### 3.5.3.2.1 Notes on the Four Finalists Implemented

RC6: Although RC6 can use a mod 2^32^ multiplier, a simpler structure called an array squarer was all that was needed. The array squarer reduced the logic required for round function implementation. Nonetheless, squaring was the dominant operation in terms of time and space. Although the fast 32 bit variable rotation used by RC6 is potentially resource intensive, the implementers found that a simple five-stage 32 bit barrel shifter was sufficient. Full loop unrolling and full external pipelining were infeasible, due to space constraints.

In a feedback mode, 2-round external pipelining was found to yield the highest throughput. This implementation increased the system clock frequency and throughput by about 50 % over the basic architecture, while increasing the area by only about 20 %. In contrast, 2-round loop unrolling increased throughput by only about 8 % over the basic architecture, while using nearly as much area as 2-round external pipelining. External pipelining using more than 2 rounds was counterproductive with respect to throughput, as was the use of all forms of internal pipelining and hybrids of external/internal pipelining.

In a non-feedback mode, it was found that about 2/3 of the delay of the round function was caused by mod 2^32^ multiplications. Partitioning the multiplier and adding two internal pipeline stages nearly tripled the system clock frequency. Combining this with 10-round external pipelining resulted in a throughput that was more than 27 times greater than the basic architecture. However, this required more than a four-fold increase in area requirements. Further internal pipelining was counterproductive due to the atomic nature of the multiplications, and further external pipelining was infeasible due to space constraints.

In both feedback and non-feedback modes, the area requirement was minimized by the basic architecture.

Rijndael: In this case study, the S-boxes predominated in terms of required logic resources. Each S-box is an 8 bit by 8 bit lookup table, and 16 copies are required per round. Other operations were simple. Full loop unrolling and full external pipelining were infeasible due to space constraints.

In a feedback mode, 2-round loop unrolling attained the best throughput. However, the improvement in throughput over the basic architecture was slight, while the increase in area was about 50 %. All forms of pipelining produced smaller throughputs. The use of one internal pipeline stage lowered the area requirement compared to the basic architecture, but also lowered throughput by about the same proportion.

In a non-feedback mode, about half of the round function delay was produced by S-box substitutions. Adding one internal pipeline stage to separate the S-boxes nearly doubled the system clock frequency. The highest throughput was obtained from 5-round external pipelining combined with one internal pipeline stage. This increased throughput by a factor of about 7 over the basic architecture, while roughly doubling the area requirement. Further internal pipelining was not explored; further external pipelining was infeasible due to space constraints. Adding one internal pipeline stage to the basic architecture minimized the area requirement. This also increased throughput by about 70 % over the basic architecture.

Serpent: The small size of the S-boxes permitted implementation via combinational logic. In addition, the S-boxes matched well with the Xilinx CLBs. Other operations were simple. The Serpent round function is so compact relative to the other three analyzed finalists that it was the only one of the four implemented finalists that was amenable to full loop unrolling and external pipelining (i.e., all 32 rounds).

In a feedback mode, 8-round loop unrolling attained the highest throughput. This reduced the need for S-box multiplexing hardware. Throughput increased by nearly a factor of 8 over the basic architecture, while the area increased by only about 40 %. However, 32-round loop unrolling, while feasible, lowered the system clock frequency and throughput. External pipelining was counterproductive.

In a non-feedback mode, 32-round external pipelining produced the highest throughput, about 8 times greater than the basic architecture, while increasing the area by about 80 %.

In both feedback and non-feedback modes, the area was minimized by the basic architecture. The round function components are simple enough that internal pipelining was counterproductive.

Twofish: The predominant feature of the Twofish round function is the key-dependent S-boxes. The S-boxes produced about half the delay. Internal pipelining with one stage was feasible, separating the S-boxes. Full loop unrolling and full pipelining were infeasible.

In a feedback mode, the highest throughput was obtained by adding one internal pipeline stage to the basic architecture. However, this increased throughput by only about 15 %, with a similar increase in the area requirement. External pipelining was counterproductive.

In a non-feedback mode, the highest throughput resulted from 8-round external pipelining, combined with one internal pipeline stage. This increased throughput by about a factor of 15 over the basic architecture, while increasing the area by about a factor of 3. Further external pipelining was infeasible due to space constraints.

In both modes, area was minimized by the basic architecture.

###### 3.5.3.2.2 Comparison of the Four Implemented Finalists

As noted in Sec. 3.5.2, there is at least four different ways that the finalists could be evaluated. One of these, maximum throughput with unlimited area, is not applicable in the context of the present study, since only Serpent has a round function that is small enough to permit full loop unrolling and external pipelining. Area measurements are made in terms of CLB slices (a slice is half of a CLB).

The constraints on the available area could be interpreted in two different ways. One viewpoint is to look at the maximum throughput attained in the available space. However, this is device-dependent. In some cases, a more portable measurement is efficiency, as measured by throughput/area. This could provide some insight into what might be achievable if the space constraint were loosened or removed. However, there is also a limitation on this measurement: throughput may not scale linearly with increases in the number of rounds in loop unrolling. This is clearly illustrated in the case of Serpent, where 8-round loop unrolling is superior to 32-round unrolling. It is dangerous to try to extrapolate measurements from one FPGA family to another, or even within the same family.

In addition to the omission of MARS and the implementation of encryption only, a significant constraint of this study is that the synthesis tools were set to optimize for highest throughput. If minimum area or optimum efficiency were the goal, synthesis tools would have to be reset accordingly. Thus, this study provides an accurate comparison between the four implemented finalists only with respect to throughput optimization. The results are given in Table 8; these results are based on Tables 4 and 5 of Ref. [30]. It should be noted that the latter two tables are optimum throughputs from the much more comprehensive Tables 2 and 3 of Ref. [30]. Implementations included encryption only, and were optimized for speed. Thus, different architectural options are represented in the table.

The interpretation of the results is dependent upon the mode of operation used and the implementation goals. If a feedback mode is assumed, Serpent provides the highest throughput (at least 45 % better than any of the other three implemented finalists). However, this throughput is attained at a high area cost. In terms of efficiency, Serpent and Rijndael are virtually identical, with RC6 and Twofish both about 30 % behind.

In a non-feedback mode, Serpent has an unequivocal edge over the competition, providing more than twice the throughput of any of the other three finalists and, at the same time, occupying the smallest area.

The authors conclude that among the four implemented finalists, Serpent is, in general, the best suited to FPGA implementation. This is due mainly to the use of small S-boxes, which increases efficiency in loop unrolling and makes full external pipelining feasible. However, it should be kept in mind that these conclusions are specific to the device and the implementation design goals.

##### 3.5.3.3 A Second Case Study

In Ref. [38], all five of the finalists were implemented, again using Xilinx FPGAs. Two different Xilinx families were employed: the Virtex XCV1000BG560-4 and the smaller XC4000. The CLBs of the two families are nearly identical from a functional point of view. Language-based design was employed, with VHDL being the chosen language. The exact Xilinx synthesis tools used are not specified.

Subkey computation was not implemented. It was assumed that subkeys were generated externally and stored in internal memory. Both encryption and decryption were implemented in units co-existing simultaneously within the FPGA. One advantage of simultaneous implementation (as opposed to reconfiguration) is efficiency: resources can be shared between the two units in most cases. The design paradigm used was to maximize such resource sharing. This decreased both the speed and the total area for the encryption and decryption units. In the case of MARS, RC6 and Twofish, an encryption/decryption pair took up only about 10 % more space than a dedicated encryption unit. However, the increase was 60 % for Rijndael and 100 % for Serpent when both encryption and decryption were implemented.

For each finalist, only one architectural option was implemented; this was chosen to be as close as possible to the basic architecture. The basic architecture was implemented for RC6, Rijndael, and Twofish. For Serpent, 8-round loop unrolling was used. This was motivated by the fact that Serpent has eight kinds of rounds, with each type using a single replicated S-box. MARS uses two kinds of rounds, keyed and mixing. This necessitated the use of two separate round implementations that share resources such as S-boxes.

Other architectural options such as pipelining are discussed, but were not implemented. In particular, internal pipelining was analyzed in terms of what the authors designate as the critical path. This path of operations, shared by encryption and decryption, determines the minimum clock period of the system. A detailed study was made of critical paths, shedding some light on the area required and the potential for internal pipelining. The treatment of loop unrolling and external pipelining was largely speculative, and has been superceded by the actual implementations of the first case study (see Sec. 3.5.3.2).

Only two of the finalists were able to fit on the largest XC4000 FPGAs. Hence, these results are omitted from this report. All five finalists were implemented in Virtex FPGAs. The largest Virtex device (the XCV1000BG560-4) was used; all results below pertain to the Virtex device. Actually, at most 38 % of the available CLBs were used in implementing the basic architecture (or its approximation, in the case of MARS and Serpent). Hence, smaller members of the Virtex family could be used with this choice of architectural option. Alternatively, on the XCV1000BG560-4, key setup could be implemented in addition to encryption and decryption.

Note: The terms *internal* and *external pipelining* are referred to in Ref. [38] as *outer-round* and *inner-round pipelining*, respectively.

###### 3.5.3.3.1 Notes on the Finalists

MARS: MARS fared well when measured by the hardware that needed to be added to the encryption hardware to support decryption: two 32 bit subtracters and three 32 bit, 2-input multiplexers. The extra hardware needed for both encryption and decryption increased the required area by only about 10 % beyond that needed for encryption alone.

MARS had the slowest minimum clock period of the five finalists. It used mod 2^32^ multiplications, which are atomic and slow, and limit internal pipelining potential. An additional source of delay in MARS was the resource sharing between the forward and backward keyed transformations. On the other hand, resource sharing between encryption and decryption (one encryption/decryption multiplexer) on the critical path caused a negligible delay.

In addition to being slow, MARS also fared poorly in the area requirement, due to the heterogeneous round structure, the use of large S-boxes, and the 32 bit multiplications. The S-boxes were the major contributor to the large area requirement.

RC6: RC6 also fared well when measured in terms of the hardware that needed to be added to the encryption hardware to support decryption. A 32 bit subtracter, two 32 bit, 2-input XORs, and eight 32 bit, 2-input multiplexers were required. The extra hardware needed for both encryption and decryption increased the area requirement by only about 10 % over and above the space needed for encryption alone.

Operations dominating the area were two mod 2^32^ squarings, twelve 32 bit multiplexes, and two variable rotations of 32 bit words. The squarings, which are atomic and slow, dominated the round function delay and limited the internal pipelining potential. This observation agreed with that of the first case study (see Sec. 3.5.3.2). Resource sharing caused additional delay, due to the presence of three encryption/decryption multiplexers on the critical path. RC6 ranks fourth in terms of the minimum clock period.

While the squaring and variable rotation operations were slow, they were not excessively large, resulting in a relatively low area requirement (second best among the finalists).

Rijndael: In Rijndael, the encryption and decryption units are more separate than in RC6 or MARS. The units shared 16 lookup tables that implement inversions in GF(2^8^). Otherwise, the units were independent. Since the lookup tables took up about 40 % of encryption area, implementing both encryption and decryption required about 60 % more hardware than encryption alone.

Rijndael has the lowest clock period of the finalists. Its critical path contained only fast operations, with the greatest delay produced by S-box accesses. The large data objects needed, together with limited resource sharing between encryption and decryption, required a relatively large area. The potential for internal pipelining was limited by the atomic nature of the S-box accesses. This observation agreed with the first case study.

The area required by Rijndael was adversely affected by the limited resource sharing between encryption and decryption, as well as by the use of relatively large S-boxes. The area requirement is, in fact, the polar opposite of speed: Rijndael had the largest area requirements of the finalists.

Serpent: Serpent allows the least resource sharing of the finalists; the encryption and decryption units are virtually independent. Thus, implementing both encryption and decryption took roughly double the area needed for encryption alone.

In this study, eight “official” rounds of Serpent were regarded as one “implementation round.” This had the effect of stretching out the system clock, compared to the other finalists, so that the clock period appeared to be slow. However, with a relatively slow clock and only four implementation rounds, Serpent had a higher throughput in this study than any other finalist.

Round function delay was dominated by S-box accesses. The authors note that Serpent had a considerable potential for internal pipelining; however; this was a consequence of combining eight normal Serpent rounds into a single implementation round.

Serpent’s area requirement was adversely affected by the lack of resource sharing between encryption and decryption and the use of S-boxes. In addition, 8 of the 32 “normal” Serpent rounds were included in this variation of the standard model. Thus, Serpent fared relatively poorly in area requirements.

Twofish: Twofish fared best among the finalists when measured by the extra hardware required to implement both encryption and decryption: two 32 bit, 2-input XORs and two 32 bit, 2-input multiplexers. This increased the area by only about 10 % above that needed for encryption alone.

Twofish had the second best minimum clock period (i.e., the second fastest), only about 10 % greater than Rijndael. Like Rijndael, the critical path of Twofish contained only simple operations. As in the case of Serpent, the authors noted that there was some potential for internal pipelining. However, the pipelining was not explored empirically, so the first case study (see Sec. 3.5.3.2) is a better source of analysis in this regard.

Twofish had the smallest area requirement of the finalists, due to a high degree of resource sharing between encryption and decryption and the use of simple operations.

###### 3.5.3.3.2 Comparison of the Finalists

The Virtex speed and area measurements are presented in Table 9; these measurements were taken from Fig. 10 of Ref. [38]. Implementations included both encryption and decryption, using only one architectural option (mainly the basic architecture, with appropriate modifications for MARS and Serpent). Only a feedback mode was considered.

As in the first case study (Sec. 3.5.3.2), the interpretation of the results depends on the modes of operation used and the goals. Since the implementations in this second case study were essentially restricted to the basic architecture used in a feedback mode, the scope of the study is not as comprehensive as in the first study. In addition, the results are not directly comparable to the first case study. The first case study implemented only encryption; the emphasis in the present study is on the simultaneous implementation of encryption and decryption.

As in the first study, Serpent emerged as the fastest finalist, achieving a throughput at least 45 % higher than any other finalist. Minimum clock periods were not used in Table 9, in deference to the deviations from the basic architecture for Serpent and MARS. As noted in Sec. 3.5.3.3.1, this affects the ranking of Serpent considerably, and makes throughput the better metric. The fast speed of Serpent was traded off against area: Serpent used over four times as much area as Twofish, partly because of near-zero resource sharing between encryption and decryption.

Twofish used a very small area, partially as a result of near-100 % sharing of encryption and decryption resources. Thus, Twofish emerged as best in terms of both area requirements and efficiency, achieving a throughput/area ratio at least 60 % higher than any other finalist. The efficiency of the two fastest finalists, Rijndael and Serpent, was reduced by their large area requirements.

Comparison of Tables 8 and 9 reveals several other observations. The restriction to the basic architecture in a feedback mode caused a much greater variation between the speeds of the finalists. The inclusion of both encryption and decryption punished the non-Feistel algorithms (Rijndael and Serpent) with respect to area requirements. Furthermore, this second study helped to fill in the gap created by the omission of MARS from the first case study. In this second study, MARS finished at the bottom in both speed and required area (i.e., low speed and high area requirement), and fared very poorly on efficiency.

##### 3.5.3.4 A Third Case Study

In Ref. [27], all five finalists were implemented on the Xilinx Virtex family of FPGAs. Language-based design was used, with the Foundation Series v2.1i tool used for mapping onto the devices.

This third study analyzed key setup, which was not treated in the first two studies. Two functions were implemented simultaneously in separate units: encryption, referred to as the cryptographic core, and the key-setup circuit. Simultaneous implementation allows key setup and encryption to be done in parallel.

Performance is measured in two ways: throughput and “latency.” Throughput measures the data encrypted per a unit of time, while latency, in this study, is the time delay required for key-setup circuit before the cryptographic core can begin processing data. Latency becomes an important factor when only small amounts of data are processed per key. This distinguishes the third study from the previous two studies, in which the throughput was the major time metric. Low latency and high throughput are generally preferred in implementations.

Only a feedback mode is considered, and only the basic architecture is implemented for each finalist, with no deviations (except for the implementation of one of each of the two round types of MARS). The key-setup circuit uses embedded RAM to pass subkeys to the cryptographic core. The cryptographic core can begin processing as soon as the first subkey is available. Two different system clocks are possible, one for the cryptographic core and another for the key-setup circuit, and in some cases this reduces latency somewhat.

###### 3.5.3.4.1 Notes on the Finalists

MARS: MARS had by far the highest latency, as well as the highest ratio of latency to block encryption time. The use of two clocks improved latency somewhat; however, latency remained significantly greater than the other algorithms. Key setup involved string-matching operations that are relatively slow and expensive. Most operations of the cryptographic core were simple. The exception was multiplication, which required a multiplier computing partial results in parallel. The area requirement is high for both the key-setup circuit and the cryptographic core (not to be confused with the inner 16 rounds of MARS, designated as the cryptographic core in the MARS submission). Throughput is average.

RC6: RC6 permits very compact implementations of both the key-setup circuit and the cryptographic core, despite the necessity of a 32 bit multiplier. RC6 has moderate latency but below average throughput. Employing separate clocks for the key-setup circuit and the cryptographic core reduced latency by a factor of nearly three.

Rijndael: A ROM-based, fully parallel lookup-table oriented implementation gave low latency and high throughput. This results, however, in a moderately high area. Using separate clocks for the key-setup circuit and the cryptographic core reduced latency somewhat.

Serpent: Serpent has low latency. Although the Serpent round function allows a fast clock, throughput is only average, due to the large number of rounds. Serpent had the most compact implementation of the algorithms.

Twofish: The Twofish key schedule allows a range of space/time tradeoffs. The authors of the paper decided to achieve the lowest possible latency. This was effected by replicating permutation boxes and maximum distance separable (MDS) matrices. As a result of these design decisions, the Twofish key-setup circuit occupies by far the most area of any finalist, and requires far more space than the cryptographic core. However, area minimization would evidently have resulted in excessive latency. The net result was moderate latency at high area cost; throughput is average.

###### 3.5.3.4.2 Comparison of the Finalists

As in the two previous studies (Sec. 3.5.3.2 and 3.5.3.3), the conclusions reached about the candidates are heavily dependent upon the design paradigm. In this third study, the focus of attention was minimizing latency. This resulted in some tradeoffs between space and latency that make the results of this study, summa rized in Table 10, difficult to compare with the results of the previous studies (as summarized in Tables 8 and 9). In this table, the implementations included encryption and key setup. Only the basic architecture was used, with no deviations. A feedback mode was assumed.

MARS has a much higher latency than the other finalists. The other four finalists are much closer together with respect to latency. Rijndael has the smallest latency, followed closely by Serpent.

Rijndael has the best throughput, at least 75 % better than any other finalist. However, Serpent achieves the best ratio of throughput to area. Serpent has the smallest total area, followed closely by RC6. Unless area minimization is a paramount consideration, Rijndael emerges as the overall best finalist in this study.

##### 3.5.3.5 A Fourth Case Study

In Ref. [93], all five finalists were studied from the point of view of a “potential FPGA implementer.” Analyses, which are quite detailed, were nonetheless referred to as implementation sketches. The target FPGA family was the Xilinx Virtex.

The notation used in Ref. [93] is somewhat different than that used in the previous studies (Secs. 3.5.3.2–3.5.3.4). The authors considered the use of a microcoded datapath, that is, a processor designed for the application and driven by a small microprogram. They found this primarily applicable to key setup. The authors also used “C-slow” to refer to C-stage pipelines formed from one or more rounds. This appears to be similar to the approach of the second study (Sec. 3.5.3.3): the only case in which multiple rounds were used is Serpent, in which eight rounds form an 8-stage pipeline. In other cases, a round or half-round was internally pipelined. The use of half-rounds to implement rounds (applicable to RC6 and Twofish) resulted in an option called a “folded round.”

This fourth study analyzes encryption, decryption, and key setup, and hence has something in common with each of the three previous studies (Secs. 3.5.3.2–3.5.3.4). However, the architectural options considered in this study seem to be somewhat out-of-synch with those used in the previous studies. For example, Fig. 1 of Ref. [93] contains some information similar to that found in Ref. [30]; however, it is difficult to reconcile the two treatments. Since the treatment of Ref. [30] is based on VHDL implementations, and the treatment in this fourth study appears to be more sketchy, it is assumed in this report that the treatments of Ref. [30] take precedence when Refs. [30] and [93] do not agree.

On the other hand, this fourth study provides a more detailed investigation of subkey computation than any of the previous studies. In particular, the authors considered several architectural options for the circuit used for subkey computation, which was assumed to be implemented along with encryption or decryption units. In some cases, it was possible to run subkey computation concurrently with encryption, decryption or both.

###### 3.5.3.5.1 Notes on the Finalists

MARS: Subkey computation in MARS was slow. The generation process required sets of subkeys to be modified several times. This effectively precluded pipelining or other methods for acceleration. The authors state that the best implementation option was a custom microcoded datapath. This resulted in a reasonably compact area requirement.

RC6: RC6 also had slow subkey computation. The serial nature of the generation process again precluded pipelining or other forms of acceleration. A custom microcoded datapath is recommended, requiring a reasonably compact area.

Rijndael: Rijndael permits subkey computation concurrently with encryption, but not for decryption. Subkey computation was compact and amenable to pipelining to some degree that was not precisely specified. Efficient subkey computation produced fairly low encryption latency. However, for decryption, subkey computation required either buffering or generating the entire key schedule before decryption could begin. S-boxes could be independent or shared with the encryption pipeline. Either way, the area requirements were modest.

Serpent: As in the case of encryption, subkey computation in Serpent required many S-box accesses. Theoretically, the circuit used for subkey computation could share S-boxes with the encryption unit, but in practice, multiplexing would make this impractical. Instead, a separate, 8-stage pipeline is recommended to implement subkey computation. This pipeline allowed key setup to be concurrent with encryption, but not with decryption. As in the case of Rijndael, the buffering of subkeys was required for decryption. The pipeline required considerable area. Even if a smaller pipeline (2 rounds) were used, the required area would still be high.

Twofish: Twofish was ideal in cases where switching between encryption and decryption occur often. Subkeys can be generated independently in either direction; shared with the encryption and decryption pipelines. When shared S-boxes were used, latency was low, but area was moderately high. When separate S-boxes were used, latency was extremely low, but area was high.

###### 3.5.3.5.2 Comparison of the Finalists

Reference [93] actually discusses both encryption/decryption and key setup, although Sec. 3.5.3.5.1 focuses on key setup. A rough summary of the conclusions of this study concerning encryption and decryption, as embodied in Fig. 1 of [93], is as follows. Only one round of each finalist was generally implemented, with internal pipelining. An exception was Serpent, for which 8 rounds were implemented. Rijndael had the lowest latency, high throughput, and low area. Serpent achieved the highest throughput due to the 8 rounds, but also had a large area requirement. MARS was slow with respect to both latency and throughput; RC6 and Twofish were in between.

The results on key setup, as summarized in the Sec. 3.5.3.5.1, are tabulated in Table 11. Implementations giving the lowest latency are listed, with the highest bandwidth being the second priority; other implementations may have lower areas. Twofish emerged as by far the best finalist from the point of view of this fourth study, due to its independent generation of subkeys. MARS and RC6 were very slow, with Serpent and Rijndael in between.

##### 3.5.3.6 Overall Summary of FPGA Implementations

It is difficult to directly compare the results of the four previous studies (Secs. 3.5.3.2–3.5.3.5), since all made different assumptions about modes, architectural options, and the implementation of decryption and key setup. Tradeoffs between area and speed make it essentially impossible to condense hardware results into one dimension. Thus, it would be difficult to obtain anything analogous to the speed charts that are feasible in software for comparing the finalists. The best that can be accomplished for FPGAs is to profile the finalists individually.

MARS suffers from its large S-boxes and, to a lesser extent, from its multiplications, data-dependent rotations and heterogeneous round structure. MARS is generally characterized by high latency for subkey computation and low efficiency, as measured by throughput/area. The general opinion of the FPGA implementers is that MARS is the least suitable for FPGA implementation, often by a considerable margin.

RC6 suffers, to some extent, from the use of multiplications and data-dependent rotations. These operations tend to have a negative impact on throughput and latency. However, RC6 has no S-boxes, which has a positive effect on the area requirements. Overall, the design choice of using complex operations instead of S-boxes has a canceling effect, putting RC6 in the middle range of the candidates by most FPGA metrics provided during Round 2.

Rijndael has a low latency and the highest throughput of all the finalists in a feedback mode, and good throughput in a non-feedback mode. Fully unrolled Rijndael implementations have the highest throughput of any of the algorithms in feedback modes. Rijndael’s area requirements are increased by limited resource sharing between the encryption and decryption units, giving Rijndael moderately high area requirements in the general case, where both encryption and decryption must be supported. Rijndael’s efficiency is average or better.

Serpent has a low latency and allows the highest throughput of all the finalists in non-feedback mode, and good throughput in a feedback mode. Fully pipelined versions of Serpent have the highest throughput of the candidate algorithms for non-feedback modes. However, although reasonably compact implementations are possible, Serpent, often has high area requirements as well. These are interconnected: Serpent is most naturally viewed in this context in terms of 8-round groupings. These groupings increase the potential for pipelining and loop unrolling, but also increase the area requirements. Moreover, Serpent’s area requirements are increased by its lack of resource sharing between the encryption and decryption units. Serpent’s efficiency is average or better.

Twofish tends to be in the middle range with respect to both latency and throughput. Twofish’s area tends to be compact; however, it still tends to score in the low to moderate range in efficiency. The details vary considerably with the setting. If decryption is implemented simultaneously with encryption, Two-fish benefits from considerable resource sharing between the encryption and decryption units. If key setup is implemented simultaneously with encryption and decryption, Twofish benefits from its independent subkey computation.

#### 3.5.4 Application Specific Integrated Circuits

An Application Specific Integrated Circuit (ASIC) is not reconfigurable and cannot be altered once produced. ASICS can be economically produced only in large quantities; they can have far more gates and be much faster than FPGAs. However, there are many similarities, including architectural options and the general use of language-based design tools such as VHDL.

##### 3.5.4.1 A Case Study

In Ref. [94], all five finalists were implemented in CMOS^4^-based ASICs, using a 0.5 micrometer standard cell library that is not available outside the National Security Agency (NSA). VHDL was used to describe the finalists. The final output of the design process was a gate-level schematic for each finalist. The performance estimates are based on simulations.

Both the basic “iterative” architecture (see Sec. 3.5.1.1) and a full external pipelined version were implemented for each finalist. All three key sizes (128, 192, 256 bits) were implemented. Time and area results are reported for the case where all three key sizes were implemented in a single key-setup circuit that could be controlled. This flexibility reduced performance compared to the case where only a single size was implemented.

###### 3.5.4.1.1 Notes on the Finalists

MARS: The heterogeneous round structure of MARS caused some problems. There are six different round types altogether (unkeyed forward and backward, keyed forward and backward, pre-addition, and post-subtraction). This heterogeneity caused unusually complex synchronization of the key-setup circuit and encryption/decryption blocks.

The Round 2 modified version of MARS was implemented. This version permits generating and storing the 40 subkeys in groups of 10 during the encryption process. In the pipelined architecture, each group of 10 subkeys was stored, creating a 4-stage key schedule pipeline. The cryptographic core required only two subkeys at a time. Thus, some space was wasted on the storage of extra subkeys. Decryption required generating and storing all 40 subkeys in registers before processing began. Therefore, because both encryption and decryption were implemented, the decision was made to generate and store all 40 subkeys for the iterative architecture.

RC6: In RC6, resource sharing was used between encryption and decryption. The iterative architecture was straightforward. In the pipelined architecture, the key schedule was also pipelined, performing one subkey computation per clock cycle.

Rijndael: In Rijndael, the number of rounds was adjusted automatically with the key size. In the pipelined architecture, the key schedule was also pipelined. Similar functions could be used to generate subkeys for encryption or decryption; this aided Rijndael’s key agility. For decryption, the pipeline started with the last subkey; the generation of this subkey was essentially key setup time. For the iterative architecture, the key schedule was used to calculate all subkeys. The last subkey was stored in the case that multiple decryptions occurred with the same key.

Serpent: In the pipelined architecture, the key schedule was pipelined along with encryption or decryption. For decryption, all subkeys were first generated in order to obtain the last subkey. Four types of rounds were required, two for encryption and two for decryption. In the iterative architecture, all subkeys were generated in order to obtain the last subkey before decryption could commence.

Twofish: An advantage of Twofish is the substantial resource sharing between encryption, decryption and key setup. Sharing between encryption and decryption did not improve efficiency, since they were run in parallel. However, such sharing reduced design time, since blocks could be replicated. Other optimizations are possible with Twofish, but constitute area/time tradeoffs. The independent subkey computation property eliminates the setup time needed by decryption for the other finalists. In addition, encryption and decryption are almost identical. Both the pipelined and iterative architectures were straightforward.

###### 3.5.4.1.2 Comparison of the Finalists

The results of Ref. [94] are summarized in Table 12. In the basic architecture, Rijndael gave the best performance as measured by throughput/area. Serpent was close behind. RC6 and Twofish were substantially lower, and MARS was poor. Rijndael was best in terms of throughput; RC6, Serpent and Twofish were best in terms of their area requirements. In the fully pipelined mode, Serpent was best when measured by throughput/area. Rijndael was somewhat lower; RC6 and Twofish were considerably lower, and MARS was poor. Serpent was best in throughput, followed by Rijndael; all algorithms except MARS had small area requirements.

Key setup times were low for Rijndael, Serpent, and Twofish; they were high for RC6 and MARS.

##### 3.5.4.2 A Second Case Study

In Ref. [50], the finalists were implemented in Mitsubishi Electric’s 0.35 micrometer CMOS ASICs. The design library is publicly available.

The design philosophy was to optimize the encryption speed for feedback modes. The required area was not considered a significant metric. Full loop unrolling was used for encryption, decryption, and key setup. Prior to encryption or decryption, it was assumed that subkey bits had been stored in registers. No pipelining was used. Hence, one block was encrypted or decrypted in one clock cycle. Only 128 bit keys were used for this study.

No special optimization was used for the lookup tables. Performance depends on the optimization capabilities of the synthesis tool used. The design language was Verilog HDL^5^. Some operations, such as addition and multiplication, were taken from a library.

###### 3.5.4.2.1 Notes on the Finalists

MARS requires four types of round functions. The use of 16 multiplications in the critical path resulted in its slow performance. The multiplications accounted for about 2/3 of the delay. Key setup was about three times slower than encryption.

RC6 required 20 multiplications in its critical path, which resulted in slow performance. The multiplications accounted for about 3/4 of the delay. Key setup was about three times slower than encryption.

Rijndael is well suited to a hardware implementation. The Rijndael implementation used logical functions and lookup tables. However, there was an asymmetry between encryption and decryption. The inverse of the MixColumn operation used more complex constant values than the MixColumn. Hence, the critical path was longer for decryption. The table lookups accounted for about half the delay. Key setup took about 85 % as much time as encryption.

Serpent is very well suited to a hardware implementation. The Serpent implementation used linear transformations (XORs and shifts) and table lookups. The 32 tables were small. The linear transformations accounted for about 36 % of the delay, and the table lookups accounted for 45 %. The effect of the asymmetry of encryption and decryption is not discussed in Ref. [50]. Key setup took about 85 % as much time as encryption.

Twofish is moderately well suited to a hardware implementation. The Twofish implementation used 8 bit by 8 bit lookup tables, additions, and logical operations. The critical path had 48 lookup tables, which accounted for about half the delay. Key setup used only about 5 % of the time used for encryption.

###### 3.5.4.2.2 Comparison of the Finalists

A summary of the results of this case study is given in Table 13. The emphasis in this study was on encryption speed, although encryption, decryption and key setup were all implemented. The table clearly shows that Rijndael was the best in terms of both throughput and efficiency (throughput/area). The throughput of Rijndael was more than twice that of any other finalist, while its area requirements were only modestly higher. Serpent also fared well in both throughput and efficiency. Twofish was smaller than Rijndael or Serpent, but did not fare nearly as well in throughput or efficiency. MARS and RC6 were even lower for throughput, and fared very poorly for efficiency.

The authors [50] suggest some ways in which their results could have been optimized, at some expense. For example, a full-custom multiplier would reduce the critical path time of MARS by about 50 % and of RC6 by about 70 %. However, the efficiencies of MARS and RC6 would still be poor. An optimization of the lookup tables would not affect the rankings by much.

#### 3.5.5 Comparison of All Hardware Results

It is difficult to completely reconcile the results for ASICS and FPGAs. As noted in Sec. 3.5.2, there are at least four metrics that could be used to evaluate performance in a hardware context. Area requirements and efficiency apply to any hardware implementation. However, FPGAs are much more strongly constrained by area limitations. Thus, for example, Tables 2 and 3 of Ref. [30] are constrained by area limitations on all the finalists except Serpent, ruling out options such as full loop unrolling. On the other hand, Ref. [50] employed full loop unrolling for all the finalists. FPGAs impose a certain structure on the logic and memory, while ASICs are not similarly constrained. The studies did not usually include area minimization as a design goal.

Another problem in trying to reach conclusions about hardware in general is that the number of parameters (e.g., feedback vs non-feedback mode, implementation vs omission of decryption and/or key setup, speed or area optimization) overwhelms the available set of experiments. Thus, the set of studies to draw upon is somewhat diffuse, with different implementers employing different sets of options. This limits comparability.

The summaries below focus on throughput, area and efficiency. Key agility, which may be an important factor in some hardware applications, is treated separately in Sec. 3.8.

MARS required the largest area of any of the finalists, often by a considerable margin, in every study where it was implemented. This is apparently due to the large S-boxes, the key schedule, and the heterogeneous round structure. Its throughput in the studies was generally low. Therefore, its efficiency (throughput/area) was uniformly less than the other finalists.

RC6 can be effectively implemented in hardware. Despite the need to implement a multiplier or an array squarer circuit, fairly compact implementations are possible. In comparison to the other finalists, RC6’s throughput is generally average. RC6 seems to perform relatively better in pipelined implementations, useful mainly for non-feedback modes, than unrolled implementations that are optimized for feedback modes. This may be at least partially a result of its reliance on multiplication, a long latency operation that lends itself well to pipelining.

Rijndael allows high throughput designs for a variety of architectures, namely basic, pipelined and unrolled implementations. When fully unrolled, 128 bit Rijndael has the lowest single block encryption latency of any of the finalists and, therefore, the highest throughput for feedback mode encryption. In standard architecture implementations, the throughput of 128 bit implementations is also at or near the top. Most of the studies did not consider other key sizes. However, since Rijndael adds additional rounds for larger key sizes, throughput in the standard architecture or unrolled implementations falls with larger key sizes, but still remains good. For fully pipelined implementations, area requirements increase with larger key sizes, but throughput is unaffected. Rijndael has good performance in fully pipelined implementations, giving it non-feedback throughput performance that is second only to Serpent. Efficiency is generally very good.

Serpent uses eight different S-boxes for each of eight successive rounds, and repeats this four times for a total of 32 rounds. This, “major/minor” round structure offers two different interpretations of the basic architecture: one with one “minor” round repeated 32 times and multiplexing hardware for the S-boxes, and another with eight pipelined or unrolled minor rounds that are repeated four times. The first alternative allows compact implementations, while the second alternative eliminates S-box multiplexing and offers better throughput and efficiency. One round of Serpent has the smallest latency of any of the finalist algorithms; therefore, fully pipelined (32 stage) implementations of Serpent offer the highest throughput of any of the five finalists for non-feedback modes. Fully or partially (8 round) unrolled implementations of Serpent offer good, low latency single block encryption that is useful for feedback mode encryption. Serpent is second only to Rijndael in feedback mode throughput (that is, for the basic architecture and fully unrolled implementations). Efficiency is generally very good.

Twofish offers many implementation options that somewhat complicate analysis. However, throughput and efficiency in the basic architecture, pipelined and unrolled implementations was generally average. Most studies considered only 128 bit key sizes; however, Twofish’s throughput is reduced somewhat with larger key sizes in both the standard architecture and pipelined implementations. Compact implementations are possible.

In summary, MARS is not a good choice for hardware implementation in terms of either throughput or area requirements. Both RC6 and Twofish seem to have average throughput and efficiency. Rijndael and Serpent generally have the best throughput and efficiency of the candidates. Rijndael has the throughput advantage for feedback mode implementations, while Serpent has the throughput advantage for non-feedback modes. Rijndael’s 192 bit and 256 bit throughput is reduced somewhat for feedback modes from that of 128 bit implementations, while Serpent’s speed is independent of key size.

### 3.6 Attacks on Implementations

In Sec. 3.2, the general security of the finalists was discussed. The attacks discussed therein were essentially mathematical in nature and did not exploit the physical characteristics of the implementations. There is another class of attacks that use physical measurements conducted during algorithm execution to gather information about quantities such as keys. Such attacks exploit a combination of intrinsic algorithm characteristics and implementation-dependent features.

#### 3.6.1 Timing and Power Attacks

In some environments, timing attacks can be effected against operations that execute in different amounts of time, depending on their arguments. Power analysis attacks can be effected against operations that use different amounts of power, depending on their power consumption pattern, which may vary with the arguments to the operation. As noted in Sec. 3.6, the vulnerability of an algorithm to such attacks is generally a function of both the algorithm characteristics (e.g., the operations employed) and the implementation characteristics (which affect the feasibility of the physical measurements needed). This implies that some algorithms may be more suitable than others in certain environments in which certain attacks are feasible, depending on the cost of defending the algorithm against the particular attack. Defending against timing and/or power analysis attacks may involve some combination of physical defenses (e.g., increasing the noise to signal ratio of measurements) and various forms of modified implementation (e.g., changes in code).

#### 3.6.2 The Role of Operations

A general defense against timing attacks is to ensure that each encryption and decryption operation runs in the same amount of time. A possible defense against a power analysis attack is software balancing. In order to accomplish software balancing, the data to be treated (e.g., an intermediate result of the cryptographic algorithm) and the complement of that data are processed using the same basic operation as near-simultaneously as possible. In this way, the correlation to single bits of information is diminished. Software balancing may be effective for certain operations whose power consumption can be “masked” to some extent by executing the operation twice, employing the complement of the argument(s) during the second execution. A rough summary of the vulnerabilities of the basic operations used by the algorithms and their vulnerabilities to timing and power attacks is as follows [25]:

- Table lookup: not vulnerable to timing attacks; relatively easy to effect a defense against power attacks by software balancing of the lookup address.
- Fixed shifts/rotations: not vulnerable to timing attacks; relatively easy to effect a defense against power attacks by software balancing of the register containing the shift/rotate amount, and its complement.
- Boolean operations: not vulnerable to timing attacks; relatively easy to effect a defense against power attacks by software balancing of arguments.
- Addition/subtraction: somewhat difficult to defend against timing or power attacks by software balancing of arguments because of the propagation of carry bits.
- Multiplication/division/squaring or variable shift/rotation: most difficult to defend against timing and power attacks by software balancing of arguments because of intricate dependencies.

In the above list of operations, stating that a defense may be effected does not guarantee that a given operation is, in fact, protected; it merely means that such a defense is theoretically possible. Conversely, stating that an operation is difficult to defend against an attack does not imply that any given implementation of an algorithm employing that operation is vulnerable to attack.

A summary of the basic operations used for encryption by the finalist algorithms is given in Table 7 in Sec. 3.5.3.1, which was taken from Ref. [30]. Note that the GFmul operations can be implemented using table lookups, or both Boolean and fixed shift operations.

A rough summary of this information is as follows:

- Rijndael and Serpent use only Boolean operations, table lookups, and fixed shifts/rotations. These operations are the easiest to defend against attacks.
- Twofish uses addition, which is somewhat more difficult to defend against attacks.
- MARS and RC6 use multiplication/division/squaring and/or variable shift/rotation. These operations are the most difficult to defend.

Stating that an algorithm uses an operation that is difficult to defend does not mean that the algorithm is indefensible; “difficulty” refers to the complexity of the task of defense. For example, in some cases, software defenses may be sufficient to defend against a particular attack. In other cases, a software defense may be infeasible, and some form of hardware defense may be necessary. Furthermore, there is no guarantee that in a particular situation, any form of defense will work. That is, timing and power analysis are implementation-dependent attacks; the vulnerability of algorithms to such attacks is not an intrinsic algorithm characteristic.

#### 3.6.3 Implicit Key Schedule Weaknesses

A general question regarding an algorithm is the following. If an attacker gains access to a subkey (or, in some cases, a whitening key), does knowledge of this key yield information about other subkeys or the original key? If so, this might be termed an implicit (or perhaps conditional) key schedule weakness. This raises an issue that has had significant consequences in practice, particularly in connection with power analysis attacks. At the present time, two attacks are known that exploit implicit key schedule weaknesses; these are discussed below.

##### 3.6.3.1 A Power Analysis Variant

In Ref. [13], the authors employed a variant of power analysis to attack the finalists’ key schedules in environments in which power analysis is feasible. Their approach correlated the power consumed during the operation of the algorithm with the number of ones in a subkey byte. Evaluating the number of ones yielded an equation involving the bits of the original key, regarded as independent variables. A sufficient number of such evaluations may provide a system of equations that can be used to obtain the original key, assuming that the system of equations has a sufficiently high rank. The rank, in turn, depends upon the randomness of the process used to generate subkeys from the original key; conversely, redundancy in this process inhibits the attack by lowering the rank. However, even if the full original key cannot be recovered, it may still be possible to obtain some information about the original key.

If the power analysis attack in Ref. [13] could be effected, a rough classification of the finalists by key schedule is as follows:

- Some implicit weakness: MARS, RC6 and Rijndael. An attack may reveal some information about the original key.
- No weakness: Serpent and Twofish.

Even if the attack were feasible in some instance, the attack would only demonstrate the feasibility with respect to a given implementation of the finalist. Vulnerability to this type of attack is not an intrinsic algorithm characteristic. Thus, stating that a finalist has an implicit weakness that might be exploited under certain conditions simply means that certain defenses may be needed to defend against the attack. There might also be restrictions on the class of suitable implementations for a finalist. More generally, the algorithm characteristics, implementation characteristics, and usage scenarios may impact the feasibility of an attack.

##### 3.6.3.2 A Second Power Analysis Variant

In Ref. [16], another power analysis variant was employed to attack the algorithm key schedules in certain implementations. The attack exploited the particular operations used to generate subkeys. If some subkeys (or, in some cases, whitening keys) can be found, it may be possible to obtain information about other subkeys or the original key. The viability of the attack depends partially on the number of rounds that need to be attacked to obtain the sought-after information (e.g., subkeys). For this attack, the finalists may be classified roughly as follows:

- Most vulnerable: Rijndael, Serpent and Twofish. Their vulnerability springs from the derivability of the original key from a small number of subkeys or whitening keys. Only a small number of rounds need to be attacked.
- Less vulnerable: MARS and RC6. All or a large number of rounds need to be attacked.

Stating that a finalist is vulnerable to the attack presupposes that the attack is feasible. See Ref. [16] for a discussion of the precise conditions under which the attack is feasible. It should again be noted that vulnerability to such an attack is not an intrinsic algorithm characteristic, but rather is heavily implementation-dependent.

#### 3.6.4 Defenses Against Implementation-Dependent Attacks

Various mechanisms have been proposed to defend against timing and power analysis attacks. Proposed defense mechanisms include (e.g., Ref. [25]):

- Elimination of branching in program execution, to defend against timing attacks.
- Software balancing (e.g., using complements of arguments to even out the total power consumed).
- Algorithm design (e.g., avoiding operations that are difficult to defend, and avoiding implicit key schedule weaknesses).
- Hardware mechanisms (e.g., random noise production).
- Choice of implementation device.
- Operational defenses.

Notes on the proposed defense mechanisms: software balancing and algorithm design strategies were discussed in Sec. 3.6.2. Other mechanisms relate to the fact that the essence of most attacks is to collect statistical samples of quantities such as power consumption. Hardware defenses may raise the noise to signal ratio, requiring the number of samples to be higher.

The choice of implementation device is significant in several respects. First, high-end devices may have hardware defenses that are unavailable in lower-end devices. Second, attacks often model devices as finite-state automata, that is, a set of connected finite states. The difficulty in effecting an attack (reflected in the number of statistical samples of power consumption) may be related to the number of possible states of the device. The number of states determines, in part, the complexity of the state space that must be analyzed. The complexity may be greater for high-end devices.

Operational defenses also relate to the sampling phase of an attack. It may be possible to limit an attacker’s ability to obtain samples pertaining to one key (e.g., by limiting the number of encryptions that can be performed by one key). One method of defending against timing or power attacks is to physically protect the device. This is feasible for cases in which the owner of the device is not a potential adversary of the entity that places keys on devices (in particular, when the device stores only keys generated by the owner).

Practical defense systems are only beginning to be studied. One such study [21] is limited to bit-slice ciphers, and hence is only applicable to Serpent. It is still unclear as to what performance penalties would be entailed if the techniques described in this study were implemented. A more general case study is summarized below.

##### 3.6.4.1 A Case Study in Defense

In Ref. [65], a study was made of smart card implementations of the finalists. In this study, implementations were augmented with defenses against power analysis attacks. The performance degradation-caused by these defenses was measured. The results of this study are summarized in Table 14; these results were obtained from Table 2 of Ref. [65]. The study compared the results both with and without masking used as a defense against power analysis attacks.

The approach in Ref. [65] can be considered a generalization of software balancing. In software balancing, the bit-wise complements of data words are generated; in Ref. [65], random strings of bits, called masks, were generated to combine with the input data and key data. The fundamental algorithm operations were then carried out on the masked data, after which the masks were removed. Because different random masks were used for every execution of the algorithm, over a statistical sample, the power consumption should be uncorrelated with the secret key and the input and output data.

A general note on the results: the RAM requirements of all the finalists were out-of-range of typical low-end smart cards. The implementations were performed on a high-end, 32 bit ARM-based card. In the cases of four of the finalists (all but Twofish), the RAM requirements were similar, and the major distinctions came in speed and ROM requirements.

###### 3.6.4.1.1 Notes on the Finalists

MARS was the most difficult of the finalists to mask against power analysis attacks. The large size of the table used for table lookups created a problem, as did the masking of multiplications and frequent converting between Boolean and arithmetic masking. Consequently, MARS suffered severe performance degradation under masking, running nearly eight times slower than in the unmasked mode. Its masked mode speed was the slowest of the finalists, and its masked ROM requirement was second highest. The only strong point was a low RAM requirement in both masked and unmasked modes, but this resulted from the necessity for implementing the lookup table in ROM.

RC6 was easier to mask than MARS, but still suffered severe performance degradation under masking, running nearly eight times slower than in the unmasked mode. Problems resulted from the masking of multiplications and frequent changing from Boolean to arithmetic masking. On the other hand, RC6 had a high speed and very low ROM requirements in the unmasked mode. Thus, RC6, when implemented using the masked mode, finished in the middle of the finalists in speed and was still the lowest for ROM requirements. RC6 was second lowest in required RAM in the masked mode, but only by a small margin.

Rijndael was relatively easy to mask because it uses only Boolean operations and table lookups. The lookup table was small enough to be masked and stored in RAM. This resulted in a considerable increase in RAM requirements from the unmasked case, where it had the lowest RAM requirement, to the middle for the finalists for the masked case. All mask operations were independent of data, permitting a pre-calculation of the mask values. Rijndael was by far the fastest of the finalists in the masked mode, nearly three times faster than its nearest competitor. Rijndael was second lowest in its ROM requirement and third in its RAM requirement for the masked mode, but only by a small margin due to the very small unmasked requirement.

Serpent was implemented only in the bit-slice mode (the standard mode would have been inefficient and harder to secure). In the bit-slice mode, there was a considerable overhead due to the masking of Boolean operations. In some instances, masks could not be pre-calculated. Thus, Serpent suffered some performance degradation in the masked mode: Serpent’s speed was about three times slower in the masked mode than in the unmasked mode, finishing second slowest among the finalists for both modes. Serpent’s ROM requirement in the masked mode was the highest among the finalists, and its RAM requirement was second highest, though by only a relatively small margin compared to MARS, which required the least amount of RAM.

Twofish was relatively easy to implement in the masked mode. The masks were pre-calculated, and many masks were shared. The masked lookup table was stored in RAM. This resulted in 1) a considerable increase in the RAM requirement from that of the unmasked mode (where it had the second lowest RAM requirement), and 2) the highest RAM requirement among the finalists when the masked mode was used. Twofish’s masked mode speed was second among the finalists. Twofish was in the middle of the finalists in ROM requirements, which were still relatively low due to the placement of the masked table.

###### 3.6.4.1.2 Comparison of the Finalists

Overall, Rijndael appeared to offer the best combination of speed and memory usage when the masking approach specified in Ref. [65] was used to protect against power attacks. RC6 followed closely with relatively low memory requirements, although its speed was mediocre among the finalists. The other three finalists exhibited greater memory requirements: Twofish required almost 700 bytes of RAM, while MARS and Serpent required over 7400 bytes and 9500 bytes of ROM, respectively. Although Twofish and Serpent showed mediocre performance that was significantly slower than Rijndael, MARS displayed an exceptionally slow performance.

### 3.7 Encryption vs Decryption

For MARS, RC6, and Twofish, encryption and decryption use similar functions. For Rijndael and Serpent, encryption and decryption are distinct functions. This has some impact on the measurement of efficiency. Tables 16–21 of Appendix A provide encryption speeds, and Tables 22–25 provide decryption speeds. These speeds cannot simply be averaged, since there are some applications that require only encryption or decryption, but not both.

Another consideration is the extra space needed for decryption when the decryption function is different from the encryption function, and both must be included in an implementation. The amount of extra space needed to house both functions depends on the amount of shared resources (e.g., S-boxes) between the two functions. In addition, the significance of this penalty depends on how significant space requirements are and upon the total amount of space needed to house both functions. In some instances, it may be sufficient to implement only one function, in which case the issue of accommodating both the encryption and the decryption operations does not arise.

For each finalist, the encryption speed and decryption speeds are very similar. However, when key setup is included with encryption and decryption, there is a noticeable difference in the performance of Rijndael: decryption plus key setup is slower than encryption plus key setup. However, this difference becomes negligible when numerous blocks are processed. The other finalists do not display this difference in performance.

### 3.8 Key Agility

Key agility refers to the ability to change keys quickly and with a minimum of resources. This includes both subkey computation and the ability to switch between different ongoing security associations when subkeys may already be available.

There are two basic methods for key schedule computation: pre-computation with the storage of subkeys, and on-the-fly subkey computation (i.e., computation of the specific subkeys needed for a particular round just prior to use in the round). Rijndael, Serpent, and Twofish support on-the-fly-subkey computation. RC6 can support on-the-fly subkey computation for encryption, but not for decryption, due to the non-invertible nature of the subkey computation. MARS is a hybrid in that 10 of the 40 subkeys can be computed at a time.

In the case of finalists supporting on-the-fly subkey computation, a second consideration may arise concerning decryption. If the function used to generate subkeys is recursive (i.e., a subkey value is based on previous subkey value[s]) and the function is also invertible, a one-time execution of the key schedule must be performed fully in the forward direction (i.e., used for encryption) to obtain the last subkey. An application could save this last subkey as well as the first subkey for subsequent processing. Henceforth, the key schedule can be run in either the forward or reverse direction^6^ to perform on-the-fly subkey computation. This is the case for Rijndael. MARS also exhibits this property, but subkeys are computed 10 at a time. Serpent’s key schedule has the property that the last subkey can be computed directly from the original key, although the specification does not explicitly state this. On the other hand, Twofish permits subkeys to be computed in any order.

In cases where large amounts of data are processed with a single key, the time to execute the key schedule may be unimportant. On the other hand, in applications in which the key is changed frequently, key agility may be an overriding factor. (See Tables 26–30 in Appendix A. Note that this is the “Full Keying” option for Twofish.)

Subkey computation methods impact key agility. If subkeys can be computed on-the-fly, much less information is needed when switching between different security associations (i.e., between contexts). If subkeys cannot be computed on-the-fly, more information is needed to switch security associations rapidly. In this case, the additional information is stored off-line. If there are a large number of security associations, this will require a large amount of memory for storing the context information. A large amount of memory can be a significant cost in hardware implementations or any other implementations that require that all needed contexts be stored close at hand (e.g., in high-speed local or cache memory).

Based on these considerations, the finalists may be characterized as follows (key setup times are available in Tables 26–30 of Appendix A):

MARS requires the computation of 10 of the 40 subkeys at a time, requiring additional resources to store these 10 subkeys. In Ref. [46], three different levels of pre-computations to foster key agility are described. The option with the fastest key setup requires about 60 bytes of memory in addition to the storage of the original key; this is a detriment in memory-restricted environments or environments where memory is a costly commodity. MARS also requires a one-time execution of the key schedule to generate all subkeys prior to the first decryption with a specific key. The computation of multiple subkeys at one time uses more memory resources than are required for algorithms performing on-the-fly subkey computation of a single subkey.

RC6 supports on-the-fly subkey computation only for encryption, given intermediate values. The implementation indicated in Table 6 appears to compute the subkeys in this way, requiring about 100 bytes of RAM; this is a detriment in memory-restricted environments. The decryption subkeys must be pre-computed. The lack of on-the-fly subkey computation reduces the level of key agility afforded by RC6.

Rijndael supports on-the-fly subkey computation for encryption and decryption. However, it is not possible to produce the first decryption subkey directly from the original key in a single computation. Therefore, prior to the first decryption with a specific key, Rijndael requires a one-time execution of the key schedule, cycling through all the subkeys, to generate the first decryption subkey. Thereafter, all other decryption subkeys can be recomputed on-the-fly. This places a slight resource burden on Rijndael’s key agility.

Serpent supports on-the-fly subkey computation for encryption and decryption. For decryption, only a single computation is needed to obtain the first decryption subkey from the original key. This computation is distinct from the transformation that is used for every other subkey. The need to prepare for the decryption process by computing the first subkey by using a distinct transform places a slight resource burden on the key agility of this algorithm.

Twofish supports on-the-fly subkey computation in any order. As a result, this algorithm is very key agile.

Although subkey computation is discussed in this section because it is a general property of algorithms, different modes of subkey computation are mainly relevant in restricted-memory environments (see Sec. 3.5). In restricted-memory environments, on-the-fly subkey computation is beneficial because memory, which is at a premium, is not needed to store the pre-computed subkeys. With on-the-fly subkey computation, subkeys are generated as they are needed and then discarded. Therefore, with respect to key setup, Twofish, Serpent, and Rijndael are well suited to memory-restricted environments, followed to a lesser degree by MARS and RC6.

### 3.9 Other Versatility and Flexibility

#### 3.9.1 Parameter Flexibility

In the call for candidate algorithms [32], NIST indicated that, at a minimum, all AES candidates must support a 128 bit block size and three key sizes of 128 bits, 192 bits, and 256 bits. The call for algorithms also indicated that an algorithm’s support for additional key and block sizes would be considered as part of the evaluation. Another potential area for flexibility that was not identified in Ref. [32] is the number of rounds to be used for the encryption and decryption operations. Algorithm flexibility may be beneficial in the future if NIST determines that changes to the algorithm are necessary due to new attacks or requirements. However, before any of these additional key, block, or round sizes could be specified for use in the AES, they would need to undergo further analysis.

Below is a summary of the key, block, and round size capabilities that each of the algorithms offers (i.e., capabilities that are integrated into the current algorithm specifications).

MARS: MARS supports key sizes from 128 bits to 448 bits, in multiples of 32 bits. Three possible approaches to extending the design of MARS to 256 bit blocks are mentioned in the MARS specification.

RC6: The RC6 specification asserts that RC6 is “a fully-parameterized family of encryption algorithms.” Members of the family are specified by RC6-*w/r/b*, where *w* is the word size in bits, *r* is the number of rounds, and *b* is the key size in bytes.

Rijndael: Rijndael supports block sizes and key sizes of 128 bits, 192 bits, and 256 bits, in any combination. The Rijndael specification only defines the number of rounds and the offsets for the Shiftrow function for combinations of key sizes and block sizes within this range. Otherwise, in principle, any combination of key sizes and block sizes that are multiples of 32 bits could be accommodated. The number of rounds of Rijndael is fixed but, if necessary, can be modified as a parameter.

Serpent: Serpent can handle any key size up to 256 bits, due to the padding scheme used in key setup. The Serpent specification asserts that versions of Serpent with different block sizes (64 bits, 256 bits, or 512 bits, for example) would be straightforward to develop.

Twofish: The Twofish algorithm can use any key size up to 256 bits. Twofish is defined for the AES key sizes; keys of other sizes can be padded with zeroes to reach the next AES defined key size. Additionally, the Twofish specification indicates that if extra rounds are required, the key schedule is designed to allow for natural extensions.

RC6 and Rijndael offer the greatest flexibility in supporting key, block, and round sizes beyond the required values. MARS, Serpent, and Twofish provide for key sizes other than 128 bits, 192 bits, and 256 bits, but less flexibility is provided in block and round sizes.

#### 3.9.2 Implementation Flexibility

Another aspect of flexibility is the possibility of optimizing cipher elements for particular environments. One example of implementation flexibility is on-the-fly subkey computation, discussed in Sec. 3.8; another example is pipelining for hardware implementations, discussed in Sec. 3.5. Some of the algorithm specifications identify other elements that can be implemented in alternative ways, providing the flexibility to optimize implementations for different performance goals. Such elements are summarized in this section.

One category of flexibility that, to some extent, is common to all of the finalists is the potential for resource sharing in hardware. Any element of an algorithm that is used repeatedly typically offers tradeoffs in hardware: to maximize the throughput, the element is duplicated, and to minimize the chip area, the resource is shared. Resource sharing between encryption and decryption is discussed in Sec. 3.5; other types of resource sharing are summarized below for each finalist.

MARS: The MARS S-boxes are used in all four types of MARS rounds, as well as the key schedule; thus, logic for the S-boxes could be shared in hardware implementations. The four types of round functions also share other operations, and they have a similar overall structure.

RC6: The quadratic function in RC6 can be implemented via a squaring operation rather than a general multiplication operation. In hardware, resource sharing is possible in the two halves of the round function, in particular, for the squaring/multiplication and variable rotation operations. The key schedule might also potentially share the resources for variable rotations.

Rijndael: At the cost of an extra transformation in the key schedule, decryption can be implemented with the same structure as encryption, although with different components. To optimize for throughput on processors with a word length of 32 bits or more, the different steps of the round function can be combined into a set of four table lookups. A single table can replace the lookup tables, at the cost of additional rotation operations. The same optimization applies to the decryption round function, although with a different table. The MixColumn operation is designed to allow an efficient implementation on 8 bit processors. Multiplication in the Galois field can be implemented either with Boolean operations and fixed rotations or with a table. Rijndael’s key schedule uses its S-box, offering an opportunity for resource sharing in hardware implementations.

Serpent: The Serpent specification describes a “bitslice” implementation that can optimize the calculation of the S-boxes. Each round contains 32 parallel applications of one of the eight 4×4 S-boxes. The bitslice implementation simultaneously calculates all of the applications of the S-box by applying appropriate sequences of Boolean operations to four 32 bit words. One sequence of Boolean operations is required for each of the four output bits. For 32 bit processors, the bitslice method is faster than looking up S-box values in stored tables, during which the processor is mostly idle as it computes operations on the 4 bit words. The increase in speed comes at a slight cost in memory, because the S-box tables are likely to require less memory than any equivalent sequences of Boolean operations. Serpent’s key schedule uses the S-boxes, offering an opportunity for resource sharing in hardware implementations.

Twofish: The Twofish specification describes four options for implementing the key dependent S-boxes, plus a fifth option that applies only to implementations that are coded in assembly language. The options represent a good range of the possible tradeoffs for setup versus throughput. For example, the “full keying” option gives the greatest throughput by pre-computing the combination of each of the four S-boxes with the appropriate column of the maximum distance separable (MDS) matrix. On the other end of the spectrum, the “zero keying” option uses the minimum setup time and table storage by computing the S-boxes on-the-fly from key material and two fixed 8 bit permutations. In order to save additional ROM on smart cards, the 8 bit permutations can be computed from the underlying 4 bit permutations, although at a heavy cost in throughput.

In hardware, the analogous construction of the 8 bit permutations of Twofish from its constituent 4 bit permutations would decrease the number of gates. The modular nature of other Twofish elements offers several possibilities for resource sharing. For example, the structures of the key schedule and the S-boxes are similar; similarly, each round contains two sets of S-box computations. As in software, another means of increasing throughput, at the cost of setup time, would be to pre-compute the S-boxes, which could then be stored in on-chip RAM.

### 3.10 Potential for Instruction-Level Parallelism

It is anticipated that future processors will support various modes of parallelism to a greater extent than existing processors. This raises the following type of question. If an unlimited number of instruction issue slots are available so that any potential parallelism for single block encryption in a finalist can theoretically be exploited, to what extent can the finalist take advantage of this situation? (Note: The issue of encrypting multiple blocks simultaneously using interleaving was addressed in Sec. 3.3.6.)

Some information can be gleaned from an examination of the operations to be executed for an algorithm. One concept, in this regard, is that of a critical path through code for a particular instruction set [18]: each instruction can be weighted according to the number of latent clock cycles. Latent clock cycles refer to the number of cycles between the instruction issuance and the availability of the result to another instruction. A critical path could then be defined to be the path from plaintext to ciphertext requiring the largest number of cycles. Table 15 presents the results of several studies.

The studies discussed in Refs. [18] and [45] present two theoretical estimates that indicate quite similar lower bounds for the critical paths of the finalists. See the “Crit1” and “Crit2” columns of Table 15.

The author of Ref. [18] extended his analysis by measuring the performance of compiled C code when using one through eight instruction issue slots of a hypothetical family of VLIW^7^ processors. The theoretical performance results in Ref. [18] for the five instruction issue slot case were later confirmed when the author measured the throughput of the algorithms (in feedback mode) on an actual VLIW processor with five instruction issue slots [19].

Two independent studies (see Refs. [18] and [98]) also provided comparable estimates of instruction-level parallelism in software. The results of these studies are also summarized in Table 15.

In conclusion, Rijndael has the most potential to benefit from instruction-level parallelism. The rest of the algorithms appear to have less opportunity to benefit from instruction-level parallelism.

## 4. Intellectual Property Issues

During the initial call for algorithms to develop the AES, NIST established a goal of worldwide, royalty free availability for the AES. While it is impossible to guarantee such an outcome, NIST did take specific steps to help achieve this goal. First, NIST required that the algorithm submitters identify all known intellectual property (IP) that may be infringed by implementing their candidate algorithm. Secondly, submitters had to obtain and provide written agreements as to the royalty free availability and use of all the previously identified IP, in the event that their candidate was selected for inclusion in the AES. NIST also specifically called attention to the IP issue at its various AES conferences and in public calls for IP related comments. In its call for Round 2 public comments, NIST stated:

NIST seeks detailed comments regarding any intellectual property—particularly any patent not already identified by the finalists’ submitters—that may be infringed by the practice of any of the finalist algorithms. This also includes comments from all parties—including submitters—regarding specific claims that the practice of a finalist algorithm infringes on their patent(s). Claims regarding infringement of copyrighted software are also particularly solicited. NIST views this input as a critical factor in the eventual widespread adoption and implementation of the algorithm(s) specified in the FIPS.

NIST reminds all interested parties that the adoption of AES is being conducted as an open standards-setting activity. Specifically, NIST has requested that all interested parties identify to NIST any patents or inventions that may be required for the use of AES. NIST hereby gives public notice that it may seek redress under the antitrust laws of the United States against any party in the future who might seek to exercise patent rights against any user of AES that have not been disclosed to NIST in response to this request for information. [34]

After comments were analyzed, and the review process was completed, IP was not a factor in NIST’s selection of the proposed AES algorithm. Consistent with its practice for FIPS, however, NIST intends to state in the proposed AES FIPS that U.S. and foreign patents may cover cryptographic devices implementing the standard.

## 5. Finalist Profiles

The following summarizes the salient information that NIST was able to accrue about the finalists.

### 5.1 MARS

#### 5.1.1 General Security

MARS has no known security attacks.

Unlike the other finalists, MARS uses both data dependent rotations and S-boxes as non-linear components. The nonstandard, heterogeneous round structure (16 mixing and 16 core rounds) of MARS makes it difficult to assess the margin of security provided, as discussed in Sec. 3.2.2. However, MARS appears to have a high margin of security. MARS has received some criticism on its complexity, which may have hindered its security analysis during the timeframe of the AES development process. Refer to Sec. 3.2 for details.

#### 5.1.2 Software Implementations

The efficiency of software implementations of MARS depends on how well the processor/language combination handles the 32 bit multiplication and variable rotation operations. This causes some variation between processors in the same family, and also causes some variation between compilers on a given processor. MARS tends to finish in the middle range in overall performance for encryption/decryption and for key setup, as shown in Table 4 of Sec. 3.3.4. Refer to Sec. 3.3 for details.

#### 5.1.3 Restricted-Space Environments

MARS is not well suited for restricted-space environments because of its ROM requirement. As indicated by Table 6, the ROM requirement tends to be high. MARS has a disadvantage in that it uses pattern-matching operations during key setup, requiring extra resources. Refer to Sec. 3.4 for details.

#### 5.1.4 Hardware Implementations

MARS has an above average area requirement. Its throughput is generally below average, and its efficiency is uniformly below average. The speed of a MARS implementation is independent of the key size used. Refer to Sec. 3.5 for details.

#### 5.1.5 Attacks on Implementations

When implemented on devices that are vulnerable to timing or power attacks, MARS is difficult to defend, due to the use of multiplications, variable rotations, and additions. The use of masking techniques to provide MARS with some defense against these attacks produces severe performance degradation and high ROM requirements, as indicated by Table 14. The key schedule is slightly vulnerable to the power analysis attacks in Refs. [13] and [16]. Refer to Sec. 3.6 for details.

#### 5.1.6 Encryption vs Decryption

Encryption and decryption in MARS are similar functions. Thus, the speed of MARS does not vary significantly between encryption and decryption. One FPGA study reports that the implementation of both encryption and decryption takes about 10 % more space than the implementation of encryption alone. Refer to Secs. 3.5.3.3 and 3.7 for details.

#### 5.1.7 Key Agility

MARS requires the computation of 10 of the 40 subkeys at a time, requiring additional resources to store these 10 subkeys. This is a detriment in memory-restricted environments. MARS also requires a one-time execution of the key schedule to generate all subkeys prior to the first decryption with a specific key. The computation of multiple subkeys at one time uses more memory resources than are required for algorithms performing on-the-fly subkey computation of a single subkey. Refer to Sec. 3.8 for details.

#### 5.1.8 Other Versatility and Flexibility

MARS supports key sizes from 128 bits to 448 bits. Refer to Sec. 3.9 for details.

#### 5.1.9 Potential for Instruction-Level Parallelism

As shown by Table 15, MARS has a limited potential for parallelism for a single block encryption. Refer to Sec. 3.10 for details.

### 5.2 RC6

#### 5.2.1 General Security

RC6 has no known security attacks.

RC6 uses data dependent rotations as non-linear components. Its security margin appears to be adequate. RC6 has received praise for its simplicity, which may aid in its security analysis during the timeframe of the AES development process. RC6’s lineage is a plus: its predecessor, RC5, has been subjected to previous analysis. Refer to Sec. 3.2 for details.

#### 5.2.2 Software Implementations

The predominant operations for RC6 are multiplications and variable rotations. Software performance depends on how well the processor/language combination handles these operations. The behavior of RC6 for encryption and decryption is mixed, as shown by Tables 16–25 of Appendix A. Generally, RC6 is the fastest finalist on 32 bit platforms. However, its relative performance drops off substantially on 64 bit processors. RC6’s performance improves most significantly relative to the other finalists if it is used in a mode capable of interleaving. Key setup time is average. Refer to Sec. 3.3 for details.

#### 5.2.3 Restricted-Space Environments

RC6 has a low ROM requirement, which is an advantage in space-restricted environments. However, RC6 lacks an on-the-fly subkey computation capability for decryption, which creates a high RAM requirement. It is, therefore, not well suited for implementation on devices with a very restricted amount of RAM available when decryption is required. Refer to Sec. 3.4 for details.

#### 5.2.4 Hardware Implementations

RC6 can be compactly implemented. Its throughput is average, and it is faster in non-feedback modes. The throughput of an RC6 implementation is independent of the key size used. Refer to Sec. 3.5 for details.

#### 5.2.5 Attacks on Implementations

When implemented on devices vulnerable to timing or power attacks, RC6 is difficult to defend, due to the use of multiplications, variable rotations, and additions. The use of masking techniques to provide RC6 with some defense against these attacks degrades its performance considerably, as indicated by Table 14. However, when masked, the RAM and ROM requirements are very reasonable when compared to other finalists. RC6 is slightly vulnerable to the power analysis attacks in Refs. [13] and [16]. Refer to Sec. 3.6 for details.

#### 5.2.6 Encryption vs Decryption

Encryption and decryption in RC6 are similar functions. Thus, the efficiency of RC6 does not vary significantly between encryption and decryption. One FPGA study reports that the implementation of both encryption and decryption takes only about 10 % more space than the implementation of encryption alone. Refer to Secs. 3.5.3.3 and 3.7 for details.

#### 5.2.7 Key Agility

RC6 supports on-the-fly subkey computation only for encryption, given about 100 bytes of intermediate values. The decryption subkeys must be pre-computed. The lack of on-the-fly subkey computation reduces the level of key agility afforded by RC6. Refer to Sec. 3.8 for details.

#### 5.2.8 Other Versatility and Flexibility

Block, key, and round sizes are parameterized. RC6 supports key sizes much higher than 256 bits. Refer to Sec. 3.9 for details.

#### 5.2.9 Potential for Instruction-Level Parallelism

As shown by Table 15, RC6 has a limited potential for parallelism for a single block encryption. Refer to Sec. 3.10 for details.

### 5.3 Rijndael

#### 5.3.1 General Security

Rijndael has no known security attacks.

Rijndael uses S-boxes as non-linear components. Rijndael appears to have an adequate security margin, but has received some criticism suggesting that its mathematical structure may lead to attacks. On the other hand, the simple structure may have facilitated its security analysis during the timeframe of the AES development process. Refer to Sec. 3.2 for details.

#### 5.3.2 Software Implementations

As shown by Tables 16–25 of Appendix A, Rijndael performs encryption and decryption very well across a variety of platforms, including 8 bit and 64 bit platforms, and DSPs. However, there is a decrease in performance with the higher key sizes because of the increased number of rounds that are performed. Rijndael’s high inherent parallelism facilitates the efficient use of processor resources, resulting in very good software performance even when implemented in a mode not capable of interleaving. Rijndael’s key setup time is fast. Refer to Sec. 3.3 for details.

#### 5.3.3 Restricted-Space Environments

In general, Rijndael is very well suited for restricted-space environments where either encryption or decryption is implemented (but not both), as indicated by Tables 5 and 6. It has very low RAM and ROM requirements. A drawback is that ROM requirements will increase if both encryption and decryption are implemented simultaneously, although it appears to remain suitable for these environments. The key schedule for decryption is separate from encryption. Refer to Sec. 3.4 for details.

#### 5.3.4 Hardware Implementations

Rijndael has the highest throughput of any of the finalists for feedback modes and second highest for non-feedback modes. For the 192 bit and 256 bit key sizes, throughput falls in standard and unrolled implementations because of the additional number of rounds. For fully pipelined implementations, the area requirement increases, but the throughput is unaffected. Refer to Sec. 3.5 for details.

#### 5.3.5 Attacks on Implementations

The operations used by Rijndael are among the easiest to defend against power and timing attacks. The use of masking techniques to provide Rijndael with some defense against these attacks does not cause significant performance degradation relative to the other finalists, and its RAM requirement remains reasonable, as indicated by Table 14. Rijndael appears to gain a major speed advantage over its competitors when such protections are considered. However, implementations are vulnerable to the power analysis attack in Ref. [16], and slightly vulnerable to the power analysis attack in Ref. [13]. Refer to Sec. 3.6 for details.

#### 5.3.6 Encryption vs Decryption

The encryption and decryption functions in Rijndael differ. One FPGA study reports that the implementation of both encryption and decryption takes about 60 % more space than the implementation of encryption alone. Rijndael’s speed does not vary significantly between encryption and decryption, although the key setup performance is slower for decryption than for encryption. Refer to Secs. 3.5.3.3 and 3.7 for details.

#### 5.3.7 Key Agility

Rijndael supports on-the-fly subkey computation for encryption. Rijndael requires a one-time execution of the key schedule to generate all subkeys prior to the first decryption with a specific key. This places a slight resource burden on the key agility of Rijndael. Refer to Sec. 3.8 for details.

#### 5.3.8 Other Versatility and Flexibility

Rijndael fully supports block sizes and key sizes of 128 bits, 192 bits and 256 bits, in any combination. In principle, the Rijndael structure can accommodate any block sizes and key sizes that are multiples of 32, as well as changes in the number of rounds that are specified. Refer to Sec. 3.9 for details.

#### 5.3.9 Potential for Instruction-Level Parallelism

As indicated by Table 15, Rijndael has an excellent potential for parallelism for a single block encryption. Refer to Sec. 3.10 for details.

### 5.4 Serpent

#### 5.4.1 General Security

Serpent has no known security attacks.

Serpent uses S-boxes as non-linear components. Serpent appears to have a high security margin and a simple structure, which may have facilitated its security analysis during the timeframe of the AES development process. Refer to Sec. 3.2 for details.

#### 5.4.2 Software Implementations

Serpent is generally the slowest of the finalists in software speed for encryption and decryption, as indicated by Tables 16–25 of Appendix A. Serpent’s key setup time is average. Refer to Sec. 3.3 for details.

#### 5.4.3 Restricted-Space Environments

Serpent is well suited to restricted-space environments, as indicated by Table 6, due to low RAM and ROM requirements. A drawback is that ROM requirements will increase if both encryption and decryption are implemented simultaneously, but Serpent should remain suitable for restricted-space environments. Refer to Sec. 3.4 for details.

#### 5.4.4 Hardware Implementations

Fully pipelined implementations of Serpent offer the highest throughput of any of the finalists for non-feedback modes. Serpent is second in feedback mode throughput for the basic architecture and fully unrolled implementations. Efficiency is generally very good, and Serpent’s speed is independent of key size. Refer to Sec. 3.5 for details.

#### 5.4.5 Attacks on Implementations

The operations used by Serpent are among the easiest to defend against timing and power attacks. Serpent is most effectively defended against these attacks by using the bitslice mode. Table 14 shows that when bitsliced Serpent is defended against power attacks by masking, its performance degrades somewhat. In particular, Serpent’s speed is low, and its ROM requirement is high. Implementations are vulnerable to the power analysis attack in Ref. [16]. Refer to Sec. 3.6 for details.

#### 5.4.6 Encryption vs Decryption

Encryption and decryption in Serpent are different functions that share very limited resources in hardware. One FPGA study reports that the implementation of both encryption and decryption takes roughly twice the amount of space needed for encryption alone. This is a disadvantage in hardware when both functions need to be implemented. Serpent’s speed does not vary significantly between encryption and decryption. Refer to Secs. 3.5.3.3 and 3.7 for details.

#### 5.4.7 Key Agility

Serpent supports on-the-fly subkey computation for encryption and decryption. For decryption, only a single computation is needed to obtain the first decryption subkey from the original key. This computation is distinct from the transformation that is used for every other subkey. This places a slight resource burden on the key agility of Serpent. Refer to Sec. 3.8 for details.

#### 5.4.8 Other Versatility and Flexibility

Serpent can handle any key sizes up to 256 bits. In addition, a bitslice technique may be used on 32 bit processors to improve performance. Refer to Sec. 3.9 for details.

#### 5.4.9 Potential for Instruction Level Parallelism

As indicated by Table 15, Serpent has a limited potential for parallelism for a single block encryption. Refer to Sec. 3.10 for details.

### 5.5 Twofish

#### 5.5.1 General Security

Twofish has no known security attacks.

Twofish uses S-boxes as non-linear components. Twofish appears to have a high security margin, but has been criticized because of its key separation property and for its complexity, which may have hindered its security analysis during the timeframe of the AES development process. Refer to Sec. 3.2 for details.

#### 5.5.2 Software Implementations

As shown by Tables 16–25 of Appendix A, Twofish has mixed results when performing encryption and decryption. Key setup time is slow. Encryption/decryption or key setup performance decreases with the larger key sizes, depending upon the keying option used. Refer to Sec. 3.3 for details.

#### 5.5.3 Restricted-Space Environments

As shown by Table 6, the RAM and ROM requirements of Twofish appear to make it suitable for restricted-space environments. Refer to Sec. 3.4 for details.

#### 5.5.4 Hardware Implementations

Throughput and efficiency in the basic architecture and the pipelined and unrolled implementations are generally average. Twofish’s throughput is reduced somewhat for the larger key sizes in both the standard architecture and pipelined implementations. Compact implementations are possible. Refer to Sec. 3.5 for details.

#### 5.5.5 Attacks on Implementations

Twofish uses addition, which is somewhat difficult to defend against timing and power analysis attacks. The use of masking techniques to provide Twofish with some defense against these attacks does not degrade performance considerably, although its RAM usage increases significantly, as indicated by Table 14. Twofish is vulnerable to the power analysis attack in Ref. [16]. Refer to Sec. 3.6 for details.

#### 5.5.6 Encryption vs Decryption

Encryption and decryption in Twofish are nearly identical functions. Thus, the efficiency of Twofish does not vary significantly between encryption and decryption. One FPGA study reports that the implementation of both encryption and decryption takes only about 10 % more space than the implementation of encryption alone. Refer to Secs. 3.5.3.3 and 3.7 for details.

#### 5.5.7 Key Agility

Twofish supports on-the-fly subkey computation for both encryption and decryption. As a result, Twofish is very key agile. Refer to Sec. 3.8 for details.

#### 5.5.8 Other Versatility and Flexibility

The Twofish specification describes four options for implementing the key dependent S-boxes, allowing various performance tradeoffs. Twofish supports arbitrary key sizes up to 256 bits. Refer to Sec. 3.9 for details.

#### 5.5.9 Potential for Instruction Level Parallelism

As indicated by Table 15, Twofish has a limited potential for parallelism for a single block encryption. Refer to Sec. 3.10 for details.

## 6. Summary Assessments of the Finalists

The following are NIST’s overall assessments of the candidates, summarizing the results of the Round 2 analysis. The assessments below are not intended to be a comprehensive list or description of the features and properties of the algorithms, which were discussed in the preceding sections of this report and in the public comments and analyses. The preceding sections in this report should be consulted for the specific details of each algorithm.

As discussed in Sec. 1.3, security was considered of primary importance during NIST’s evaluation of the finalists. Since many of the remaining (non-security) analysis results often covered both the “cost” and “algorithm characteristics” evaluation criteria, NIST considered all of these factors together, as secondary to security (i.e., “cost” factors could not be clearly separated from “algorithm characteristics” factors.).

As indicated above in Sec. 4, although Intellectual Property (IP) information was reviewed during the evaluation, it did not play a role in distinguishing one algorithm from another, and therefore is not addressed in this section.

### 6.1 General Security

Based on the security analysis performed to-date, there are no known security attacks on any of the five finalists, and *all five algorithms appear to have adequate security for the AES.* In terms of security margin, MARS, Serpent, and Twofish appear to have high security margins, while the margins for RC6 and Rijndael appear adequate. Some comments criticized Rijndael for its mathematical structure and Twofish for its key separation property; however, those observations have not led to attacks.

### 6.2 Software Implementations

RC6 and Rijndael generally demonstrate above average encryption and decryption speed for 128 bit keys, with RC6 doing particularly well on 32 bit platforms and Rijndael performing more evenly across platforms. MARS has average performance for encryption and decryption across platforms, depending on how well the processor performs 32 bit multiplications and variable rotations. Twofish has mixed results across platforms for encryption and decryption, but is generally an average performer among the finalists. Serpent is generally the slowest of the finalists for encryption and decryption.

The key setup performance for Rijndael is consistently the fastest of all the finalists. The key setup performance for MARS, RC6, and Serpent is average, while that of Twofish is consistently the slowest of the finalists.

MARS, RC6, and Serpent exhibit consistent encryption and decryption performance for all three key sizes. Rijndael’s performance for encryption and decryption decreases with higher key sizes due to the increased number of rounds. The encryption/decryption or key setup performance of Twofish decreases with the larger key sizes, depending on which keying option is used.

The performance of RC6 improves most significantly relative to the other finalists if it is used in a mode capable of interleaving.

### 6.3 Restricted-Space Environments

Rijndael has very low RAM and ROM requirements and is very well suited to restricted-space environments when either encryption or decryption is implemented. A drawback is that its ROM requirements increase if both encryption and decryption are implemented simultaneously, although Rijndael still appears to remain suitable for restricted-space environments.

Serpent has low RAM and ROM requirements and is well suited to restricted-space environments, when either encryption or decryption is implemented. As with Rijndael, Serpent’s ROM requirements increase when both encryption and decryption are implemented simultaneously, but the algorithm appears to remain suitable for restricted-space environments.

The RAM and ROM requirements of Twofish appear to make it suitable for restricted-space environments.

RC6 has a low ROM requirement, which is an advantage in restricted-space environments. However, the algorithm lacks an on-the-fly subkey computation capability for decryption, creating a high RAM requirement relative to the other finalists. Therefore, RC6 is not well suited for implementation on devices with a very restricted amount of available RAM when decryption is required.

MARS is not well suited for restricted-space environments due to its ROM requirement, which tends to be the highest among the finalists. Additionally, MARS’ key schedule involves pattern-matching operations, which requires extra resources.

### 6.4 Hardware Implementations

Serpent and Rijndael appear to have the best hardware throughput of the finalists, for both feedback and non-feedback modes. Serpent offers the highest throughput of the finalists in non-feedback modes, and its efficiency (throughput/area) is generally very good. Rijndael offers the highest throughput among the finalists for feedback modes. For the larger key sizes, Rijndael’s throughput decreases, and fully pipelined implementations require a higher area but without a decrease in throughput. Rijndael’s efficiency also appears to be generally very good.

RC6 and Twofish generally appear to have average throughput, and both algorithms can be compactly implemented. RC6’s throughput increases in non-feedback modes. Twofish’s throughput is somewhat reduced for the larger key sizes.

MARS has an above average area requirement, its throughput is generally below average, and its efficiency is uniformly below average.

### 6.5 Attacks on Implementations

Rijndael and Serpent use operations that are among the easiest to defend against power and timing attacks. Since it uses the addition operation, Twofish is somewhat more difficult to defend against those same attacks. RC6 and MARS are the most difficult of the five finalists to defend against timing and power attacks, due to their use of multiplications, variable rotations, and additions.

When the algorithms are implemented using masking techniques for defense against power and timing attacks, it appears that the speed of Rijndael, Serpent, and Twofish are impacted significantly less than that of MARS and RC6. Rijndael also appears to gain a major speed advantage over the other algorithms when such protections are considered.

### 6.6 Encryption vs Decryption

Twofish, MARS, and RC6 need very little additional area to implement both encryption and decryption in hardware, as opposed to implementing encryption alone. The encryption and decryption functions are nearly identical for Twofish, while the functions are similar for both MARS and RC6.

Rijndael’s encryption and decryption are different—more than for Twofish, MARS, and RC6—although Rijndael can be implemented in such a way as to share some hardware resources.

For Serpent, encryption and decryption are different functions that appear to share only very limited hardware resources.

All of the finalists show very little speed variation, if any, between encryption and decryption functions for a given key size. Rijndael’s key setup performance is slower for decryption than for encryption.

### 6.7 Key Agility

Twofish supports on-the-fly subkey computation for both encryption and decryption. Serpent also supports on-the-fly subkey computation for both encryption and decryption; however, the decryption process requires one additional computation. Rijndael supports on-the-fly subkey computation for encryption, but requires a one-time execution of the entire key schedule prior to the first decryption with a particular key. MARS has characteristics that are similar to Rijndael’s, except that 10 subkeys must be computed and stored at a time. This places an additional resource burden on a MARS implementation. RC6 supports on-the-fly subkey computation for encryption only, given intermediate values. Decryption subkeys for RC6 must be precomputed.

### 6.8 Other Versatility and Flexibility

MARS supports key sizes ranging from 128 bits to 448 bits.

RC6 has parameterized block, key, and round sizes, including support for key sizes much larger than 256 bits.

Rijndael supports additional block and key sizes in increments of 32 bits, and the number of rounds can be altered.

Serpent supports any key size up to 256 bits, and the bitslice implementation can improve its performance on many processors.

Twofish supports arbitrary key sizes up to 256 bits, and the algorithm specification offers four options that allow various performance tradeoffs.

### 6.9 Potential for Instruction-Level Parallelism

Rijndael has the most potential to benefit from instruction-level parallelism. The rest of the algorithms appear to have less opportunity to benefit from instruction-level parallelism.

## 7. Conclusion

Each of the finalist algorithms appears to offer adequate security, and each offers a considerable number of advantages. Any of the finalists could serve admirably as the AES. However, each algorithm also has one or more areas where it does not fare quite as well as some other algorithm; none of the finalists is outstandingly superior to the rest.

NIST selected Rijndael as the proposed AES algorithm at the end of a very long and complex evaluation process. During the evaluation, NIST analyzed all public comments, papers, verbal comments at conferences, and NIST studies and reports. NIST judged Rijndael to be the best overall algorithm for the AES.

Rijndael appears to be consistently a very good performer in both hardware and software across a wide range of computing environments regardless of its use in feedback or non-feedback modes. Its key setup time is excellent, and its key agility is good. Rijndael’s very low memory requirements make it very well suited for restricted-space environments, in which it also demonstrates excellent performance. Rijndael’s operations are among the easiest to defend against power and timing attacks. Additionally, it appears that some defense can be provided against such attacks without significantly impacting Rijndael’s performance. Rijndael is designed with some flexibility in terms of block and key sizes, and the algorithm can accommodate alterations in the number of rounds, although these features would require further study and are not being considered at this time. Finally, Rijndael’s internal round structure appears to have good potential to benefit from instruction-level parallelism.

There are many unknowns regarding future computing platforms and the wide range of environments in which the AES will be implemented. However, when considered together, Rijndael’s combination of security, performance, efficiency, implementability, and flexibility make it an appropriate selection for the AES for use in the technology of today and in the future.

## 8. Next Steps

Following NIST’s announcement of the proposed AES algorithm, NIST will prepare the draft AES FIPS for public review and comment. After the close of the public comment period, the standard will be revised by NIST, as appropriate, in response to those comments. A review, approval, and promulgation process will then follow. If all steps of the AES development process proceed as planned, it is anticipated that the standard will be completed by the summer of 2001.

When the AES officially becomes a FIPS, a conformance-testing program will be available for AES implementations through NIST’s Cryptographic Module Validation Program (CMVP).

## Figures and Tables

**Fig. 1:**
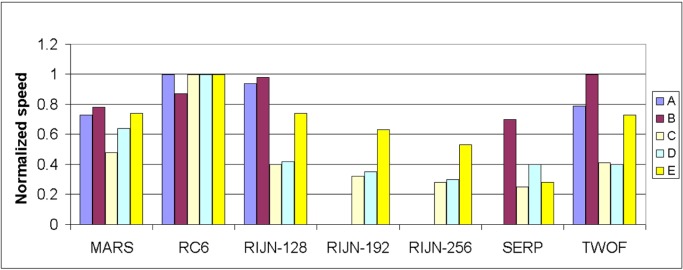
Encryption speed for 32 bit processors (C) from Table 16.

**Fig. 2:**
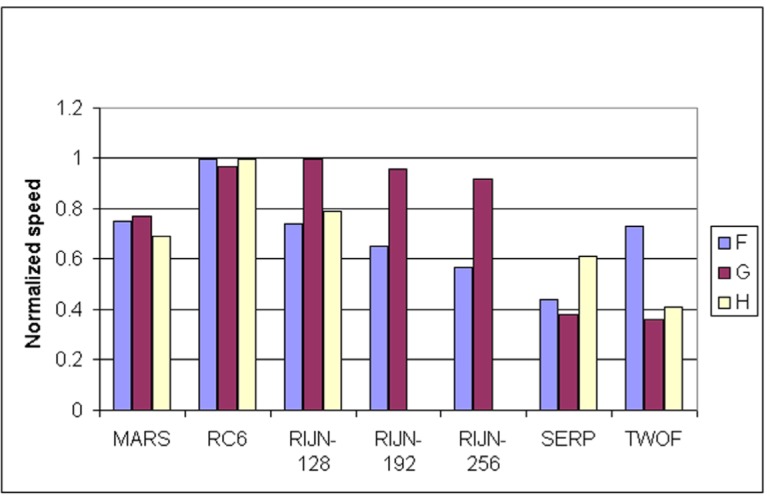
Encryption speed for 32 bit processors (Java) from Table 17.

**Fig. 3:**
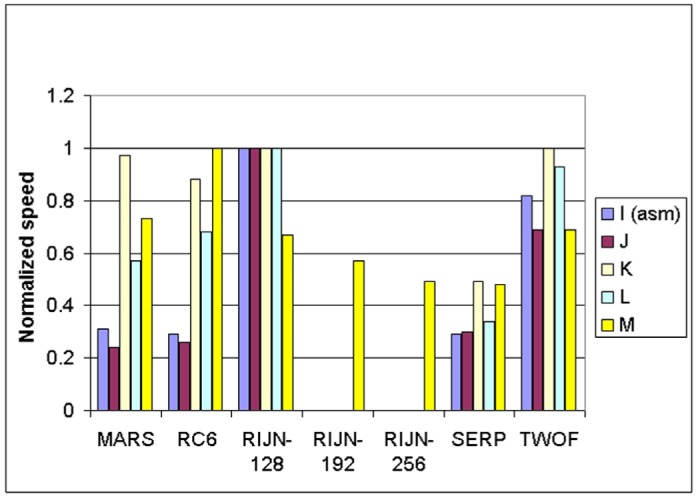
Encryption speed for 64 bit processors (C and assembler) from Table 18.

**Fig. 4:**
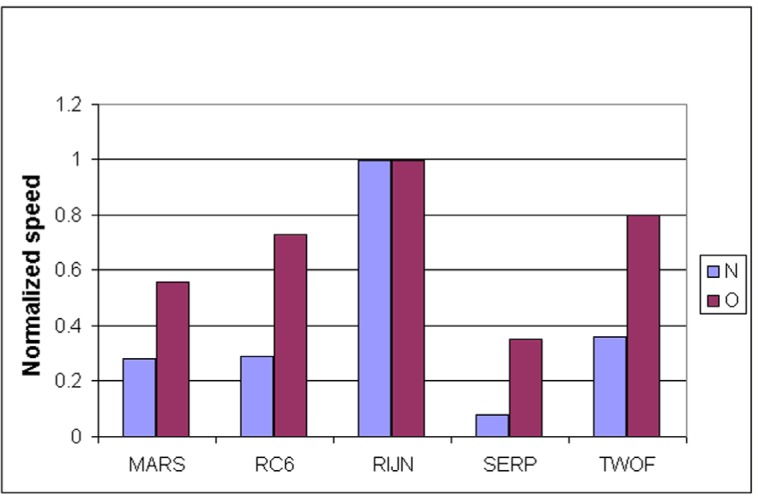
Encryption speed for 8 bit processors from Table 19.

**Fig. 5:**
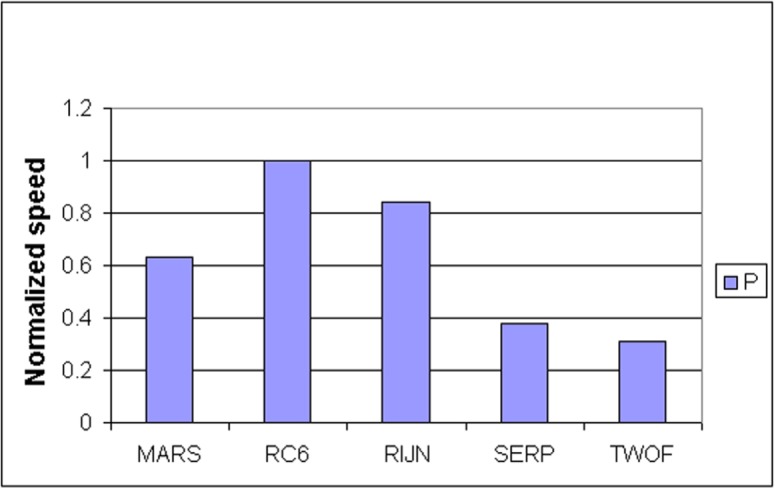
Encryption speed for ARM from Table 20.

**Fig. 6:**
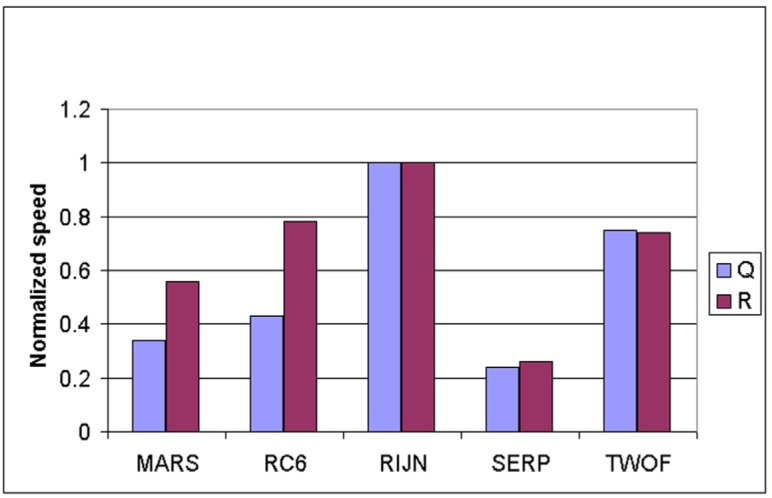
Encryption speed for DSPs from Table 21.

**Fig. 7:**
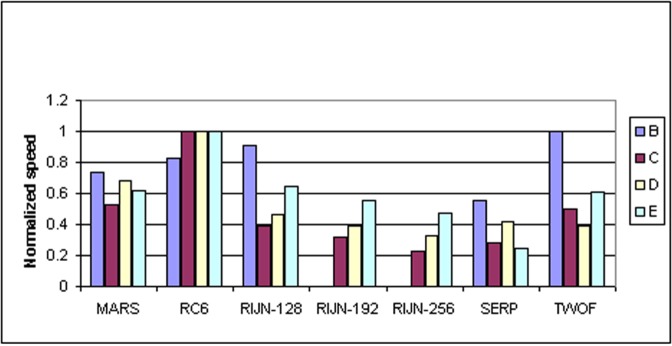
Decryption speed for 32 bit processors (C) from Table 22.

**Fig. 8:**
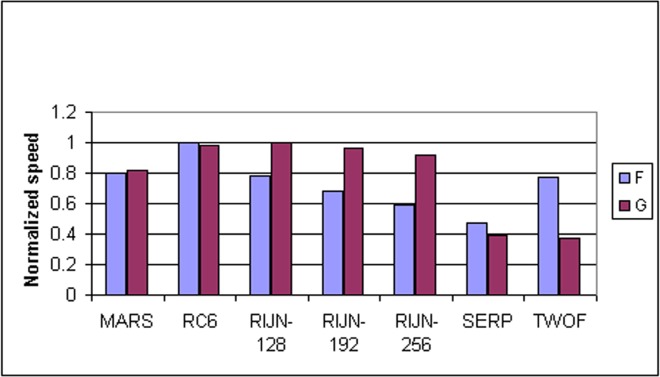
Decryption speed for 32 bit processors (Java) from Table 23.

**Fig. 9:**
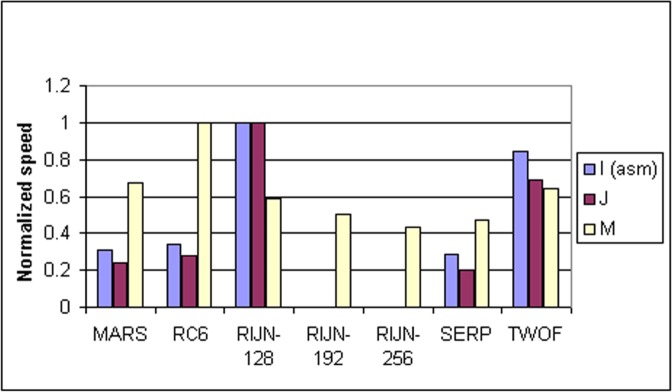
Decryption speed for 64 bit processors (C and assembler) from Table 24.

**Fig. 10:**
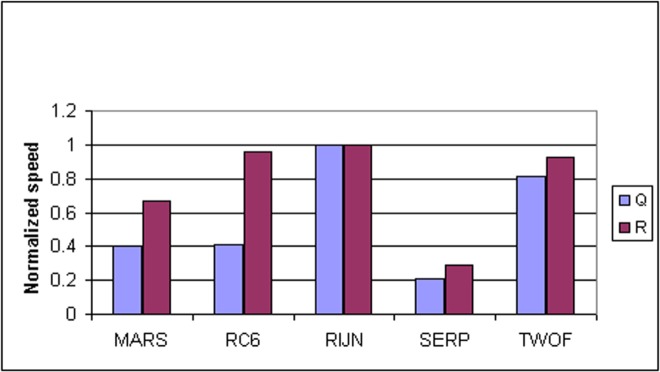
Encryption speed for DSPs from Table 25.

**Fig. 11:**
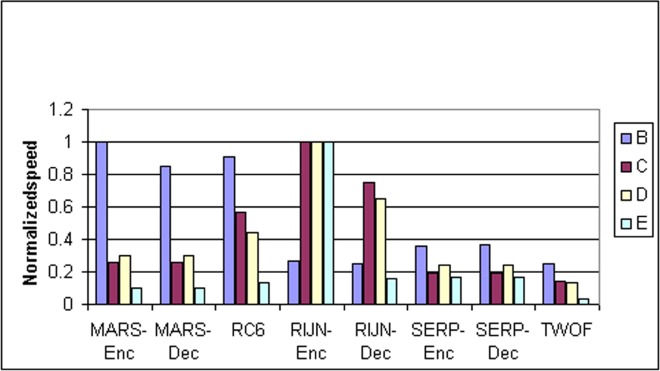
128 bit key setup for 32 bit processors (C) from Table 26.

**Fig. 12:**
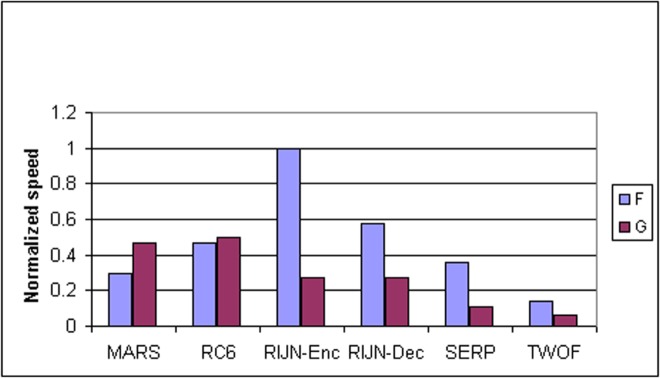
128 bit key setup for 32 bit processors (Java) from Table 27.

**Fig. 13:**
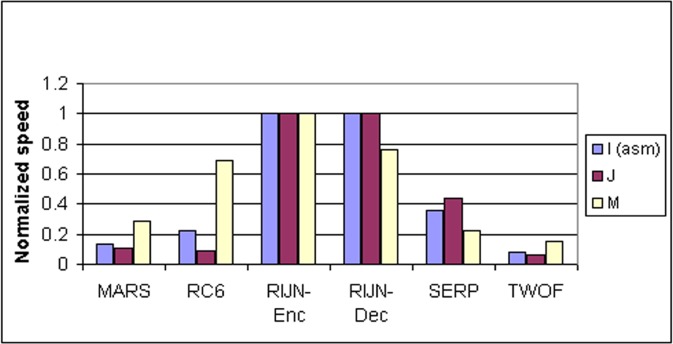
128 bit key setup for 64 bit processors (C and assembler) from Table 28.

**Fig. 14:**
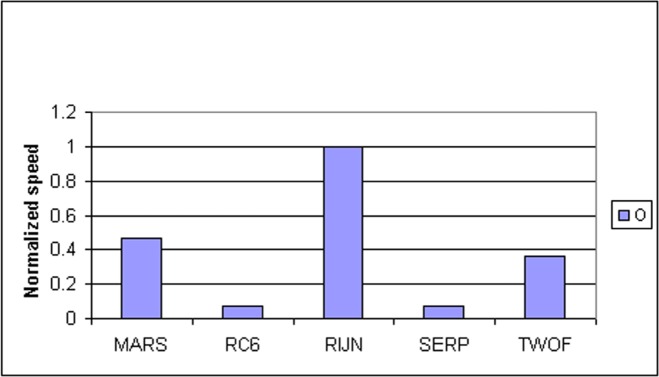
128 bit key setup for an 8 bit processor (C) from Table 29.

**Fig. 15:**
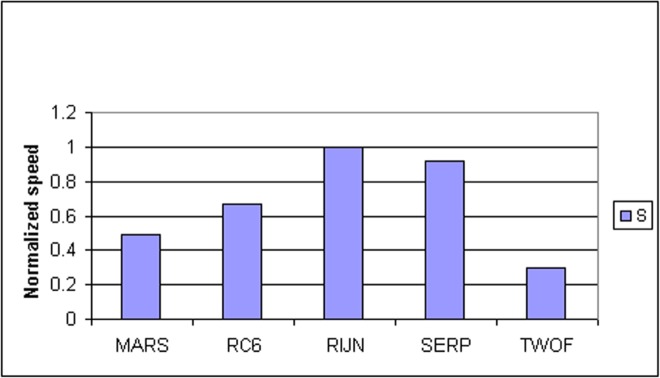
128 bit setup for a DSP from Table 30.

**Table 1:** Summary of reported attacks on reduced-round variants of the finalists

Algorithm, Rounds	Reference	Rounds (Key size)	Type of Attack	Texts	Mem. Bytes	Ops.
MARS	[57]	11C	Amp. Boomerang	2^65^	2^70^	2^229^
16 Core(C)	[58]	16 M, 5 C	Meet-in-Middle	8	2^236^	2^232^
16 Mixing		16 M, 5 C	Diff. M-i-M	2^50^	2^197^	2^247^
(M)		6 M, 6 C	Amp. Boomerang	2^69^	2^73^	2^197^
RC6	[39]	14	Stat. Disting.	2^118^	2^112^	2^122^
20	[60]	12	Stat. Disting.	2^94^	2^42^	2^119^
		14(192,256)	Stat. Disting.	2^110^	2^42^	2^135^
		14(192,256)	Stat. Disting.	2^108^	2^74^	2^160^
		15(256)	Stat. Disting.	2^119^	2^138^	2^215^
Rijndael	[22]	4	Truncated. Diff.	2^9^	small	2^9^
10 (128)		5	Truncated. Diff.	2^11^	small	2^40^
12 (192)		6	Truncated. Diff.	2^32^	7*2^32^	2^72^
14 (256)	[37]	6	Truncated. Diff.	6*2^32^	7*2^32^	2^44^
		7 (192)	Truncated. Diff.	19*2^32^	7*2^32^	2^155^
		7 (256)	Truncated. Diff.	21*2^32^	7*2^32^	2^172^
		7	Truncated. Diff.	2^128^–2^119^	2^61^	2^120^
		8 (256)	Truncated. Diff.	2^128^–2^119^	2^101^	2^204^
		9 (256)	Related Key	2^77^	NA	2^224^
	[63]	7 (192)	Truncated. Diff.	2^32^	7*2^32^	2^184^
		7 (256)	Truncated. Diff.	2^32^	7*2^32^	2^200^
	[40]	7 (192,256)	Truncated. Diff.	2^32^	7*2^32^	2^140^
Serpent	[57]	8 (192,256)	Amp. Boomerang	2^113^	2^119^	2^179^
32	[62]	6 (256)	Meet-in-Middle	512	2^246^	2^247^
		6	Differential	2^83^	2^40^	2^90^
		6	Differential	2^71^	2^75^	2^103^
		6 (192,256)	Differential	2^41^	2^45^	2^163^
		7 (256)	Differential	2^122^	2^126^	2^248^
		8 (192,256)	Boomerang	2^128^	2^133^	2^163^
		8 (192,256)	Amp. Boomerang	2^110^	2^115^	2^175^
		9 (256)	Amp. Boomerang	2^110^	2^212^	2^252^
Twofish	[35]	6 (256)	Impossible Diff.	NA	NA	2^256^
16	[36]	6	Related Key	NA	NA	NA

NA = Information not readily available.

**Table 2:** Encryption and decryption performance by platform

	32 bit (C)	32 bit (Java)	64 bit (C and assembler)	8 bit (C and assembler)	32 bit smartcard (ARM)	Digital Signal Processors
MARS	II	II	II	II	II	II
RC6	I	I	II	II	I	II
Rijndael	II	II	I	I	I	I
Serpent	III	III	III	III	III	III
Twofish	II	III	I	II	III	I

**Table 3:** Key setup performance by platform

	32 bit (C)	32 bit (Java)	64 bit (C and assembler)	8 bit (C and assembler)	Digital Signal Processors
MARS	II	II	III	II	II
RC6	II	II	II	III	II
Rijndael	I	I	I	I	I
Serpent	III	II	II	III	I
Twofish	III	III	III	II	III

**Table 4:** Overall performance

	Enc/Dec	Key setup
MARS	II	II
RC6	I	II
Rijndael	I	I
Serpent	III	II
Twofish	II	III

**Table 5:** A smart card study. Source: Ref. [82], Sec. 3.4.1

	RAM	ROM	ENC	KEY	TIME
MARS	572	5468	45	21	67
RC6	156	1060	34	138	173
RIJN	66	980	25	10	35
SERP	164	3937	71	147	219
TWOF	90	2808	31	28	60

RAM = Total RAM in bytes.

ROM = Total ROM in bytes.

ENC = Time for encryption of one 128 bit block, in units of 1000 cycles.

KEY = Time for key scheduling, in units of 1000 cycles.

TIME = Encryption + key scheduling, in units of 1000 cycles.

**Table 6:** Performance study on the 6805. Source: Ref. [54], Tables 2 and 3

	RAM			ROM		TIME	
	KEYA	ENCA	SCHA	ENCO	EOSO	ENCT	SCHT
MARS	23	91	74	4059	4077	358	213
MARS(2)	160	33	32	3329	4136	34	110
RC6	56	55	38	1342	1374	106	79
RC6(2)	176	24	30	639	933	33	82
RIJN	16	34	0	879	879	9	0
RIJN(d)	16	37	1	976	1049	14	2
SERP	16	85	0	1056	1056	126	0
TWOF	24	36	N/A	N/A	2200	27	2

KEYA = Bytes of RAM needed for subkey storage.

ENCA = Bytes of RAM needed for encryption.

SCHA = Bytes of RAM needed for key setup.

ENCO = Bytes of RAM needed for encryption.

EO+SO = Bytes of RAM needed for encryption + key setup.

ENC = Time to encrypt one block, in units of 1000 cycles.

SCHT = Time to execute key setup, in units of 1000 cycles.

**Table 7:** Operations used by the candidate algorithms. Source: [30], Table 1

MARS	XOR	Table	FixShi	VarShi	Add	Sub	Mul	GFmul
MARS	Y	Y	Y	Y	Y	Y	Y	
RC6	Y		Y	Y	Y		Y	
RIJN	Y	Y	Y					Y
SERP	Y	Y	Y					
TWOF	Y	Y	Y		Y			Y

Y = Yes (operation is used by the algorithm).

Table = Table lookup.

FixShi = Fixed shift or rotate.

VarShi = Data dependent shift or rotate.

Add = Mod 2^32^ addition.

Sub = Mod 2^32^ subtraction.

Mul = Mod 2^32^ multiplication.

Gfmul = GF(2^8^) multiplication.

**Table 8:** An FPGA study of optimized speed for encryption. Source: Ref. [30], Tables 2 and 3

	Non-Feedback			Feedback		
	THRU	AREA	TH/AR	THRU	AREA	TH/AR
RC6	2398	10.8	221	126	3.19	39.6
RIJN	1938	11.0	176	300	5.30	56.6
SERP	4860	9.0	539	444	7.96	55.7
TWOF	1585	9.3	169	120	3.05	39.1

THRU = Throughput in megabits per second.

AREA = Area in units of 1000 CLB slices.

TH/AR = THRU/AREA.

**Table 9:** An FPGA study of the basic architecture for encryption/decryption. Source: Ref. [38], Fig. 10

	THRU	AREA	TH/AR
MARS	39.8	2.7	14.5
RC6	103.9	1.1	91.2
RIJN	331.5	2.9	114.2
SERP	339.4	4.4	76.5
TWOF	177.3	1.0	164.8

THRU = throughput in megabits per second.

AREA = area in units of 1000 CLB slices.

TH/AR = THRU/AREA.

**Table 10:** An FPGA study of the basic architecture for encryption/key setup. Source: Ref. [27], Tables 1–16

	LAT	THRU	TH/AR	KEYA	CORA	AREA	LAT2
MARS	1.96	2.04	2.96	2.28	4.62	6.89	1.45
RC6	0.17	1.12	4.26	0.90	1.74	2.65	0.06
RIJN	0.07	3.53	6.22	1.36	4.31	5.67	0.05
SERP	0.08	1.49	6.62	1.30	1.25	2.55	0.08
TWOF	0.18	1.73	1.84	6.55	2,81	9.36	0.16

LAT = latency in microseconds, using a single system clock.

THRU = throughput in units of 100 megabits per second.

TH/AR = throughput per area, in units of 10 kilobits per (seconds × CLB slices).

KEYA = key-setup circuit area in units of 1000 CLB slices.

CORA = encryption area in units of 1000 CLB slices.

AREA = total area in units of 1000 CLB slices.

LAT 2 = latency in microseconds, using separate system clocks for key setup and encryption.

**Table 11:** An FPGA study of key setup. Source: Ref. [93], Fig. 2

	LAT	BAND	AREA
MARS	270	270	50
RC6	264	264	290
RIJN	36	36	128
SERP	32	4	2060
TWOF	4	4	1260

LAT = latency for subkey generation, in clock cycles.

BAND = number of clock cycles to generate a set of subkeys.

AREA = number of CLB slices required for subkey generation.

**Table 12:** An ASIC study. Source: Ref. [94], Tables 11 and 12

	Basic				Fully Pipelined			
	AR	TH	KE	KD	AR	TH	KE	KD
MARS	127	56	9553	27470	1333	2189	3718	3718
RC6	21	103	8139	8139	554	2171	3660	3660
RIJN	46	443	0	286	471	5163	0	233
SERP	23	202	19	672	438	8030	18	212
TWOF	23	104	61	61	343	2278	0	0

AR = area in units of mm^2^.

TH = throughput, Mbit/s.

KE = key setup time, encrypt, in nanoseconds.

KD = key setup time, decrypt, in nanoseconds.

**Table 13:** An ASIC study of optimized speed. Source: Ref. [50], Table 4.1

	CORA	KEYA	TOTA	KEYT	CRIT	K/C	THRU	T/A
MARS	690	2245	2935	1740	567	3.1	225	0.07
RC6	741	901	1643	2112	627	3.4	203	0.12
RIJN	518	93	612	57	65	0.9	1950	3.18
SERP	298	205	503	114	137	0.8	931	1.85
TWOF	200	231	431	16	324	0.1	394	0.91

CORA = area (in units of 1000 gates) for encryption/decryption core.

KEYA = area (in units of 1000 gates) for key scheduling.

TOTA = total area (in units of 1000 geates).

KEYT = key setup time, in nanoseconds.

CRIT = critical path time, in nanoseconds.

K/C = KEYT/CRIT.

THRU = throughput in megabits per second.

T/A = THRU/TOTA.

**Table 14:** A smart card study of power analysis defense. Source: Ref. [65], Table 2

	CYCU	CYCM	RAMU	RAMM	ROMU	ROMM
MARS	9425	73327	116	232	2984	7404
RC6	5964	46282	232	284	464	1376
RIJN	7086	13867	52	326	1756	2393
SERP	15687	49495	176	340	2676	9572
TWOF	19274	36694	60	696	1544	2656

CYCU = Cycle count, no masking.

CYCM = Cycle count, with masking.

RAMU = RAM in byres, no masking.

RAMM = RAM in bytes, with masking.

ROMU = ROM in bytes, no masking.

ROMM = ROM in bytes, with masking.

**Table 15:** Critical path and instruction-level parallelism. Sources: refs. [18], [45], and [98]

	Crit1	Crit2	ETHR	THR	Par	IPC
MARS	258	214	0.56	0.57	2	10
RC6	185	181	0.69	0.69	2	2
Rijndael	86	71	0.93	0.93	7	10
Serpent	556	526	0.27	0.28	3	3
Twofish	166	162	0.69	0.70	3	6

Crit1 = the first estimate of critical path (in clock cycles); taken from Ref. [45]

Crit2 = the second estimate of critical path (in clock cycles); taken from Ref. [18]

ETHR = Estimated throughput (bits per cycle) on a hypothetical VLIW processor with 5 instruction issue slots, from Ref. [18], Fig. 1.

THR = Throughput (bits per cycle) on an actual VLIW processor with 5 instruction issue slots, in feedback mode [19], slide #3.

Par = An estimate of the maximum number of processing elements that can be effectively used in parallel, taken (roughly) from Table 6 in Ref. [18].

IPC = An estimate of the maximum number of processing elements that can be effectively used in parallel; taken from Ref. [98].

**Table 16:** Software speeds (encryption): 32 bit processors (C)

	A		B		C		D		E	
	Clocks	Norm.	Clocks	Norm.	Clocks	Norm.	Mbits	Norm.	Clocks	Norm.
MARS	306	0.73	1600^a^	0.78	656	0.48	80.6	0.64	364	0.74
RC6	223	1.00	1436	0.87	318	1.00	125.9	1.00	269	1.00
RIJN	237	0.94	1276	0.98	805	0.40	52.6	0.42	362	0.74
					981	0.32	44.3	0.35	428	0.63
					1155	0.28	38.2	0.30	503	0.53
SERP			1800	0.70	1261	0.25	50.3	0.40	953	0.28
TWOF	282	0.79	1254	1.00	780	0.41	50.3	0.40	366	0.73

a The value is based on the Round 1 version of MARS (with a different key schedule from the Round 2 version).

**Table 17:** Software speeds (encryption): 32 bit processors (Java)

	F		G		H	
	kbits/s	Norm.	kbits/s	Norm.	Clocks	Norm.
MARS	19 718	0.75	3738	0.77	8840^a^	0.69
RC6	26 212	1.00	4698	0.97	6110	1.00
RIJN	19 321	0.74	4855	1.00	7770	0.79
	16 922	0.65	4664	0.96		
	14 957	0.57	4481	0.92		
SERP	11 464	0.44	1843	0.38	10 050	0.61
TWOF	19 265	0.73	1749	0.36	14 990	0.41

a The value is based on the Round 1 version of MARS (with a different key schedule from the Round 2 version).

**Table 18:** Software speeds (encryption): 64 bit processors (C and assembler)

	I^a^		J		K		L		M	
	Clocks	Norm.	Clocks	Norm.	Clocks	Norm.	Clocks	Norm.	kbit/s	Norm.
MARS	540	0.31	511	0.24	507^b^	0.97	515	0.57	63 581.1	0.73
RC6	580	0.29	490	0.26	559	0.88	428	0.68	86 522.7	1.00
RIJN	168	1.00	125	1.00	490	1.00	293	1.00	58 282.7	0.67
									49 080.1	0.57
									42 387.4	0.49
SERP	580	0.29	565/419^c^	0.22/0.30	998	0.49	854	0.34	42 174.4	0.48
TWOF	205	0.82	182	1.00	490	1.00	316	0.93	59 947.9	0.69

a The study used assembler implementations.

b The value is based on the Round 1 version of MARS (with a different key schedule from the Round 2 version).

c The encryption speed for Serpent (128 bit key) was updated in Ref. [99].

**Table 19:** Software speeds (encryption): 8 bit processors (C and assembler)

	N		O	
	Clocks	Norm.	Clocks	Norm.
MARS	34 163^a^	0.28	45 588	0.56
RC6	32 731	0.29	34 736	0.73
RIJN	9 464	1.00	25 494	1.00
SERP	126 074	0.08	71 924^b^	0.35
TWOF	26 500	0.36	31 877	0.80

a The value is based on the Round 1 version of MARS (with a different key schedule from the Round 2 version).

b The study used a non bitslice implementation of Serpent.

**Table 20:** Software speeds (encryption): 32 bit microprocessor for smart cards (ARM)

	P	
	Clocks	Norm.
MARS	9 425	0.63
RC6	5 964	1.00
RIJN	7 086	0.84
SERP	15 687	0.38
TWOF	19 274	0.31

**Table 21:** Software speeds (encryption): Digital Signal Processors (DSPs)

	Q		R	
	Clocks	Norm.	Clocks	Norm.
MARS	8 908^a^	0.34	406	0.56
RC6	8 231	0.43	292	0.78
RIJN	3 518	1.00	228	1.00
SERP	14 703	0.24	871	0.26
TWOF	4 672	0.75	308	0.74

a The value is based on the Round 1 version of MARS (with a different key schedule from the Round 2 version).

**Table 22:** Software speeds (decryption): 32 bit processors (C)

	B		C		D		E	
	Clocks	Norm.	Clocks	Norm.	Mbit/s	Norm.	Clocks	Norm.
MARS	1 580^a^	0.74	569	0.53	83.9	0.68	371	0.62
RC6	1 406	0.83	307	1.00	123.9	1.00	231	1.00
RIJN	1 276	0.91	784	0.39	57.1	0.46	358	0.65
			955	0.32	47.9	0.39	421	0.55
			1 121	0.23	41.3	0.33	492	0.47
SERP	2 102	0.55	1 104	0.28	52.0	0.42	920	0.25
TWOF	1 162	1.00	613	0.50	47.9	0.39	376	0.61

a The value is based on the Round 1 version of MARS (with a different key schedule from the Round 2 version).

**Table 23:** Software speeds (decryption): 32 bit processors (Java)

	F		G	
	Kbit/s	Norm.	kbit/s	Norm.
MARS	19 443	0.80	3 965	0.82
RC6	24 338	1.00	4 733	0.98
RIJN	18 868	0.78	4 819	1.00
	16 484	0.68	4 624	0.96
	14 468	0.59	4 444	0.92
SERF	11 519	0.47	1 873	0.39
TWOF	18 841	0.77	1 781	0.37

**Table 24:** Software speeds (decryption): 64 bit processors (C and assembler)

	I^a^		J		M	
	Clocks	Norm.	Clocks	Norm.	kbit/s	Norm.
MARS	538	0.31	527	0.24	66 608.8	0.67
RC6	493	0.34	490	0.28	98 737.7	1.00
RIJN	168	1.00	126	1.00	58 282.7	0.59
					49 368.8	0.50
					42 819.9	0.43
SERP	585	0.29	631	0.20	46 113.8	0.47
TWOF	200	0.84	182	0.69	63 581.1	0.64

a The study used assembler implementation.

**Table 25:** Software speeds (decryption): Digital Signal Processors (DSPs)

	Q		R	
	Clocks	Norm.	Clocks	Norm.
MARS	8 826^a^	0.40	400	0.67
RC6	8 487	0.41	281	0.96
RIJN	3 500	1.00	269	1.00
SERP	16 443	0.21	917	0.29
TWOF	4 328	0.81	290	0.93

a The value is based on the Round 1 version of MARS (with a different key schedule from the Round 2 version).

**Table 26:** Software speeds (key setup): 32 bit processors (C)

	B		C		D		E	
	Clocks	Norm.	Clocks	Norm.	Keys/ms	Norm.	Clocks	Norm.
MARS	4 708 (5 548)^a^	**1.00(0.85)**	4 934	**0.26**	76.9	**0.30**	2 118	**0.10**
			4 997	**0.26**	75.0	**0.30**		
			5 171	**0.25**	73.2	**0.29**		
RC6	5 186	**0.91**	2 278	**0.57**	111.1	**0.44**	1 697	**0.13**
			2 403	**0.54**	109.2	**0.44**	2 040	**0.11**
			2 514	**0.51**	105.3	**0.42**	1 894	**0.11**
RIJN	17 742 (18 886)	**0.27(0.25)**	1 289 (1 724)	**1.00(0.75)**	250.0 (162.3)	**1.00(0.65)**	215 (1 334))	**1.00(0.16)**
			2 000 (2 553)	**0.64(0.50)**	200.0 (133.3)	**0.80(0.53)**	215 (1 591)	**1.00(0.14)**
			2 591 (3 255)	**0.50(0.40)**	162.3 (107.2)	**0.65(0.43)**	288 (1 913)	**0.75(0.11)**
SERP	13 154 (12 648)	**0.36(0.37)**	6 944	**0.19**	58.8	**0.24**	1 300	**0.17**
			8 853	**0.15**	46.9	**0.19**		
			10 668	**0.12**	38.7	**0.15**		
TWOF	18 846	**0.25**	9 263	**0.14**	31.9	**0.13**	8 520	**0.03**
			12 722	**0.10**	23.3	**0.09**	11 755	**0.02**
			17 954	**0.07**	17.4	**0.07**	15 700	**0.01**

a The values are based on the Round 1 version of MARS (with a different key schedule from the Round 2 version).

**Table 27:** Software speeds (key setup): 32 bit processors (Java)

	F		G	
	kbit/s	Norm.	kbit/s	Norm.
MARS	28 680	**0.30**	2 110	**0.47**
	27 680	**0.29**	3 131	**0.70**
	26 683	**0.28**	4 131	**0.93**
RC6	45 603	**0.47**	2 233	**0.50**
	40 625	**0.42**	3 335	**0.75**
	29 683	**0.31**	4 444	**1.00**
RIJN	96 234 (56 017)	**1.00(0.58)**	1 191	**0.27**
	86 773 (48 324)	**0.90(0.50)**	1 574	**0.35**
	70 494 (39 963)	**0.73(0.42)**	1 733	**0.39**
SERP	34 729	**0.36**	487	**0.11**
	33 729	**0.35**	734	**0.17**
	31 973	**0.33**	979	**0.22**
TWOF	13 469	**0.14**	286	**0.06**
	10 556	**0.11**	327	**0.07**
	8 500	**0.09**	361	**0.08**

**Table 28:** Software speeds (key setup) 64 bit processors (C and assembler)

	I^a^		J		M	
	Clocks	Norm.	Clocks	Norm.	Keys/s	Norm.
MARS	1 797	**0.13**	1 408	**0.11**	60 975.6	**0.29**
					59 171.6	**0.28**
					57 803.5	**0.27**
RC6	1 077	**0.22**	1 581	**0.09**	147 058.8	**0.69**
RIJN	239	**1.00**	148	**1.00**	212 766 (161 290)	**1.00(0.76)**
					163 934 (125 000)	**0.77(0.59)**
					142 857 (108 696)	**0.67(0.51)**
SERP	668	**0.36**	475 / 340^b^	**0.31/0.44**	47 393.4	**0.22**
					37 878.8	**0.17**
					31 250.0	**0.15**
TWOF	2 846	**0.08**	2 445	**0.06**	31 055.9	**0.15**
					23 255.8	**0.11**
					16 420.4	**0.08**

a The study used assembler implementations.

b The key setup speed for Serpent (128 bit key) was updated in Ref. [99].

**Table 29:** Software speeds (key setup): 8 bit processors

	O	
	Clocks	Norm.
MARS	21 742	**0.47**
RC6	138 851	**0.07**
RIJN	10 318	**1.00**
SERP	147 972^a^	**0.07**
TWOF	28 512	**0.36**

a The study used a non-bitslice implementation of Serpent.

**Table 30:** Software speeds (key setup): Digital Signal Processors (DSPs)

	Q	
	Clocks	Norm.
MARS	54 427^a^	**0.49**
RC6	40 011	**0.67**
RIJN	26 642	**1.00**
SERP	28 913	**0.92**
TWOF	88 751	**0.30**

a The value is based on the Round 1 version of MARS (with a different key schedule from the Round 2 version).

## Footnotes

1 The operation of 32 bit addition is referred to simply as addition; the operation of 32 bit subtraction is referred to simply as subtraction in this report.

2 Certain commercial equipment, instruments, or materials are identified in this paper to foster understanding. Such identification does not imply recommendation or endorsement by the National Institute of Standards and Technology, nor does it imply that the materials or equipment identified are necessarily the best available for the purpose.

3 VHDL stands for VHSIC Hardware Description Language. VHSIC is yet another acronym that stands for Very High Speed Integrated Circuits.

4 Complementary Metal Oxide Semiconductor.

5 Hardware Design Language.

6 The forward direction would be used to produce the subkeys in the correct order for the encryption process, starting from the first subkey; the reverse direction would produce the subkeys in the order needed for the decryption process, starting from the last subkey.

7 Very Long Instruction Word.

## 9. Appendix A. Software Speeds for Encryption, Decryption, and Key Setup

This appendix includes Tables 16–30 and figures (Figs. 1–15) that present the *encryption, decryption*, and *key setup* performance data from various studies of the five AES finalists. Each study (identified by a letter, A-R) contains two columns.

- Column 1 contains the actual performance data (datapoints) from the study. This is usually expressed in *clock cycles* (“Clocks”); however, results measured in terms of kilobits per second (kbit/s), megabits per second (Mbit/s), etc. are marked as such. In Tables 26–30, key setup times for decryption are included in parentheses if they differ noticeably from the key setup times for encryption.
- Column 2 presents a normalized representation of the data (“Norm.”) when compared to the fastest performance within a study. This allows easier comparisons of relative algorithm performance. The fastest datapoint in a study receives a normalized value of “1.00.” The remaining normalized values are calculated as follows. Where speed is measured in clock cycles, the normalized value of a selected datapoint equals the fastest datapoint divided by the selected datapoint. Where speed is measured in bits (or keys) per second (or ms), the normalized value equals the selected datapoint divided by the fastest datapoint. For example, in study “A” in Table 16, RC6 uses the fewest number of clock cycles, and therefore has a value of 1.00 for that study. MARS has a normalized value of 223 / 306 = 0.73.
  For the key setup tables, the fastest datapoint within a study—for either encryption or decryption key setup speed—receives a normalized value of “1.00.” That datapoint is used to normalize key setup speeds for *both* encryption *and* decryption in that study. (Normalized values for the decryption key setup speeds are indicated in parentheses.) For example, in study “C” in Table 26, Rijndael—when setting up 128 bit keys for encryption—uses the fewest number of clock cycles, and therefore has a normalized value of 1.00 for that study. The key setup for 256 bit Rijndael keys for decryption has a normalized value of 1289 / 3255 = 0.40.

Studies C, D, E, F, G, and M include values for all three key sizes (128 bits, 192 bits, and 256 bits) for all five finalists. The remainder of the studies only includes datapoints for 128 bit keys. Datapoints for 128 bit keys are included in the tables below. If a source contains performance figures for 192 bit and 256 bit keys that differ noticeably from the 128 bit key performance, those datapoints are also included. In those cases, the datapoint for the 128 bit key size is listed first, followed by the 192 bit and 256 bit datapoints.

Following the tables is a series of figures (Figs. 1–15) that visually presents the normalization data for each table.

NIST is including this information to give the reader a sense of the relative performance of the finalists in different types of environments. These results are not exhaustive of Round 1 and Round 2 analysis, nor do they necessarily reflect optimum performance. Several other studies are available that summarize so-called “best-known” values. However, NIST does not include those studies in the tables below, since it is not always clear how or where such “best-known” values were obtained, or whether the best-known values are actually the best possible values. NIST decided that analyzing studies that implemented the algorithms in a relatively consistent manner, in a particular environment, with well-defined parameters would provide a better picture of the relative performance of the algorithms.

The reader should also understand that for the different studies presented below, the various contributors of the following values often used different coding techniques, programming languages, compilation tools, constraints, and amounts of encrypted data. This obviously resulted in different raw values.

In the following tables, the candidates will be abbreviated, as follows:

RIJN=Rijndael
SERP=Serpent
TWOF=Twofish

NIST does not vouch for the accuracy of data not obtained by NIST.

The studies in Tables 16–21 are identified by letter in the following list.

**Table:**

A:	Intel Pentium II, C. Source: Ref. [5], Table 1.
B:	Linux/GCC-2.7.2.2/Pentium 133 MHz MMX, C. Source: Ref. [10], Table 3.
C:	Intel Pentium III 600MHz, C. Ref. [7], §5.1, Table 6 (128 blocks).
D:	Apple G4 PowerPC, C. Ref. [8], slide 7.
E:	Intel Pentium II/III, C. Source: Ref. [41], Table 1.
F:	Intel Pentium Pro 200, w/Symantec Visual Cafe 2.5a, Java. Ref. [89], Appendix.
G:	Intel Pentium Pro 200, w/JDK 1.3, JIT, Java. Ref. [28], Appendix B.
H:	UltraSPARC-I, w/JDK1.2, JIT, Java. Ref. [9], Table 2
I:	Hewlett-Packard PA-RISC, ASM. Source: Ref. [98], Appendix A.
J:	Hewlett-Packard IA-64, C. Source: Ref. [98], Appendix A; Ref. [99].
K:	Compaq Alpha 21164a 500 MHz, C. Source: Ref. [9], Table 1.
L:	Compaq Alpha 21264, C. Ref. [95], Table 1.
M:	SGI 300 MHz R12000, C w/GCC-2.8.1. Ref. [7], §5.2, Table 2.
N:	Motorola 6805 CPU Core, C. Ref. [54], Table 3. (For MARS and RC6, there were two values presented. These two are for implementations where more than 120 bytes of RAM are available.)
O:	Z80 CPU + coprocessor. Ref. [82], Table 8.
P:	ARM CPU. Ref. [65], Table 2.
Q:	Texas Instruments TMS320c541 DSP, C. Source: Ref. [44].
R:	Texas Instruments TMS320C6201 DSP, C. Source: Ref. [97], Table 1, single-block mode.

The studies in Tables 22–25 are identified by letter in the following list.

**Table:**

B:	Linux/GCC-2.7.2.2/Pentium 133 MHz MMX, C. Source:Ref. [10], Table 3.
C:	Intel Pentium III 600MHz, C. Ref. [7], §5.1, Table 6 (128 blocks).
D:	Apple G4 PowerPC, C. Ref. [8], slide 7.
E:	Intel Pentium II/III, C. Source:Ref. [41], Table 1.
F:	Intel Pentium Pro 200, w/Symantec Visual Cafe 2.5a, Java. Ref. [89], Appendix.
G:	Intel Pentium Pro 200, w/JDK 1.3, JIT, Java. Ref. [28], Appendix B.
I:	Hewlett-Packard PA-RISC, ASM. Source:Ref. [98], Appendix A.
J:	Hewlett-Packard IA-64, C. Source:Ref. [98], Appendix A.
M:	SGI 300 MHz R12000, C w/GCC-2.8.1. Ref. [7], §5.2, Table 2.
Q:	Texas Instruments TMS320c541 DSP, C. Source:Ref. [44].
R:	Texas Instruments TMS320C6201 DSP, C. Source: Ref. [97], Table 1, single-block mode.

The studies in Tables 26–30 are identified by letter in the following list.

**Table:**

B:	Linux/GCC-2.7.2.2/Pentium 133 MHz MMX, C. Source:Ref. [10], Table 3.
C:	Intel Pentium III 600MHz, C. Ref. [7], §5.1, Table 6 (128 blocks).
D:	Apple G4 PowerPC, C. Ref. [8], slide 7.
E:	Intel Pentium II/III, C. Source:Ref. [41], Table 1.
F:	Intel Pentium Pro 200, w/Symantec Visual Cafe 2.5a, Java. Ref. [89], Appendix.
G:	Intel Pentium Pro 200, w/JDK 1.3, JIT, Java. Ref. [28], Appendix B.
I:	Hewlett-Packard PA-RISC, ASM. Source:Ref. [98], Appendix A.
J:	Hewlett-Packard IA-64, C. Source:Ref. [98], Appendix A.
M:	SGI 300 MHz R12000, C w/GCC-2.8.1. Ref. [7], §5.2, Table 2.
Q:	Texas Instruments TMS320c541 DSP, C. Source:Ref. [44].
R:	Texas Instruments TMS320C6201 DSP, C. Source: Ref. [97], Table 1, single-block mode.
